# Graphene-supported organoiridium clusters catalyze *N*-alkylation of amines *via* hydrogen borrowing reaction†

**DOI:** 10.1039/d4ra06595f

**Published:** 2024-11-04

**Authors:** Tsun-Ren Chen, Siang-Yu Chiu, Wen-Jen Lee, Yi-Siou Tsai, Yu-Sheng Huang

**Affiliations:** a Department of Applied Chemistry, National Pingtung University Pingtung City Taiwan trchen@mail.nptu.edu.tw; b Department of Applied Physics, National Pingtung University Pingtung City Taiwan

## Abstract

Graphene-supported organic iridium clusters (GSOICs) have been designed, prepared, characterized, and used for *N*-alkylation of amines *via* hydrogen borrowing reactions. Structural analysis data (including IR, XPS, TEM and EDS) show that organoiridium clusters are uniformly formed on the surface of graphene, and the grain size of GSOIC is between 1 and 3 nm. After being activated by the auxiliary ligand TMPP (tris(4-methoxyphenyl)phosphine), GSOIC showed excellent catalytic performance for hydrogen borrowing reaction, with its turnover frequency (TOF) reaching 13.67 h^−1^. Multi-cycle catalysis shows that the GSOIC/TMPP catalytic system exhibits high stability and reliability, with the turnover number (TON) of each catalytic cycle reaching 328, and the cumulative TON of 10 consecutive catalytic cycles reaching 3280. These systems exhibit excellent *N*-alkylation for a variety of substrates under one-pot conditions without the need for bases, solvents, and other additives, representing a sustainable and environmentally friendly catalytic reaction strategy.

## Abstract 2

Graphene-supported organic iridium clusters (GSOIC) exhibit excellent *N*-alkylation on various substrates under green processes.

## Graphical abstract

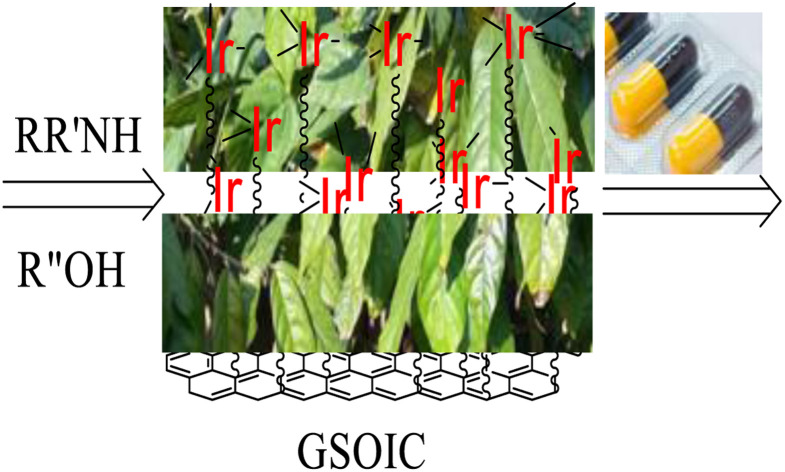

## Introduction

**Fig. 1:**
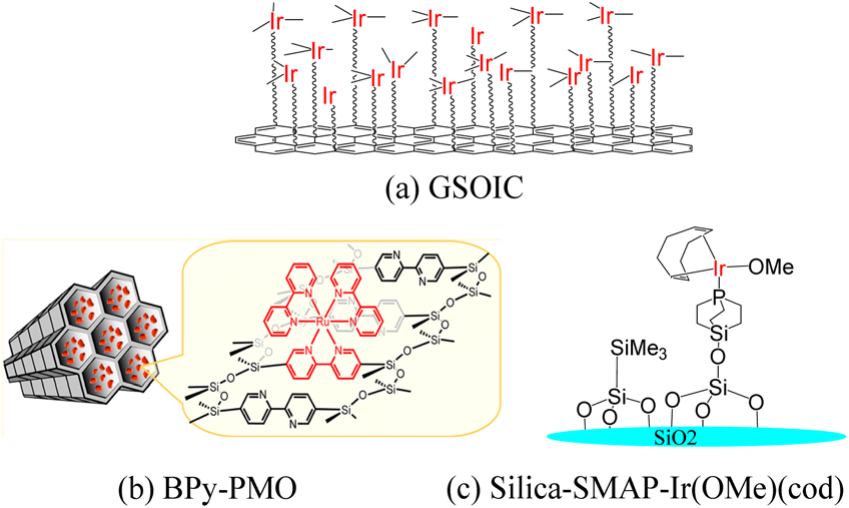
Comparison of several advanced catalytic materials: (a) graphene-supported organoiridium clusters (GSOIC), (b) metal–organic frameworks (MOFs) (BPy-PMO) (c) silica-based materials (silica-SMAP-Ir(OMe)(COD)).

Graphene is a single layer of carbon atoms tightly bound into a hexagonal honeycomb lattice. It has a high theoretical specific surface area (SSA≒2630 m^2^ g^−1^),^1^ which is much larger than the currently reported SSA of carbon black (usually less than 900 m^2^ g^−1^) or carbon nanotubes (less than 1000 m^2^ g^−1^). As a result, graphene has the highest ratio of edge atoms of any allotrope, giving graphene sheets some special chemical reactivity and which can be modified to form graphene-based materials for applications in the electronics, biological and chemical industries.^2–4^ After functionalization, some molecules can be grafted on the surface of graphene to form a structure with jungle-like molecular chains standing on the two-dimensional surface of graphene, called graphene supported molecular clusters (GSMC).^5,6^ In this report, we focus on combining GSMC with iridium to form graphene-supported organoiridium clusters (GSOIC) (Fig. 1a), including the synthesis, characterization, and applications of GSOIC. Compared with metal–organic frameworks (MOFs) formed from organic single molecules (Fig. 1b)^7^ or silica-based materials (Fig. 1c),^8^ GSOIC provides higher contact area and higher density of catalytic centers, to promote chemical reactions. In addition, the structure of GSOIC is quite stable to acids, bases and organic solvents, and the GSOIC structure will not be destroyed during the catalysis process. Moreover, the graphene surface of GSOIC has a higher affinity for organic substrates (π–π interaction between the benzene-like structure of graphene and organic molecules), which is beneficial to the catalytic frequency (turnover frequency, TOF).

**Scheme 1:**
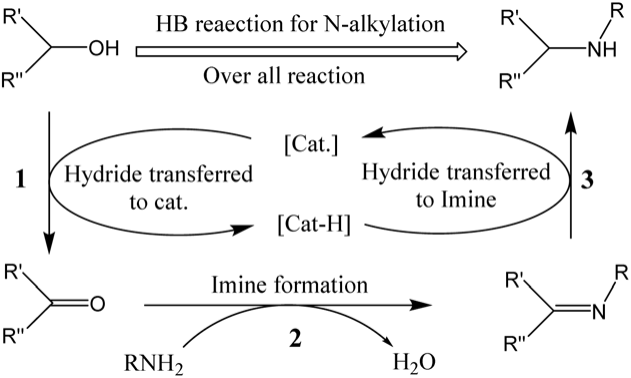
General mechanism of hydrogen borrowing strategies for amine *N*-alkylation.

GSOIC will be used for hydrogen borrowing reactions in *N*-alkylation of amines, as nitrogen-containing organic moieties are important components and play a vital role in pharmaceuticals and bioactive compounds. To date, the alkylation of amines with alkyl halides has been widely used industrially to produce nitrogen-containing molecules.^9,10^ Although quite fast, it often comes with some significant drawbacks, such as tedious purification procedures and environmentally unfriendly stoichiometric waste. To overcome the problems posed by halide-based *N*-alkylation, many studies have been conducted, including Buchwald–Hartwig coupling,^11^ hydroamination,^12^ and Ullmann reaction.^13^ In addition, a more robust and sustainable approach has also attracted attention, namely the catalytic “hydrogen borrowing (HB)” strategy, using less toxic and more readily available alcohols as alkylating agents to form new C–N bonds. Scheme 1 shows the general mechanism of the hydrogen borrowing strategy.^14,15^ The following steps are involved: (1) the two hydrogens of the alcohol are transferred to the catalyst metal, the alcohol is converted into a more reactive and highly electrophilic carbonyl compound, (2) the nucleophilic addition reaction of the amine with the carbonyl compound occurs to form an imine and eliminate water. (3) In the final stage, catalytic hydrogenation of imines occurs by transferring borrowed hydrogen to form an alkylated amine. In this strategy, the only byproduct of the process is water, and all elements of the reactants (left) have been transferred to the products (right), providing a sustainable approach with high atomic efficiency.

To date, a variety of catalysts have been discovered for the *N*-alkylation of amines using alcohols as alkylating agents, the most common of which are iridium and ruthenium complexes,^16–19^ but many other metals have also been explored.^20–24^ Many of these research reports show good to excellent catalytic ability for *N*-alkylation, but most require complex reaction conditions and difficulty in recycling used, which limits the development of applications. Here, we report a catalytic system GSOIC/TMPP for the *N*-alkylation of amines *via* a hydrogen borrowing process. These systems exhibit excellent *N*-alkylation performance for a variety of substrates under one-pot conditions without the need for bases, solvents, and other additives. The concepts presented in this report can be extended to various catalytic systems with different grafted molecules on the graphene surface to develop novel catalytic systems.

## Results and discussion

### Synthesis and characterization of GSOIC

**Scheme 2:**
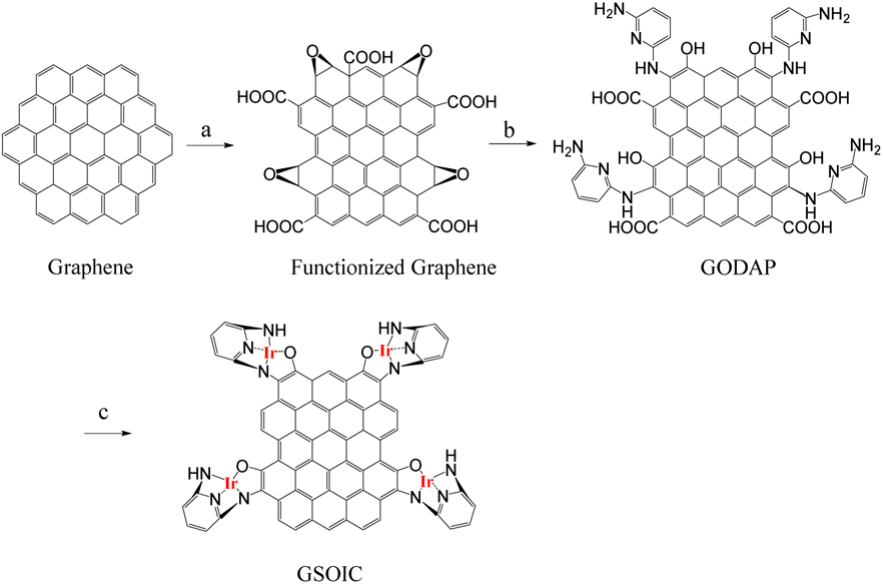
Preparation procedure of graphene supported molecular cluster GSOIC.

**Fig. 2:**
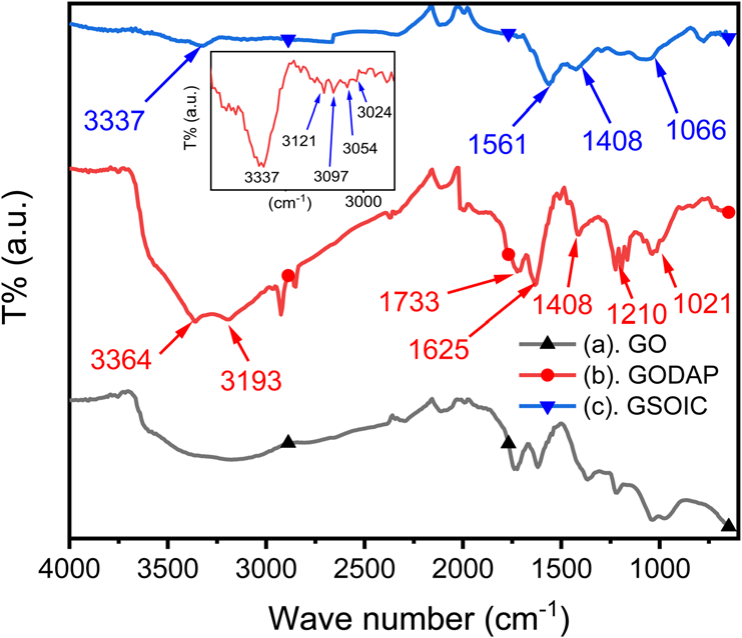
Infrared spectra of (a) GO (▲), (b) GODAP (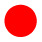) and (c) GSOIC (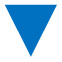).

Graphene-supported organoiridium clusters GSOIC was prepared in three steps (Scheme 2). A modified Hummers' method was used for functionalizing graphene to prepare graphene oxide (GO) (Scheme 2a).^25,26^ Under hydrothermal reaction conditions (120 °C, 96 hours), the highly reactive epoxy group of GO is attacked and ring-opened by the nucleophilic amine group of 2,6-diaminopyridine (DAP) to obtain a graphene grafted product (GODAP) (Scheme 2b). The GODAP infrared spectrum (red line in Fig. 2) has a broad absorption band between 2500 and 3700 cm^−1^, showing the presence of hydroxyl groups. In the broad absorption band between 2500 and 3700 cm^−1^, there are sharp prominent peaks at 3364 and 3193 cm^−1^, showing the characteristics of N–H and aromatic C–H. The peak at 1733 cm^−1^ indicates the presence of carbonyl groups. The peaks at 1625 and 1408 cm^−1^ are caused by aromatic ring stretching. The peaks at 1210 and 1021 cm^−1^ originate from the stretching of C–O. GODAP reacts with iridium(iii) chloride under high-pressure hydrothermal reaction conditions (140 °C, 96 h) to form GSOIC (Scheme 2c). The nitrogen atom of the 2,6-diaminopyridine residue and the hydroxyl group formed by ring opening of the epoxide are coordinated with iridium. Under this reaction condition, decarboxylation occurs, the original carboxyl groups in GODAP are removed, and the hexagonal structure of graphene is restored. The GSOIC infrared spectrum (blue line in Fig. 2) shows the presence of N–H (3337 cm^−1^), aromatic ring stretching (1561 and 1408), and C–O stretching (1066). However, the absorption bands of hydroxyl, carboxyl and carbonyl groups disappear.

**Fig. 3:**
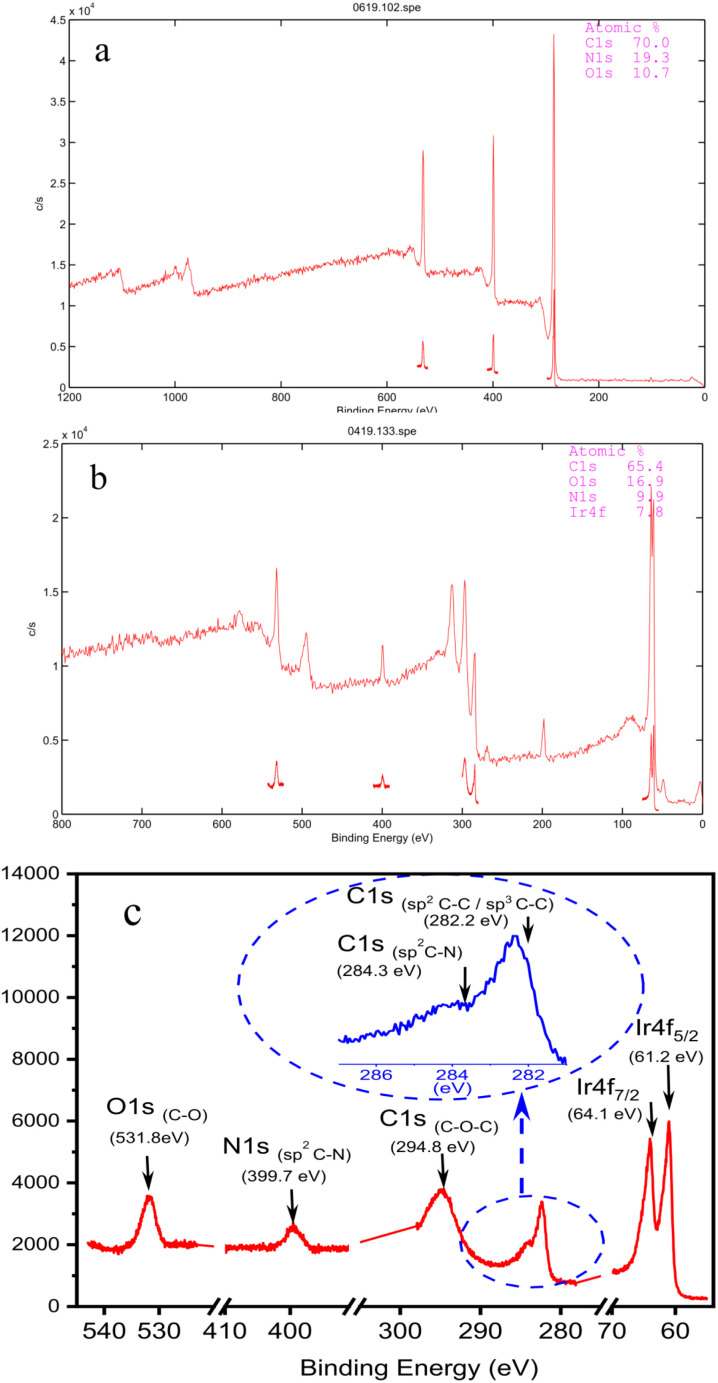
(a) Survey XPS of GODAP; (b) survey XPS of GSOIC; (c) chemical states of elements in GSOIC.

The X-ray photoelectron spectrum (XPS) of GODAP (Fig. 3a) shows that GODAP is composed of C, N and O, with atomic ratios of 70%, 19.3% and 10.7% respectively, indicating that GO is combined with DAP. The composition of GSOIC shown by XPS (Fig. 3b) is C, N, O and Ir, with atomic ratios of 65.6%, 16.9%, 9.97% and 7.8% respectively, showing that iridium is bound to GODAP. The chemical states of elements in GSOIC (Fig. 3c) shows two characteristic peaks of iridium, the Ir 4f^7/2^ peak centred at 61.06 eV and the Ir 4f^5/2^ peak centred at 64.16 eV, which are significantly higher than Ir^0^ at 60.61 respectively and 63.51 eV, which confirms that iridium should be ionic and bonded to the GODAP skeleton. The binding energies of 284.3 eV and 399.7 eV indicate C–N bonding in GSOIC, and 294.8 eV and 531.8 eV represent C–O bonding in GSOIC.^27^

**Fig. 4:**
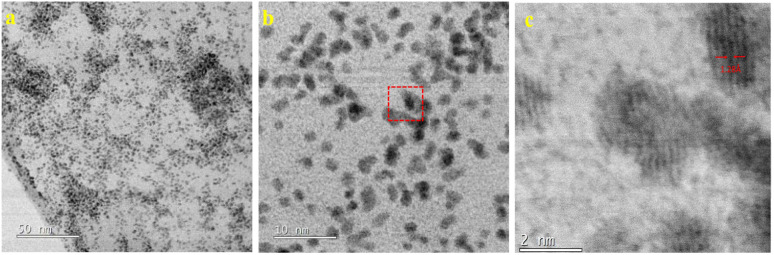
(a) Low-magnification image transmission electron microscopy (TEM) of GSOIC; (b) representative high-resolution TEM; (c) magnified fragment of b (indicated by square symbols).

**Fig. 5:**
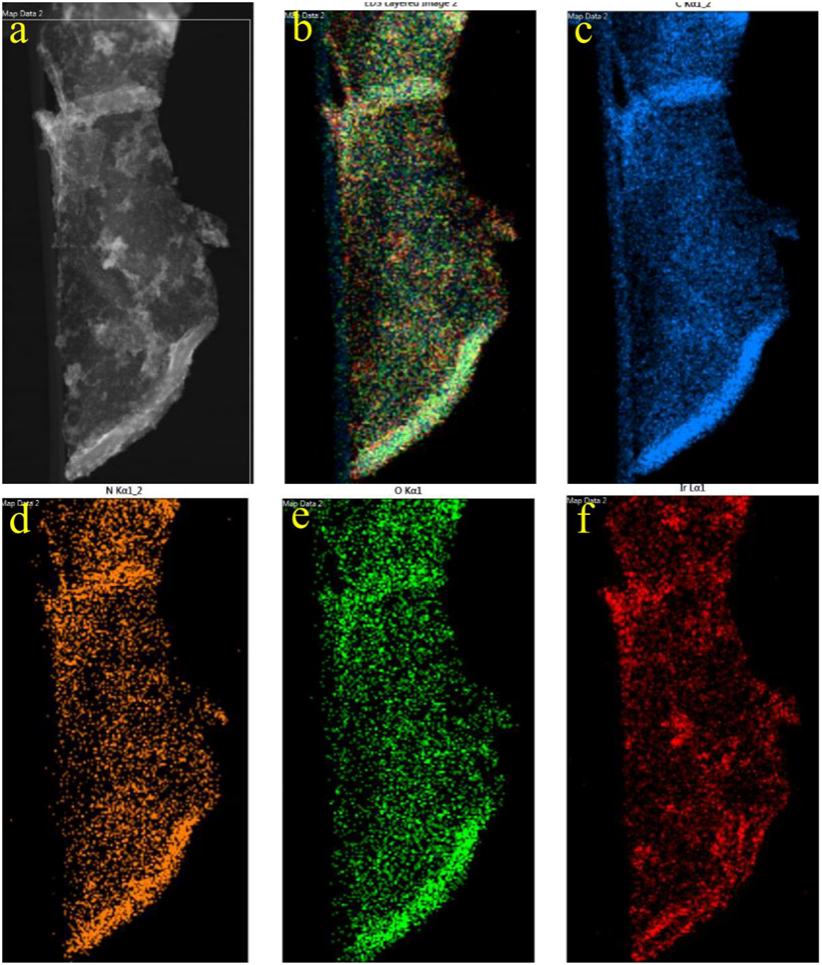
(a) Marker area for EDS element mapping; (b) overlap of various elements within the mapping area; (c–f) map of individual elements in the selected area.

Low-magnification image transmission electron microscopy (TEM) of GSOIC (Fig. 4a) shows that some substructures grow uniformly on the surface of graphene sheets. Representative high-resolution TEM (Fig. 4b) shows that the grain size of GSOIC ranges from 1 to 3 nm. Fig. 4c is an enlarged fragment of Fig. 4b (indicated by square symbols), where the lattice of graphene and iridium structures can be observed, with some carbon atoms replaced by iridium ions. The radius of iridium is about 1.28 Å, which belongs to the covalent bond of iridium. Fig. 5 shows the EDS elemental map of the TEM GSOIC image. Various elements including carbon, nitrogen, oxygen and iridium atoms are distributed in the mapping area (Fig. 5b), where carbon atoms are evenly distributed to form a skeleton structure (Fig. 5c). Nitrogen and oxygen atoms are uniformly bonded and distributed on the carbon surface (Fig. 5d and e). Last but not least, iridium atoms are bonded on the top surface of the above component structure (Fig. 5f).

### Initial catalytic studies

**Scheme 3:**
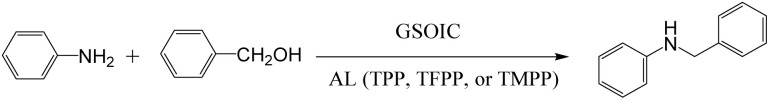
Hydrogen borrowing reactions for *N*-alkylation of aniline using catalyst GSOIC activated by triphenylphosphine derivatives.

**Fig. 6:**
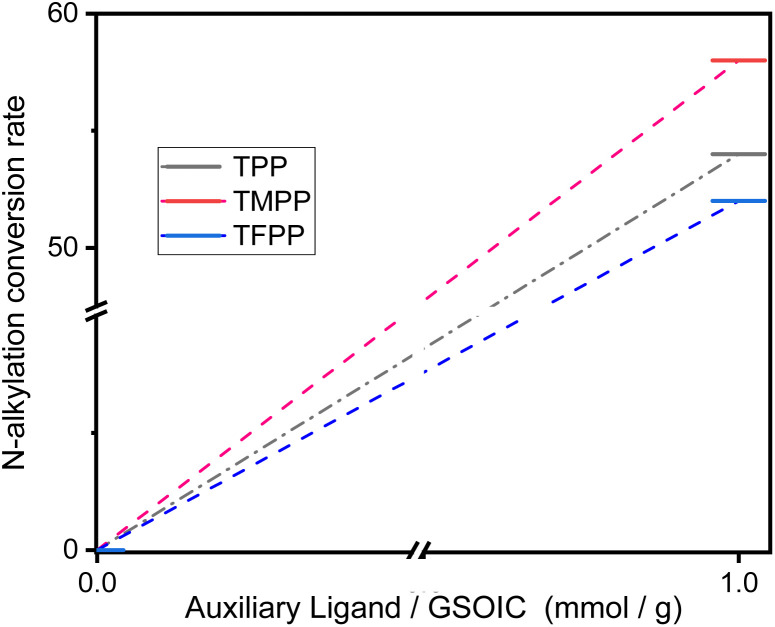
Conversion rate of aniline to *N*-benzylaniline in hydrogen borrowing reaction in GSOIC catalytic system using different triphenylphosphine derivatives as auxiliary ligands.

**Scheme 4:**
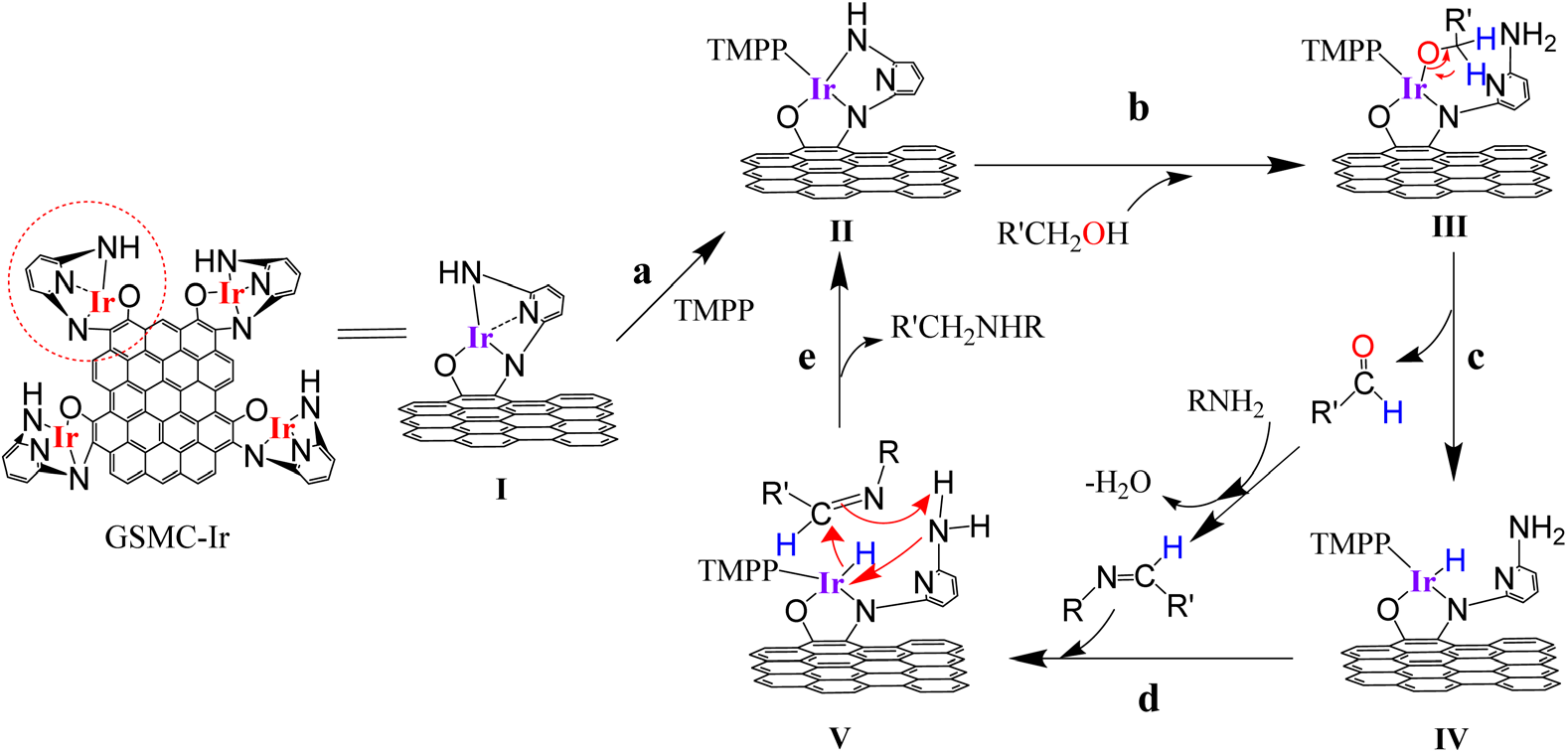
A plausible mechanism for C–N bond formation in the GSOIC/AL catalytic systems.

GSOIC is quite stable but not catalytically active, so it needs to be activated before being used in catalysis. For metastable complexes, nitrogen- or phosphorus-containing compounds are usually used as auxiliary ligands (AL),^28,29^ which can help metastable complexes release active species. We used triphenylphosphine derivatives, including triphenylphosphine (TPP), tris(4-methoxyphenyl)phosphine (TMPP) and tris(4-fluorophenyl)phosphine (TFPP) as auxiliary ligands to activate GSOIC for hydrogen borrowing reactions. *N*-alkylation of aniline was performed using benzyl alcohol as alkylating agent (Scheme 3). Preliminary test results show that when the molar ratio of amine to alcohol is 1 : 2, the alkylation efficiency is significantly better than the ratio of 1 : 1 or 1 : 3. The alkylation efficiency increases with increasing GSOIC loading. When the ratio of amine to GSOIC reaches 1 mmol : 0.002 g, the catalytic efficiency is quite excellent. Too much catalyst will lead to a decrease in alkylation efficiency and is not conducive to economic benefits. Therefore, the molar ratio of amine to alcohol was set to 1 : 2, and the ratio of auxiliary ligand to GSOIC was initially set to 0.002 mmol : 0.002 g (equal to 1 mmol : 1 g). Fig. 6 shows that when TPP, TMPP and TFPP are used as auxiliary ligands, the conversion rates of aniline to *N*-benzylaniline are 54%, 58% and 52% respectively, indicating that the triphenylphosphine derivatives with electron doner group (EDG) are beneficial to the catalytic performance of GSOIC.^30^ Based on the previous reports and preliminary mechanistic studies,^27,29–34^ a plausible mechanism is proposed for C–N bond formation in the GSOIC/AL catalytic systems (Scheme 4). Triphenylphosphine derivatives exhibit different catalytic properties, so auxiliary ligands (AL) must be involved in the reaction mechanism. According to previous studies and related literature reports, the auxiliary ligand should be the initiator of the reaction mechanism.^29,30^ In this step, the phosphorus atom of TMPP provides an electron pair to the iridium of GSOIC, resulting in the formation of a covalent Ir–P bond and the cleavage of a covalent Ir–N_py_ bond to form intermediate II (step a). Our previous studies and other literature reports indicate that the metal–alkoxide complex should form in the catalytic cycle and the borrowed hydrogen comes from the α-carbon of the alkoxide.^27,31^ In these steps, the lone pair electron of the alcohol's oxygen attacks iridium, the hydrogen of the alcohol's hydroxyl group is transferred to the terminal amino group of DAP, forming metal alkoxide complex III (step b), and then the hydride is transferred from the α-carbon to iridium to form iridium hydride complex IV and aldehyde (step c), while the released aldehyde reacts with the amine to form an imine.^31,32^ The experimental results show that the concentration of imine gradually decreases at the end of the reaction, indicating that the imine is drawn into the catalytic cycle and consumed, in which the imine formed in the previous step is adsorbed onto the catalyst. Transfer the hydride from iridium to the electrophilic carbon of the imine, forming the *N*-alkylation product (R′CH_2_NHR) and regenerating intermediate II (steps d and e).^31,33,34^

**Fig. 7:**
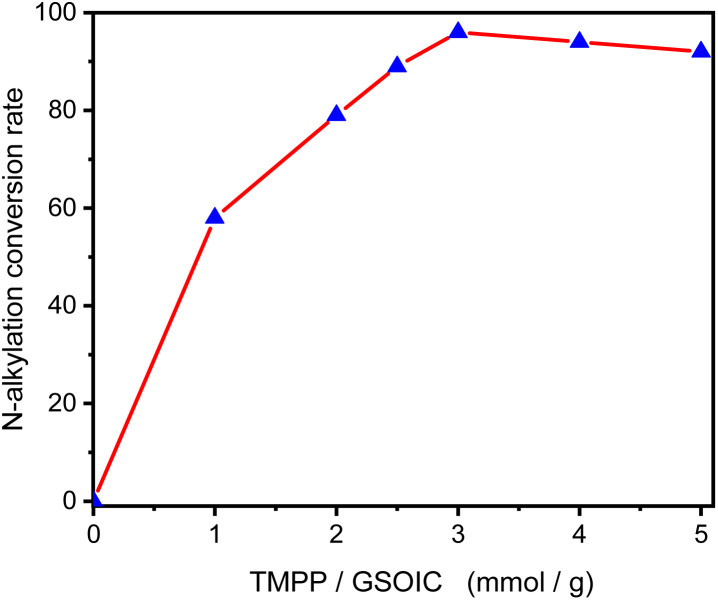
*N*-Alkylation of aniline with benzyl alcohol catalyzed by different TMPP/GSOIC ratios.

Then, we evaluated the effect of the catalytic system formed by different ratios of TMPP and GSOIC on the hydrogen borrowing reaction. Fig. 7 shows that under reaction conditions without TMPP, no *N*-alkylation product was observed, and then the conversion of aniline to *N*-benzylaniline increased with the increasing ratio of TMPP to GSOIC. When the ratio of TMPP to GSOIC increased to 3 (mmol g^−1^), a maximum conversion rate of 96% was observed, after which the conversion rate gradually decreased as the ratio of TMPP to GSOIC increased. Interestingly, XPS elemental analysis shows that the atomic ratio of iridium in GSOIC is 7.8%, equivalent to 2.91 mmol Ir/GSOIC. In other words, an equimolar amount of TMPP can fully stimulate the activity of GSOIC, while an excess of TMPP will inhibit the reaction.

**Fig. 8:**
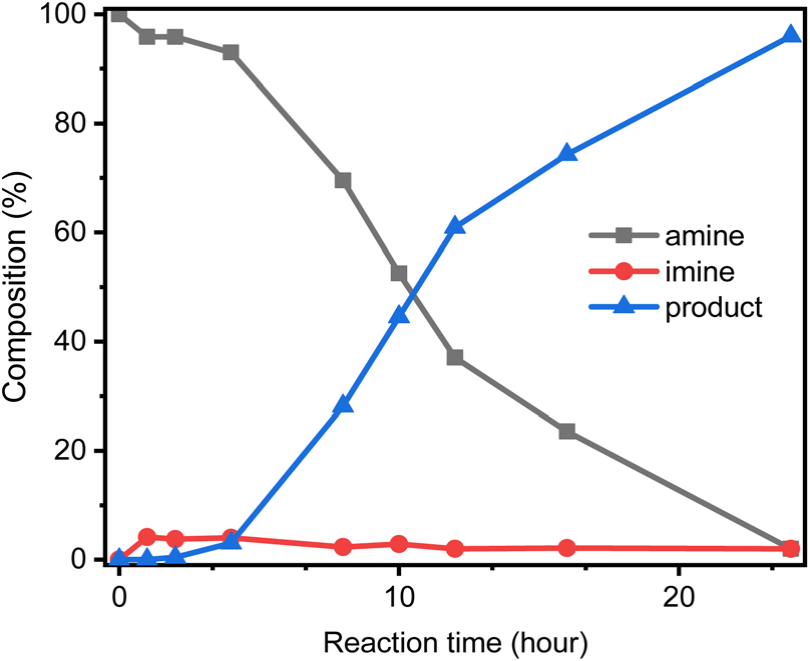
Reaction progress of *N*-alkylation of aniline. Aniline (1 mmol) react with benzyl alcohol (2 mmol), and catalyst GSOIC/TMPP (0.002 g/0.006 mmol); composition distribution of nitrogen-containing products was determined by GC-MS.

Fig. 8 shows the composition of the reaction mixture during the *N*-alkylation of aniline. Within 0 to 2 hours of reaction, imine increased significantly, with an average increase of 4%, but the product *N*-benzylaniline was not obvious (less than 1%). Since the lower imine concentration is not conducive to the adsorption of imine to iridium hydride complex IV, the transfer of hydride from iridium hydride complex IV to imine is relatively low at this stage. Since then, the product *N*-benzylaniline has increased significantly, and the intermediate imine has almost remained at 3–4%. At this stage, aniline is converted to imine, which is rapidly converted to the product *N*-benzylaniline through hydride transfer. In the later stages of the reaction, aniline is almost exhausted (less than 2%), imine is also reduced to 2%, and the product *N*-benzylaniline is higher than 95%. At this stage, the supply of imine decreases, but the transfer of hydride from iridium hydride complex IV to imine keep going.

### Reliability of GSOIC/TMPP catalytic system in catalytic cycle

**Fig. 9:**
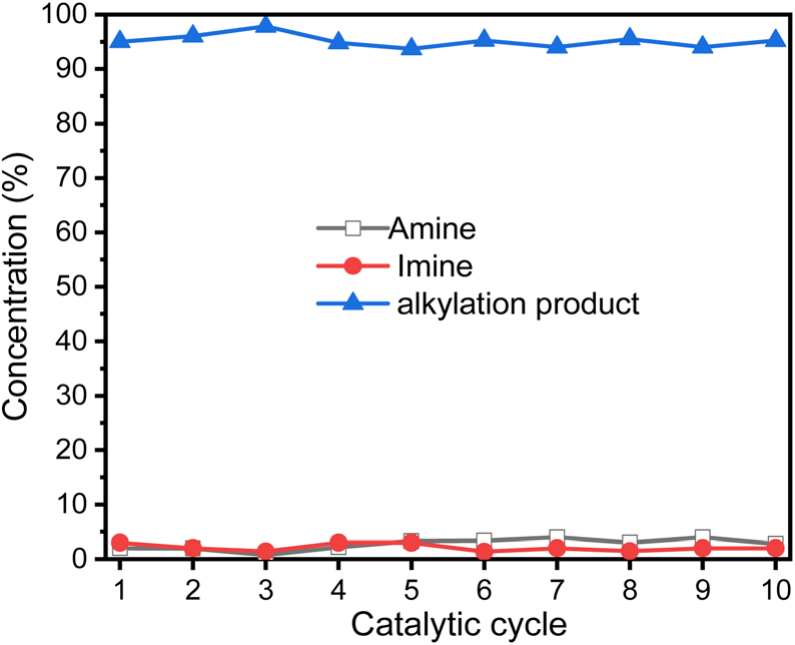
Yield of *N*-benzylaniline in ten catalytic cycles of GSOIC/TMPP catalytic system.

Ideally, heterogeneous catalysts can be used in continuous production process, and the products can be easily separated from the reaction mixtures. Therefore, we studied ten catalytic cycles of the GSOIC/TMPP system for the *N*-alkylation of aniline to evaluate the activity and stability of GSOIC during long-term catalysis. For each cycle, 1 mmol of amine was mixed with 2 mmol of alcohol, 0.001 g of catalyst GSOIC, and 0.003 mmol of TMPP. The reaction was carried out at 160 °C for 24 h, and the recovered GSOIC was separated by centrifugation and used for the next round of reaction. The yield of the first cycle was 95.0%. The yield of the second cycle was 96.1%, an increase of 1.1%. Fortunately, the yield remained between 94.0% and 97.0% after the second cycle, and the yield in the tenth cycle reached 95.2%, indicating that the catalyst is quite stable under the reaction conditions and will not encounter deactivation (Fig. 9).

**Fig. 10:**
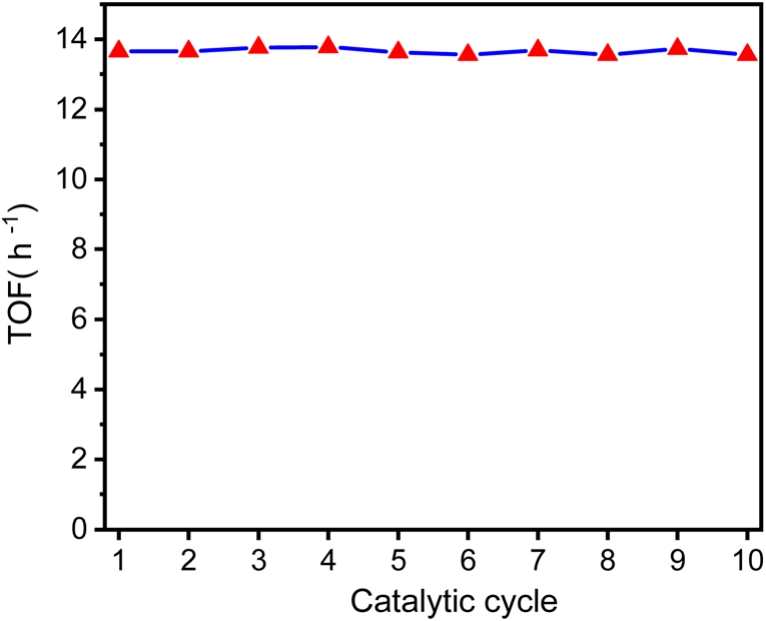
The TOF of the catalytic cycle of converting aniline into *N*-benzylaniline shows that the catalytic ability of the GSOIC/TMPP catalytic system for the hydrogen borrowing reaction is quite stable.

**Fig. 11:**
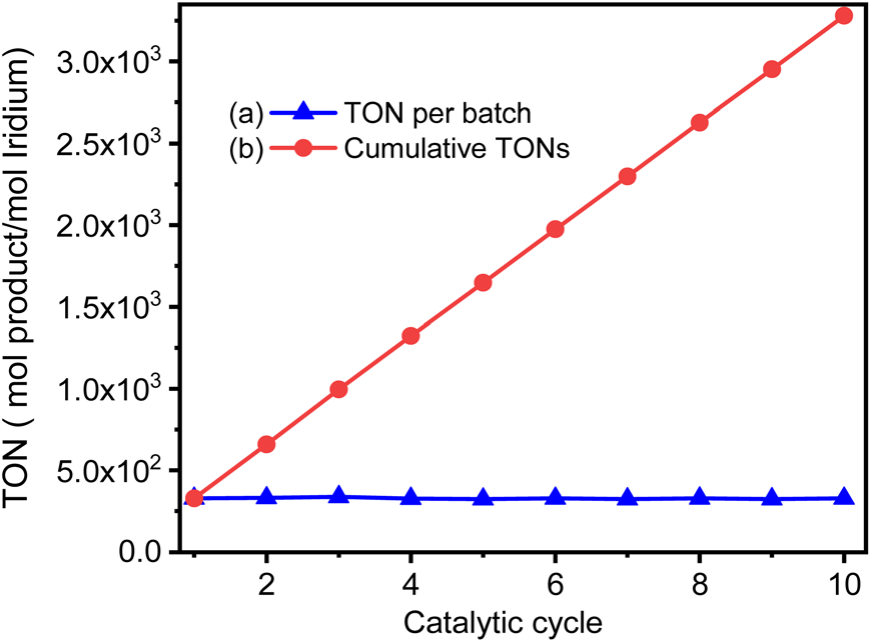
The TON of the catalytic cycles for the transformation of aniline into *N*-benzylaniline; (a) TON of each cycle, (b) accumulated TON, for the cycle n the accumulated TON was calculated by 
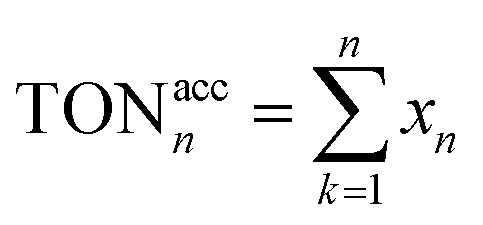
.

Fig. 10 shows the turnover frequency (TOF, mol_produced_/mol_iridium_ × h^−1^) of ten cycles of the GSOIC/TMPP catalytic system for the *N*-alkylation reaction of aniline, where the TOF of the first cycle is 13.65 h^−1^, the average TOF in 10 cycles is about 13.65 h^−1^, and the TOF in the last cycle is 13.68 h^−1^. The results show that the TOF of the hydrogen borrowing reaction of the GSOIC/TMPP system is quite stable and does not lose activity during catalytic cycles. The turnover number (TON, mol_produced_/mol_iridium_) and cumulative TON in 10 cycles are shown in Fig. 11. The TON of the first cycle is 327.59, and the TON of other cycles is similar to the first cycle. The average TON over the 10 cycles is approximately 327.66, and the TON for the last cycle is 164.14 (Fig. 11a); therefore, TON accumulates steadily (Fig. 11b). The cumulative TON of 10 cycles exceeds 3280, which means that when 172 g GSOIC is used in 10 cycles, the cumulative TON of *N*-alkylation products will reach 3280 mol, that is, 1 kg GSOIC can produce 9834 mol (1694 kg) of product, showing high productivity. Table 1 lists comparative data for the *N*-alkylation of aniline using previously reported catalysts. In comparison to these literature, the GSOIC/TMPP catalytic system does not require solvents, bases, or other stoichiometric reagents, making it an environmentally sustainable solution. In addition, the catalytic ability of this catalytic system is better than other catalytic systems, which is beneficial to practical applications.

**Table 1:** *N*-Alkylation of aniline with benzyl alcohol under varying conditions^a^

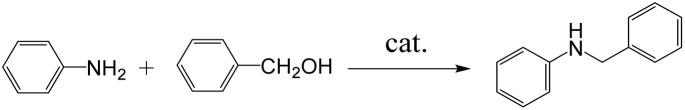					
#	Catalyst	Base/solvent temp. (°C)	Yield	TON	Ref.
1	(NNN)–NiCl_2_	NaO^*t*^Bu/toluene 140	47	2340	35
2	Ni(COD)_2_	KOH/neat 140	2	100	36
3	[NiCl_2_ + 2 phen]	KO^*t*^Bu/toluene 130	25	1250	37
4	Hf-MOF-808	No/*o*-xylene 140	85	7	38
5	(NNP)Ir (COD)	KO^*t*^Bu/dig 150	92	10	39
6	(NNP)Ru(COD)	KO^*t*^Bu/toluene 110	98	20	40
7	Co_1_–N_3_P_1_	KO^*t*^Bu/toluene 140	98	860	41
8	Ni/*θ*-Al_2_O_3_	No/*o*-xylene 144	99	800	42
9	SACs	Py/toluene 150–200	95	170	43
10	GSOIC/TMPP	No/neat 160	95	3280	This report

a (1) No: no base. (2) Neat: no solvent. (3) Dig.: diglyme 3. Py: pyridine.

### Substrate scope

We then explored the scope of *N*-alkylation substrates for coupling various amines and alcohols using the GSOIC/TMPP catalytic system, and the results are shown in Table 2. When benzyl alcohol, *p*-anisyl alcohol, and 4-chlorobenzyl alcohol were used as alkylating agents to alkylate aniline, the isolated yields of amine products 2a, 2b, and 2c were 93%, 92%, and 90%, respectively. Anilines with electron-withdrawing groups, such as 4-chloroaniline, can be alkylated well with benzyl alcohol, *p*-anisyl alcohol, and 4-chlorobenzyl alcohol to afford the corresponding amines 2d, 2e, and 2f in yields as high as 88%, 90%, and 78%, respectively. Anilines with electron-donating groups, such as *p*-anisidine, can also be alkylated well with substituted benzyl alcohols to afford the corresponding amines 2g, 2h, and 2i in yields of 90%, 92%, and 84%, respectively. Heteroaromatic compounds can also be successfully *N*-alkylated *via* a hydrogen borrowing reaction in the GSOIC/TMPP catalytic system. For example, *N*-alkylation of indole with benzyl alcohol and *p*-anisyl alcohol gave the corresponding products 2j and 2k in yields of 87% and 83%, respectively. Next, we studied the reaction models of pyrrolidine and piperazine with benzyl alcohol and *p*-anisyl alcohol, and obtained the corresponding products *N*-benzylpyrrolidine (2l), *N*-(4-methoxybenzyl)pyrrolidine (2m), benzylpiperazine (BZP, 2n) and (4-methoxybenzyl)piperazine (MOBZP, 2o), with yields of 85%, 83%, 90% and 91%, respectively. Among them, *N*-benzyl pyrrolidine derivatives are being evaluated for the treatment of Alzheimer's disease (AD),^44^ while BZP and MOBZP are antidepressant medication antihelminthic agents.

**Table 2:** *N*-Alkylation of amines with alcohols catalyzed by GSOIC/TMPP^a^

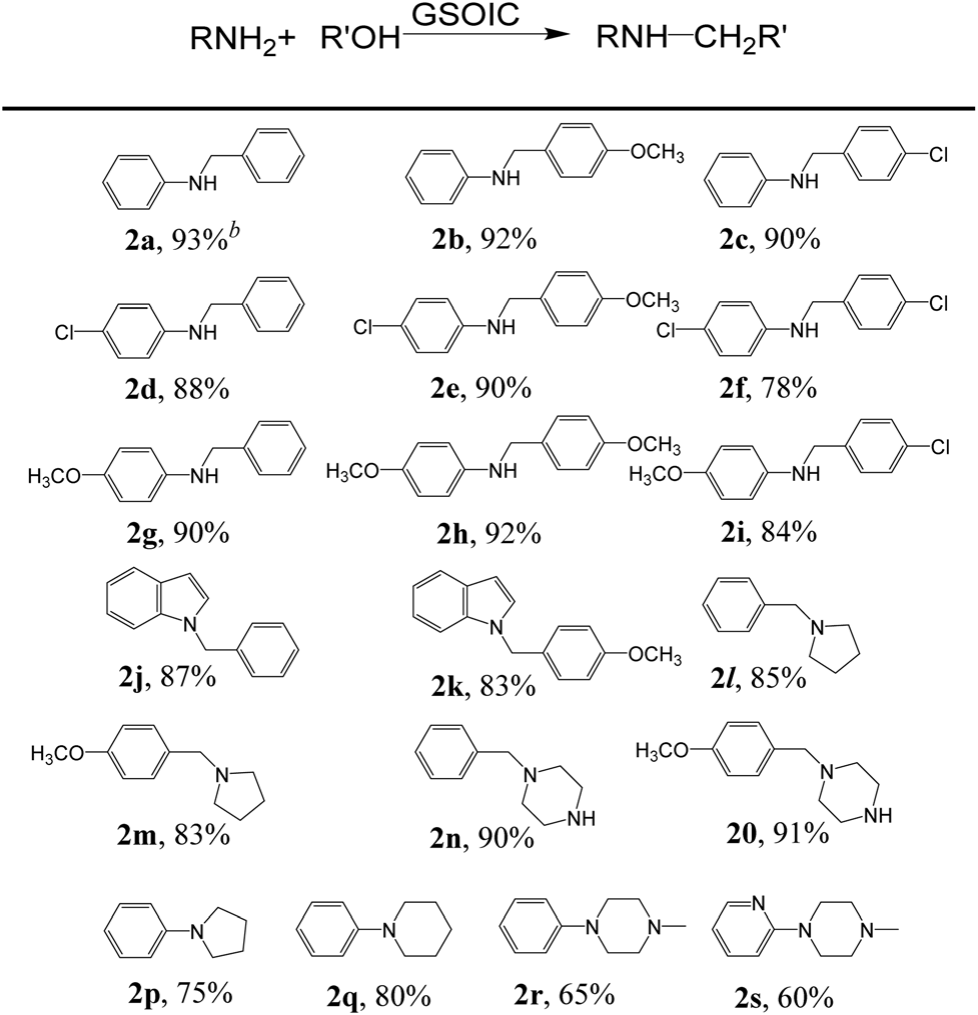

a Reaction of 1 mmol amine or nitrogen-containing compound with 2 mmol alcohol catalyzed by GSOIC/TMPP (0.002 g/0.006 mmol).

b Isolated yields.

Importantly, the heterocyclic structure, which is crucial in drugs, can be constructed through intermolecular reactions and intramolecular cyclization. For example, in the GSOIC/TMPP catalytic system, aniline reacts with 1,4-butanediol or 1,5-pentanediol to form the cyclized five-membered cyclic amine 2p and the six-membered cyclized product 2q, with yields of 75% and 80% respectively. Further, *N*-methyldiethanolamine reacts with aniline or 2-aminopyridine to produce *N*-methyl-*N*-phenylpiperazine (2r) and *N*-methyl-*N*-pyridylpiperazine (2s), with yields of 65% and 60% respectively. Among them, pyridylpiperazine derivatives are known to be effective and selective α2-adrenergic receptor antagonists, and phenylpiperazine derivatives are medicines, such as Antrafenine, Bifeprunox, Ciprofloxacin, Dropropizine, and Elopiprazole, *etc.*

### Synthetic applications

**Scheme 5:**
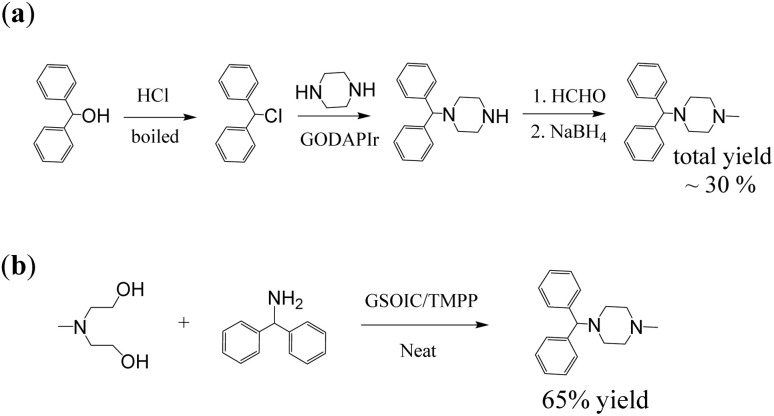
Synthesis of cyclizine: (a) previous report, (b) our case.

N-heterocycles are important structures in biochemistry and pharmaceutical compounds and are often constructed through complex steps. The use of GSOIC/TMPP catalytic system can greatly simplify the synthesis process. For example, the above-mentioned derivatives of *N*-benzylpyrrolidine (2l and 2m), benzylpiperazine (2n and 2o), phenylpiperazine (2r) and pyridylpiperazine (2s) can be prepared in one step. In addition, we used GSOIC/TMPP catalytic system to synthesize cyclizine, which has been included in the World Health Organization Essential Medicines List for the treatment and prevention of nausea, vomiting and dizziness caused by motion sickness.^45^Scheme 5a shows the traditional production process for preparing cyclizine.^46^ This route requires at least three steps with an overall yield of 30%, in which a large amount of harmful and expensive chemicals must be used, and toxic and ecologically unfavorable substances are released into the environment. In the case of Scheme 5b, we use an intermolecular cyclization reaction to prepare cyclizine *via* one-step catalysis. *N*-methyldiethanolamine (MDEA) was mixed with diphenylamine and the catalyst GSOIC/TMPP in a reactor, reacted at 160 °C for 24 hours under a nitrogen atmosphere, then cooled the reactant and filtered out the catalyst. The filtrate was purified by column chromatography, yielding an isolated yield of 65%, which is a very good yield compared to known methods.^27,47,48^

## Experimental

### Materials and methods

Iridium chloride and crystalline graphite were purchased from Seedchem Co and SHOWA Co, respectively. All other chemicals were purchased from Acros and used as received. Silica aluminum foil with fluorescent indicator 254 nm (from Merck) was used for thin layer chromatography (TLC). Use SD fine silica gel (60–120 mesh) for column chromatography, with dichloromethane and hexane gradient as the mobile phase.

### Preparation of grafted graphene (GODAP)

Graphene oxide (GO) is synthesized from graphite powder through a modified Hummers' method.^25,26^ In a flask with a stirring bar, 0.5 g of synthesized GO was dispersed in a mixed solvent (ethyl ethanol : water = 1 : 1, v/v) (15 mg mL^−1^), ultrasonicated for 40 min, and then 15 mmol of 2,6-diaminopyridine was added to the reaction mixture and heated in an oil bath (90 °C) for 60 min. Transfer the above reaction mixture to a hydrothermal reactor, put the hydrothermal reactor containing the reaction mixture into an oven, and react at 120 °C for 96 hours. The reaction was then filtered, washed with pure water and dried to obtain 0.73 g product GODAP.

### Preparation of graphene-supported molecular cluster GSOIC

In a flask with a stirring bar, 0.5 g of GODAP was dispersed in a mixed solvent (ethyl ethanol : water = 3 : 1, v/v) (10 mg mL^−1^), treated with ultrasonic for 40 minutes, and then 1.67 mmol of anhydrous iridium(iii) chloride was added to the reaction mixture and stirred for 60 minutes. Transfer the above reaction mixture to a hydrothermal reactor, put the hydrothermal reactor containing the reaction mixture into an oven, and react at 140 °C for 96 hours. The reaction was then filtered, washed with pure water and dried to obtain 0.65 g product GSOIC.

### Characterizations

NMR spectra were measured on a Bruker AVIIIHD-600 MHz or a Mercury 300 MHz NMR spectrometer. The infrared spectra were recorded on Agilent Technologies Model Cary 630 FTIR instruments. Mass spectra were taken with a Finnigan/Thermo Quest MAT 95XL instrument with electron impact ionization for organic compounds or fast atom bombardment for metal complexes. Transmission electron microscopy (TEM) images were obtained using a JEOL JEM-ARM200FTH microscope, operated at 80 kV and equipped with a cold field emission gun (CFEG), spherical-aberration corrector, and high-angle annular dark field (HAADF) detector. The TEM capabilities include a point image resolution of 0.19 nm, lattice image resolution of 0.10 nm, information limit of 0.10 nm, bright-field lattice image resolution of 0.136 nm, and dark-field lattice image resolution of 0.08 nm. Energy-dispersive X-ray spectroscopy (EDS) was also performed in conjunction with the TEM.

### General procedure for *N*-alkylation reaction

In the presence of the catalyst GSOIC/TMPP (0.002 g GSOIC and 0.006 mmol TMPP), 1 mmol of amine was mixed with 2 mmol of alcohol in a Schlenk tube (without solvent or base) and the reaction was heated to 160 °C for 24 hours and monitored by GCMS. The residue was purified by column chromatography using dichloromethane/*n*-hexane (10–100%) as eluent to obtain pure product. The desired coupling products are fully characterized by ^1^H, ^13^C NMR and MS spectroscopy.

### Reliability of catalytic systems in catalytic cycles

For each cycle, 1 mmol of amine was mixed with 2 mmol of alcohol, 0.001 g of catalyst GSOIC, and 0.003 mmol of TMPP in a Schlenk tube. The reaction was carried out at 160 °C for 24 hours. After cooling, 2 mL of dichloromethane were added to the reaction mixture, and the catalyst was separated by centrifugation. The clean supernatant was analyzed by GC-MS to identify product composition. Then, 1 mmol aniline, 2 mmol benzyl alcohol, and 0.003 mmol TMPP were added to the Schlenk tube containing the recovered GSOIC for the next catalytic cycle.

### Intermolecular cyclyzation to synthesize cyclizine

In a Schlenk tube, 1 mmol 2-diphenylamine was mixed with 2 mmol *N*-methyldiethanolamine (MDEA), 0.006 mmol TMPP and 0.002 g catalyst GSOIC, and reacted at 160 °C for 24 hours under nitrogen. Then, the reaction mixture was cooled and treated with a mixed solvent of triethylamine (TEA)/*n*-hexane/dichloromethane (DCM) with a volume ratio of 1 : 40 : 60. Then, use TEA/methanol/DCM mixed solvent with a volume ratio of 1 : 10 : 90 as the eluent to obtain pure product. The isolated yield of cyclizine was 65%. The desired product was fully characterized by ^1^H, ^13^C NMR and MS spectra.

## Conclusions

In summary, we successfully demonstrated an unprecedented graphene-supported organoiridium cluster (GSOIC) for hydrogen borrowing reactions. The structure of GSOIC has been fully characterized by infrared spectroscopy, X-ray photoelectron spectroscopy (XPS), transmission electron microscopy (TEM), and EDS elemental mapping. The catalytic system GSOIC/TMPP shows excellent *N*-alkylation catalytic ability, with high TOF (up to 13.67 h^−1^) and high TON (accumulated TON in 10 cycles exceeds 3280). The catalytic system GSOIC/TMPP can couple various amines and alcohols into corresponding molecules (including heteroaromatics, heterocyclic compounds and drugs) with yields ranging from 60% to 98%. This catalytic system provides a low-loss, low-waste, and high-atom-economic method for constructing drugs or other target molecules.

## Data availability

Data supporting this study are included in the ESI.†

## Conflicts of interest

There are no conflicts to declare.

## Supplementary Material

RA-014-D4RA06595F-s001
