# Supplementary material for: Graphene-supported organoiridium clusters catalyze N-alkylation of amines via hydrogen borrowing reaction
Source: RSC Adv. 2024 Nov 4;14(47):35163–71. doi: 10.1039/d4ra06595f (PMC11533416; doi:10.1039/d4ra06595f)
Supplement: RA-014-D4RA06595F-s001 [file RA-014-D4RA06595F-s001.pdf]

Supplementary Information (SI) for RSC Advances.
This journal is © The Royal Society of Chemistry 2024

Supporting Information

Graphene-Supported Organoiridium Clusters Catalyze N-alkylation of Amines via Hydrogen Borrowing Reaction

Tsun-Ren Chen,*[a] Siang-Yu Chiu,[a] Wen-Jen Lee,[b] Yi-Siou Tsai [a] and Yu-Sheng Huang [a]

[a]Department of Applied Chemistry, National Pingtung University, Pingtung City,Taiwan.

[b]Department of Applied Physics, National Pingtung University, Pingtung City, Taiwan

*Corresponding Author: Tsun-Ren Chen
E-mail: trchen@mail.nptu.edu.tw

Characterization of the Catalyzed Products

N-benzylaniline *(2a)*. ^{1}H NMR ($CDCl_3$, 600MHz) δ (ppm) = 7.33-7.36 (m, Ar, 4H), 7.17-7.24 (m, Ar, 1H), 7.15-7.16 (m, Ar, 2H), 6.70 (t, Ar, 1H), 6.62-6.63 (d, Ar, 2H, JHH = 7.8Hz), 4.32 (s, CH2, 2H), 4.02 (br. s, NH, 1H). ^{13}C NMR ($CDCl_3$, 150MHz) δ (ppm) = 148.1, 139.3, 129.2, 128.6, 127.5, 127.2, 117.5, 112.8, 48.2. HRMS (ESI/APCI): *m/z* =184.1124 g/mol, calc'd. for $C_{13}H_{14}N$: 184.1121 g/mol. FTIR IR (ATR): ν = 3418(w), 3030 (w), 1597 (s), 1500 (s), 1455 (s), 1262 (m), 746(s) , 693(s) cm^{-1}

N-(4-methoxybenzyl)aniline *(2b)*. ^{1}H NMR ($CDCl_3$, 600MHz) δ (ppm) = 7.26-7.28 (d, JHH = 8.4Hz, 2H, Ar), 7.15-7.24 (m, 2H, Ar), 6.85-6.87 (m, 2H, Ar), 6.62 (t, 1H, Ar), 6.61 (d, 2H, Ar), 4.23 (s, NH, 1H), 3.77(s, 3H). .^{13}C NMR ($CDCl_3$, 150MHz) δ (ppm) = 158.8, 148.1, 131.3, 129.2, 128.7, 117.4, 113.9, 112.8, 55.3, 47.8. HRMS (ESI/APCI): *m/z* = 214.1225 g/mol, calc'd. for $C_{14}H_{16}NO$ $[M]^+$: 214.1226 g/mol. FTIR IR (ATR): ν = 3395 (w), 3000 (w), 2836 (w), 1604(m), 1507 (s), 1463(m),1239(s) ,1172(m), 1030(m), 820(m) , 746(m) cm^{-1}.

N-(4-chlorobenzyl)aniline (2c). ^{1}H NMR ($CDCl_3$, 600MHz) δ (ppm) = 7.28 (s, 3H, Ar), 7.14-7.16 (m, 2H, Ar), 6.59-6.70 (m, 1H, Ar), 6.58-6.59 (m, 2H, Ar), 4.29 (d, 2H, JHH = 5.4Hz), 4.03 (br. s, NH, 1H). ^{13}C NMR ($CDCl_3$, 150MHz) δ (ppm) = 147.7, 137.9, 132.8, 129.2, 128.7, 128.6, 117.7, 112.8, 47.5. HRMS (ESI/APCI): *m/z* = 218.0736 g/mol, calc'd. for $C_{13}H_{13}NCl$ $[M]^+$: 218.0731 g/mol. FTIR IR (ATR): ν = 3418 (w), 3022 (w), 2813 (w),

1604(m), 1515 (s),1463(m),805(m) ,738 (s), 686(s) cm^{-1}.

***N*-benzyl, *N*-(4-chlorophenyl)amine (2d) :** ^{1}H NMR (d-chloroform, 600MHz) = 7.24-7.33 (m, Ar, 4H), 7.08-7.09 (m, Ar, 2H), 6.52-6.53 (m, Ar, 2H), 4.61 (s, 1H), 4.28-4.29 (d, CH2 ,2H, JHH = 6.0Hz), 4.05(br. s, NH, 1H). ^{13}C NMR (d-Chloroform, 150MHz) = 146.6, 138.9, 129.0, 128.9, 128.6, 127.5, 127.3, 127.0, 126.5, 122.0, 113.8 ,54.4, 48.3. HRMS (ESI/APCI) m/z = 217.6898, Calculated for $C_{13}H_{12}NCl^{+}$ $[M]^{+}$: 217.6929g/mol. FTIR IR (ATR): ν = 3388(w), 3030 (w), 2850 (w), 1597(m), 1492 (s),1455(m),813(s) , 731 (m), 694 (m) cm^{-1}.

***N*-(4-methoxybenzyl), *N*-(4-chlorophenyl)amine (2e)**

^{1}H NMR (d-chloroform, 600MHz) =7.23-7.30 (m, 2H, Ar), 7.17-7.22 (m, 4H, Ar), 6.70-6.76 (m, 2H, Ar),, 4.51 (s, 2H), 3.00 (s, -OCH_3, 3H). ^{13}C NMR (d-Chloroform, 150MHz) = 149.7, 139.0 129.1, 128.5, 126.8, 126.6, 116.4, 112.2, 56.5, 38.4. HRMS (ESI/APCI): m/z = 247.7092, Calculated for $C_{14}H_{14}NOCl^{+}$ $[M]^{+}$: 247.7183 g/mol. FTIR IR (ATR): ν = 3388 (w), 3007 (w), 2836 (w), 1604(m), 1500 (s),1463(m),1239(s) ,1172 (m), 813 (s) cm^{-1}.

***N*-(4-chlorobenzyl), *N*-(4-chlorophenyl)amine (2f)**

^{1}H NMR (d-chloroform, 600MHz) = 7.33-7.35 (m, 2H, Ar), 7.22-7.25 (m, 2H, Ar), 6.92--6.98 (m, 2H, Ar), 6.61-6.69 (m, 2H, Ar), 4.35-4.36 (d, JHH = 9Hz, 2H), 4.33 (s, 1H,NH). ^{13}C NMR (d-Chloroform, 150MHz) = 152.2, 150.6 138.9, 136.5, 128.6, 127.3, 124.5, 116.7,

114.4, 112.2, 47.8. HRMS (ESI/APCI): m/z = 252.28,08 Calculated for $C_{13}H_{11}NCl_2^+$ $[M]^+$: 252.1380 g/mol. FTIR IR (ATR): ν = 3388 (w), 3022 (w), 2858 (w), 1597(m), 1500 (s),1089(m), 806 (s) cm^{-1}.

***N*-benzyl, *N*-(4-methoxyphenyl)amine (2g):** ^{1}H NMR (d-chloroform, 600MHz) = 7.31-7.33 (m, Ar, 4H), 7.23-7.25 (m, Ar, 1H), 6.75-6.76 (d, Ar, 2H), 6.58-6.59 (d, Ar, 2H), 4.26 (s, CH2, 2H), 3.72 (s, $-OCH_3$, 3H).^{13}C NMR ($CDCl_3$, 150MHz) δ (ppm) = 152.1, 142.4, 139.6, 128.5, 127.5, 127.1, 114.8, 114.0, 55.8, 49.2. HRMS (ESI/APCI): m/z = 213.1598, Calculated for $C_{14}H_{15}NO^+$ $[M]^+$: 213.2731g/mol. FTIR IR (ATR): ν = 3321 (w), 3030 (w), 2836 (w), 1604(m), 1507 (s), 1455(m), 1231(m), 1030 (m), 820 (m), 731 (m) , 701 (m) cm^{-1}.

***N*-(4-methoxybenzyl), *N*-(4-methoxyphenyl)amine (2h)**

^{1}H NMR (d-chloroform, 600MHz) =7.23-7.25 (m, 4H, Ar), 7.10-7.22 (m, 1H, Ar), 5.89 (s, 1H, Ar), 5.81(s, 2H, Ar), 4.30 (s, 2H), 4.05 (br. s, NH, 1H),3.71 (s, $-OCH_3$, 6H). ^{13}C NMR (d-Chloroform, 150MHz) = 161.6, 150.0, 139.1, 128.6, 127.5, 127.2, 91.6, 89.8, 55.1, 48.3. HRMS (ESI/APCI): m/z = 243.2897, Calculated for $C_{15}H_{17}NO_2^+$ $[M]^+$: 243.2984g/mol. FTIR IR (ATR): ν = 3365 (w), 3000 (w), 2836 (w), 1612(m), 1507 (s),1463(m),1239(s) , 1030(s), 813(m) cm^{-1}.

***N*-(4-chlorobenzyl), *N*-(4-methoxyphenyl)amine (2i)**

^{1}H NMR (d-chloroform, 600MHz) =8.24-8.25(m, 2H, Ar), 7.31-7.34(m, 4H, Ar), 7.24-7.30

(m, 2H, Ar), 6.51-6.52 (2H, $-NCH_2Ar$), 5.55 (br. s, NH, 1H), 4.52 (s, $ArOCH_3$, 3H). ^{13}C NMR (d-Chloroform, 150MHz) = 162.2, 158.0, 139.0, 128.5, 127.4, 127.2, 110.8, 45.4. HRMS (ESI/APCI): m/z = 247.6919, Calculated for $C_{14}H_{14}NOCl^+$ $[M]^+$: 247.7182 g/mol. FTIR IR (ATR): ν = 3373 (w), 3000 (w), 2836 (w), 1612(m), 1507 (s),1463(m),1231(s) , 1030 (m), 813 (m) cm^{-1}.

***N*-benzylindole (2j)**

^1H NMR (d-chloroform, 600MHz) = 8.05 (s, Ar, 1H), 7.30-7.35 (m, Ar, 5H), 7.23-7.25 (m, Ar, 2H), 6.58-6.60 (m, Ar, 1H), 6.34-6.36 (m, Ar, 1H), 4.48 (benzylic, 2H). ^{13}C NMR (d-Chloroform, 150MHz) = 158.6, 148.2, 139.1, 137.4, 128.6, 127.3, 127.2, 113.1, 106.7, 46.3. HRMS (ESI/APCI): m/z = 207.2751, Calculated for $C_{15}H_{13}N$ $[M]^+$: 207.2691g/mol. FTIR IR (ATR): ν = 3410(m), 3030 (w), 1612 (w), 1492 (m), 1455 (m), 1336(m) , 1007(m) , 738(s) , 693(s) cm^{-1}

***N*-(4-methoxybenzyl)indole (2k)**

^1H NMR (d-chloroform, 600MHz) =7.32-7.34 (m, Ar, 2H), 7.23-7.25 (m, Ar, 2H), 7.08-7.14 (m, Ar, 2H), 6.60-6.75 (m, Ar, 2H), 6.81-6.95 (m, Ar, 2H), 6.43-6.46 (m, Ar, 2H), 4.40 (s, 1H), 4.45 (s, 1H) , 3.73 (s, 1H), 3.67 (s, 3H). ^{13}C NMR (d-Chloroform, 150MHz) = 151.8, 145.4, 141.5, 128.5, 126.7, 125.8, 116.4, 114.7, 114.4 55.7, 54.2. MS HRMS (ESI/APCI): m/z = 237.1148, Calculated for C16H15NO $[M]^+$: 237.1153 g/mol. FTIR IR (ATR): ν = 3410(m), 3000 (w), 2836 (w), 1612 (m), 1507 (s), 1455 (m), 1239(s), 1179 (m),

1030 (m), 813(m) , 738(s) cm^{-1}

N-phenylpyrrolidine (2*l*). ^{1}H NMR ($CDCl_3$, 600MHz) δ (ppm) = 7.12-7.36 (m, 5H), 3.58 (s, 2H), 2.46-2.49 (m, 4H), 1.74-1.76 (m, 4H). ^{13}C NMR ($CDCl_3$, 150MHz) δ (ppm) = 140.9, 139.1, 128.9, 128.5, 128.1, 127.5, 126.9, 126.8, 65.2, 54.1, 23.3. HRMS (ESI/APCI): *m/z* =148.2232 g/mol, calc'd. for $C_{13}H_{14}N$: 148.2234 g/mol. FTIR IR (ATR): ν = 3358 (w), 3030 (w), 2925 (m), 1649(m), 1455(m), 1201(m), 1022(s), 738 (s), 701 (s) cm^{-1}

N-(4-methoxyphenyl) pyrrolidine (2m). ^{1}H NMR ($CDCl_3$, 600MHz) δ (ppm) = 7.22 (d, Ar, 1H, JHH = 7.8Hz), 7.19 (d, Ar, 1H, JHH = 7.8Hz), 7.17 (d, Ar, 1H, JHH = 7.8Hz), 7.12 (d, Ar, 1H, JHH = 7.8Hz), 4.62 (s, 3H), 3.35(s, 2H), 2.22-2.24 (m, 4H), 1.74–1.77 (m, 4H). ^{13}C NMR ($CDCl_3$, 150MHz) δ (ppm) = 137.9, 137.3, 129.1, 128.8, 127.0, 65.1, 60.3, 54.1, 23.3. HRMS (ESI/APCI): *m/z* = 178.2486 g/mol, calc'd. for $C_{11}H_{16}NO^+$ $[M]^+$: 178.2488 g/mol. FTIR (ATR): *ν* = 3380(w), 2955(m), 1612(m), 1515(s), 1463 (m), 1246(s), 1179(s), 1029(s), 813(m), 731(w) cm^{-1}.

Benzylpiperazine (2n). ^{1}H NMR ($CDCl_3$, 600MHz) δ (ppm) = 7.23 (d, Ar, 2H, JHH = 8.0Hz), 7.29 (d, Ar, 2H, JHH = 8.0Hz), 7.17 (s, Ar, 1H), 3.71 (s, 2H), 2.65-2.69 (m, 4H), 1.84–1.77 (m, 4H). ^{13}C NMR ($CDCl_3$, 150MHz) δ (ppm) = 133.5, 130.6, 128.6, 59.2, 53.7, 23.3. HRMS (ESI/APCI): *m/z* = 176.2545 g/mol, calc'd. for $C_{11}H_{16}N_2^+$ $[M]^+$: 176.2565g/mol. FTIR (ATR): *ν* = 3291(m), 3030(w), 2858(m), 1649 (m), 1455(m), 1201(w), 1015(s), 731(s), 693(s) cm^{-1}.

(4-Methoxybenzyl) piperazine (*2o*)

^{1}H NMR (d-chloroform, 600MHz) = 7.17-7.39 (m, 4H, Ar), 3.79 (s,3H), 3.35-3.60 (m, 1H), 2.42-2.48 (m, 1H), 1.43-1.93 (m, 4H), 0.98-1.30 (m, 4H). ^{13}C NMR (d-Chloroform, 150MHz) = 142.7, 137.7, 130.0, 128.3, 127.9, 127.7, 126.8, 126.4, 63.0,53.3, 51.8. HRMS (ESI/APCI): m/z = 206.2760, Calculated for $C_{12}H_{18}N_2O^+$ $[M]^+$: 206.2819g/mol.

FTIR (ATR): *v* = 3462(w), 2940(m), 2798(m), 1612(m), 1515(s), 1440(m), 1246(s), 1179(s), 1030(s), 835(s), 813(s) cm^{-1}.

***N*-phenylpyrrolidine (*2p*):** ^{1}H NMR (d-chloroform, 600MHz) = 7.15-7.23 (m, 5H, Ar), 2.42-2.44 (m, 2H), 2.15-2.17 (m, 4H), 1.72-1.76 (m, 2H). ^{13}C NMR (d-Chloroform, 150MHz) = 148.5, 129.1, 117.0, 112.6, 43.6, 31.6, 20.2, 13.9. HRMS (ESI/APCI): m/z =147.1395, Calculated for C13H13N: 147.2169 g/mol. FTIR (ATR): *v* = 3350(s), 3037(w), 1604(s), 1500(s), 1366(w), 1276(s),1172(m), 746(s), 686(s) cm^{-1}.

***N*-phenylpiperidine (*2q*):** ^{1}H NMR (d-chloroform, 600MHz) = 7.13-7.24 (m, Ar, 2H), 6.59-6.67 (m, Ar, 1H), 6.57-6.58 (m, Ar, 2H), 3.06-3.09(m, 2H) ,1.54-1.61(m, 2H) , 1.31-1.32(m, 2H) , 1.28-1.30(m, 2H) , 0.86-0.89(m, 2H). ^{13}C NMR ($CDCl_3$, 150MHz) δ (ppm) = 148.5, 129.2, 129.1, 117.0, 112.6, 31.6, 29.5, 26.8, 22.6, 14.0. HRMS (ESI/APCI): m/z = 161.2345, Calculated for $C_{11}H_{15}N^{+}$ $[M]^{+}$: 161.2435 g/mol. FTIR (ATR): *v* = 3343(s), 2933(s), 2858(s), 1604(s), 1500(s), 1455(s), 1030(s), 746(s), 693(s) cm^{-1}.

1-methyl-4-phenyl piperazine (2r). ^{1}H NMR ($CDCl_3$, 600MHz) δ (ppm) = 7.10-7.31 (m, Ar, 2H), 7.06-7.09 (m, Ar, 1H), 6.92-6.99 (m, Ar, 2H), 3.62-3.64(m, 4H) , 2.85-2.87(m, 7H). ^{13}C NMR ($CDCl_3$, 150MHz) δ (ppm) = 139.4, 137.4, 130.2, 128.2, 128.1, 64.3, 59.8, 54.0, 23.3. HRMS (ESI/APCI): *m/z* = 177.2645 g/mol, calc'd. for $C_{11}H_{17}N_2^{+}$ $[M]^{+}$: 177.2644 g/mol. FTIR (ATR): *v* = 3343(s), 2940(m), 2843(m, 1604(s), 1500(s), 1463(m), 1269(m), 1030(s), 753(s), 693(s) cm^{-1}.

1-methyl-4-(pyridin-2-yl) piperazine (2s). ^{1}H NMR ($CDCl_3$, 600MHz) δ (ppm) =7.50 (dd, 1H), 7.45 (ddd, 1H), 7.17 – 7.25 (m, 2H), 3.98-3.99 (m, 4H), 2.35-2.37 (m, 4H), 1.97-2.00(m, 2H). ^{13}C NMR ($CDCl_3$, 150MHz) δ (ppm) = 142.6, 128.3, 127.9, 126.7, 53.3, 46.2. HRMS (ESI/APCI): *m/z* = 178.2524g/mol, calc'd. for $C_{10}H_{16}N_3^+$ $[M]^+$: 178.2525 g/mol. IR(ATR) : *v* = 3321(s), 3186(s), 2917(m), 1604(s), 1567(s), 1485(s), 1440(s), 1321(m), 1149(m), 768(s), 738(s) cm^{-1}.

Cyclizine. ^{1}H NMR ($CDCl_3$, 600MHz) δ (ppm) = 7.17-7.44 (m, 10H, Ph), 4.25 (s, 1H, N-CH), 3.33 (s, 8H, N-CH), 2.32 (s, 3H, N-CH3). ^{13}C NMR ($CDCl_3$, 150MHz) δ (ppm) = 143.9, 143.8, 128.9, 128.5, 128.9, 128.1, 77.5, 56.5, 54.5, 52.3, 45.6. HRMS (ESI/APCI): *m/z* =267.3862 g/mol, calc'd. for $C_{18}H_{23}N_2$ $[M]^+$: 267.3863 g/mol. FTIR (ATR): *v* = 3022(w), 2925(s), 2805(s), 1678(m), 1611(m), 1492(m), 1455(s), 1343(m) ,1305(m), 1283(m), 1164(s), 1134(s), 1007(s), 776(s), 746(s), 693(s) cm^{-1}.

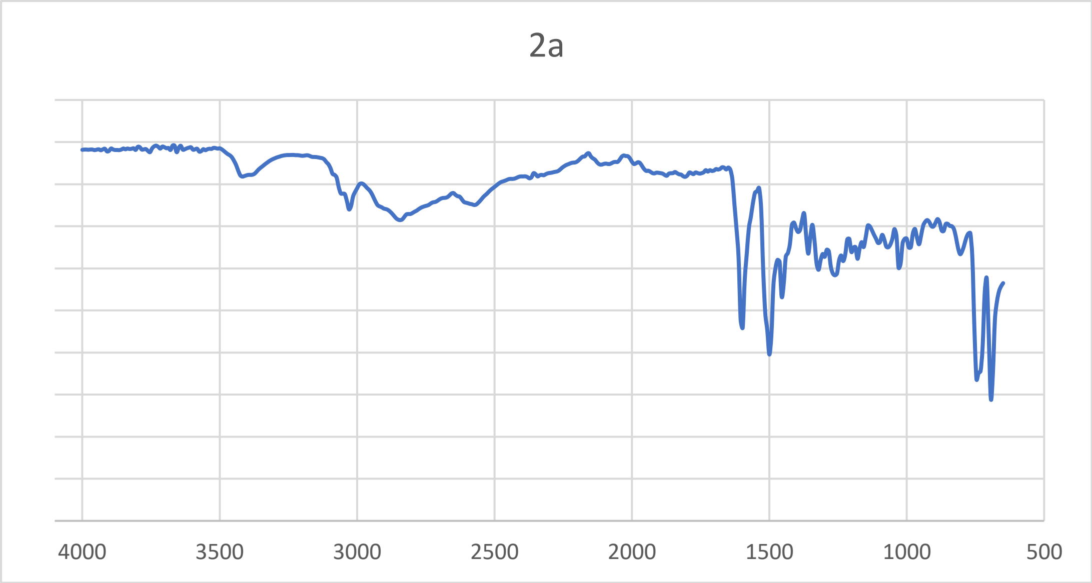

Figure S1. Infrared spectrum of 2a

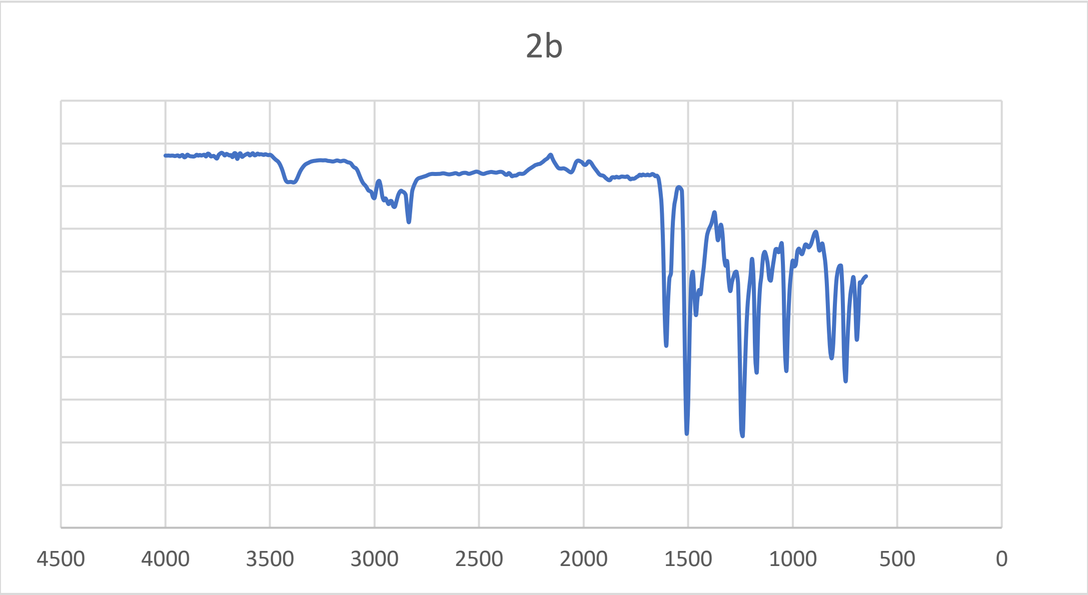

Figure S2. Infrared spectrum of 2b

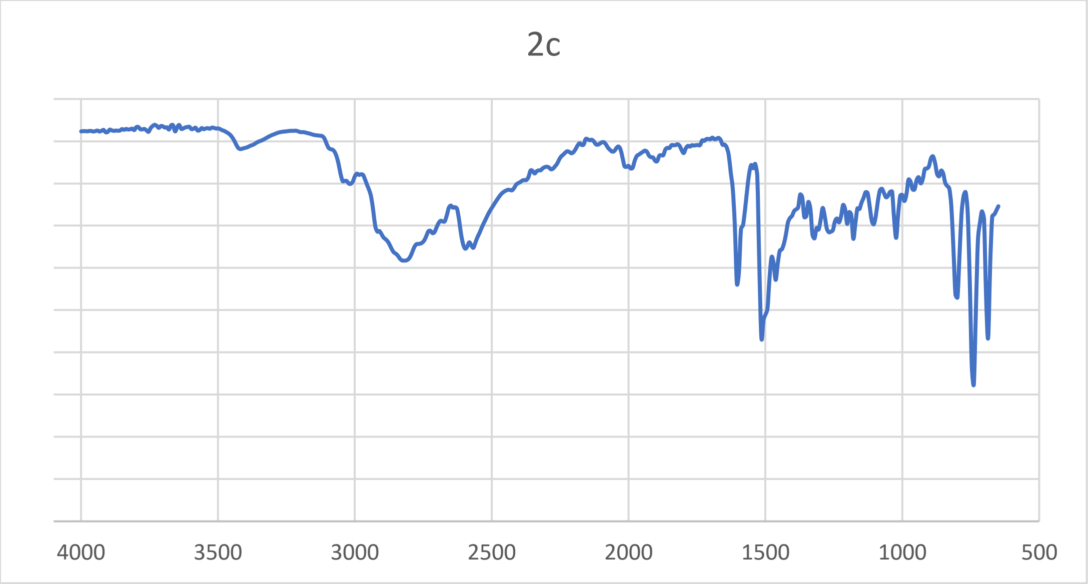

Figure S3. Infrared spectrum of 2c

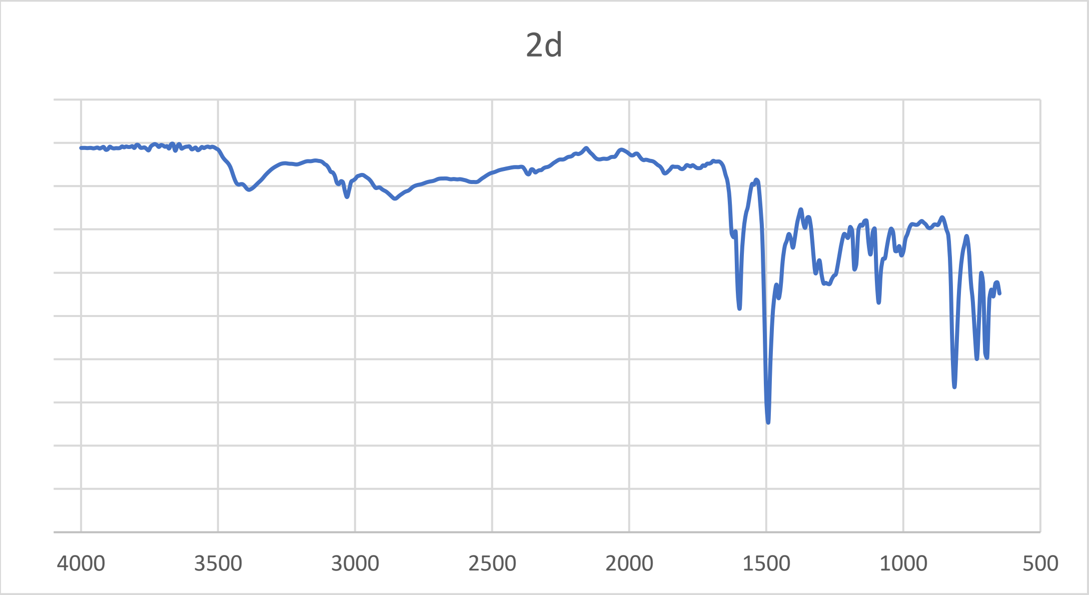

Figure S4. Infrared spectrum of 2d

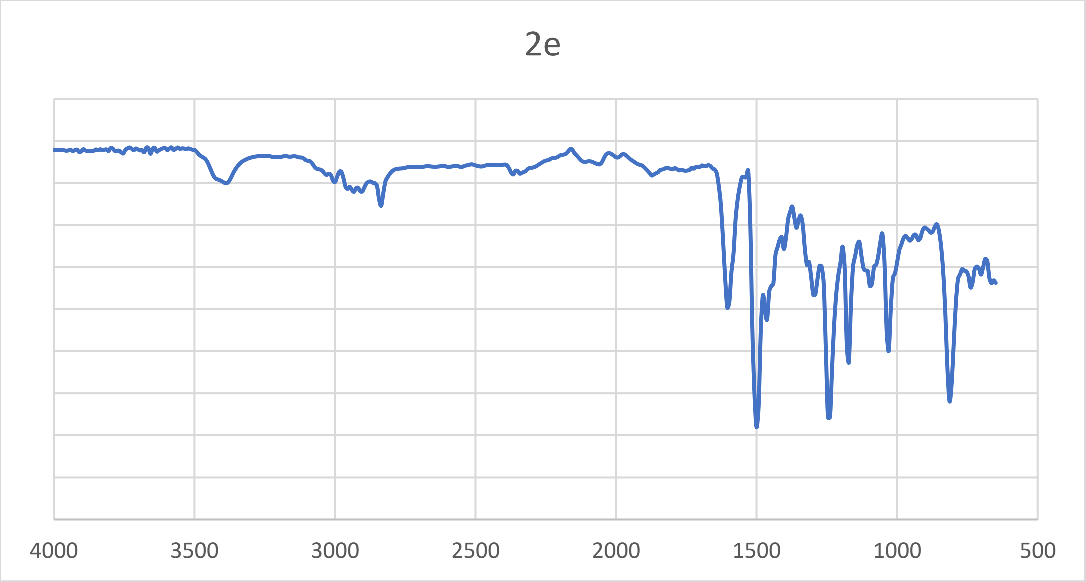

Figure S5. Infrared spectrum of 2e

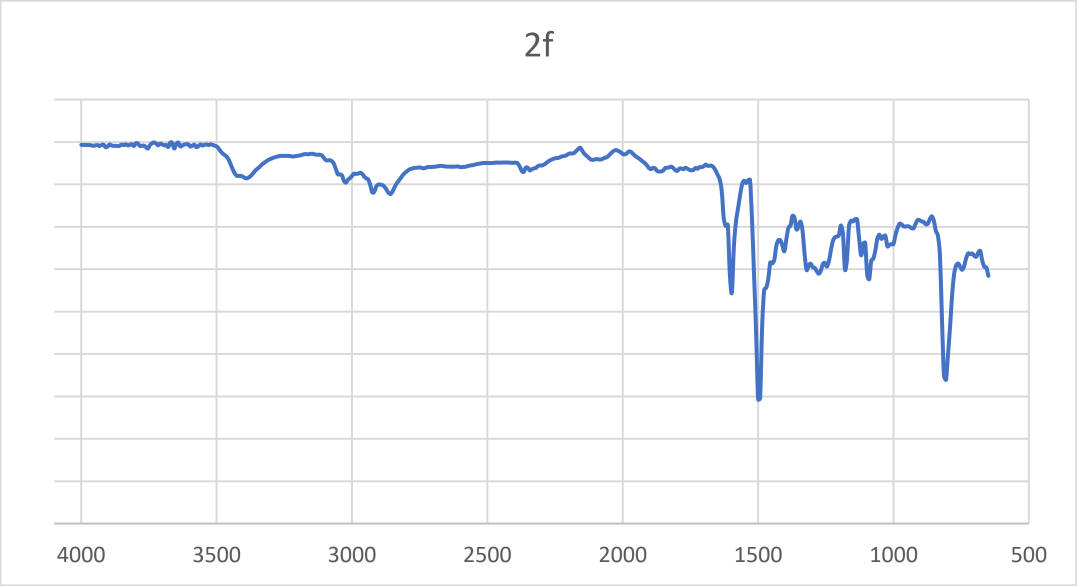

Figure S6. Infrared spectrum of 2f

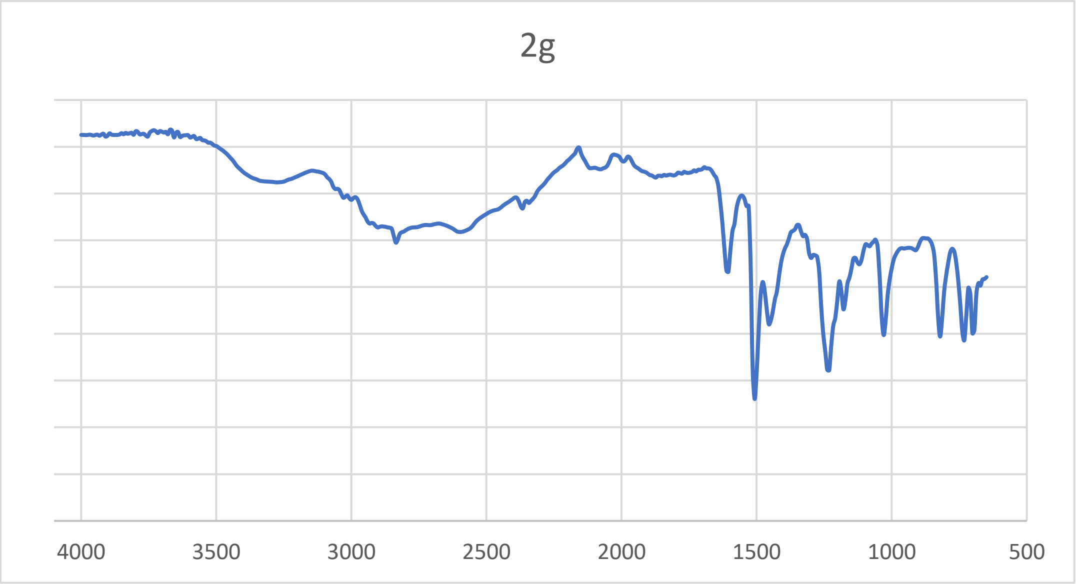

Figure S7. Infrared spectrum of 2g

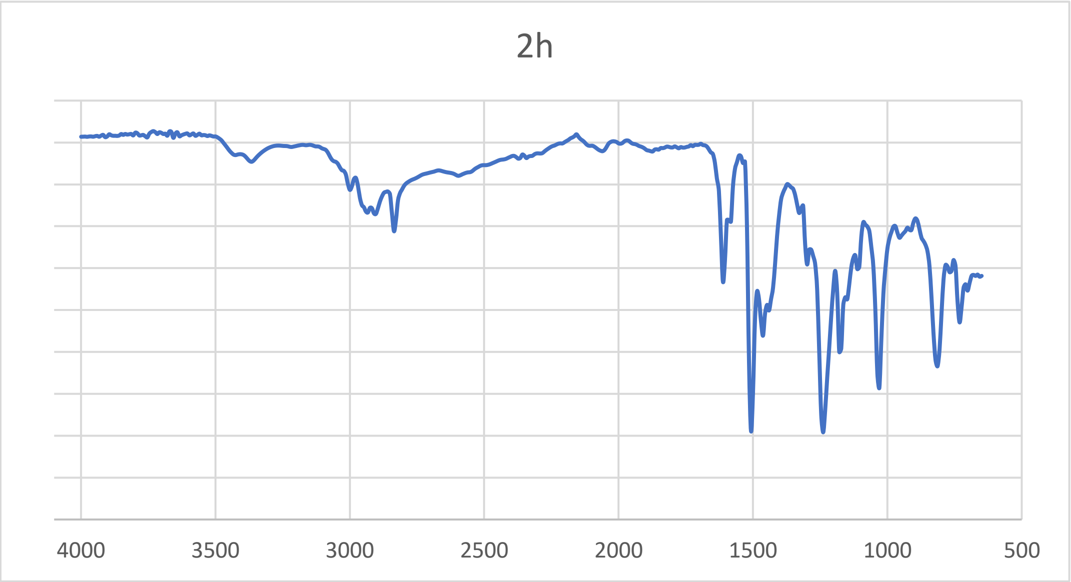

Figure S8. Infrared spectrum of 2h

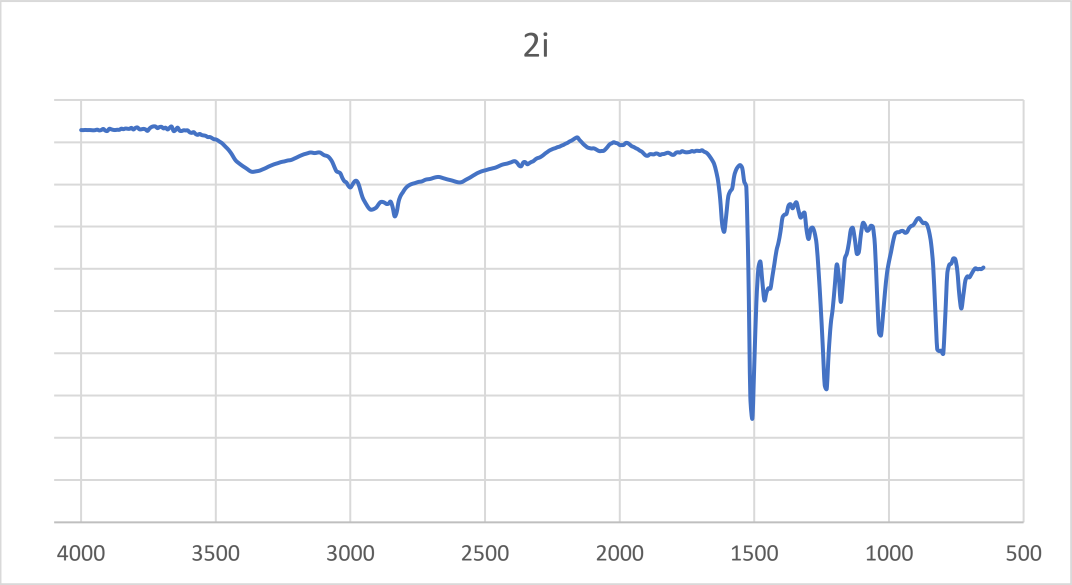

Figure S9. Infrared spectrum of 2i

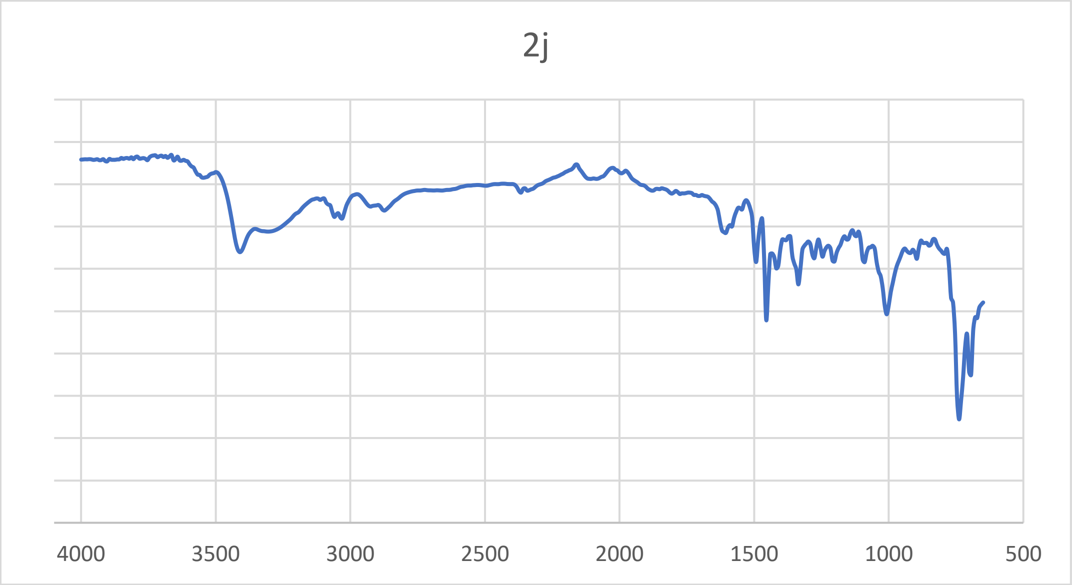

Figure S10. Infrared spectrum of 2j

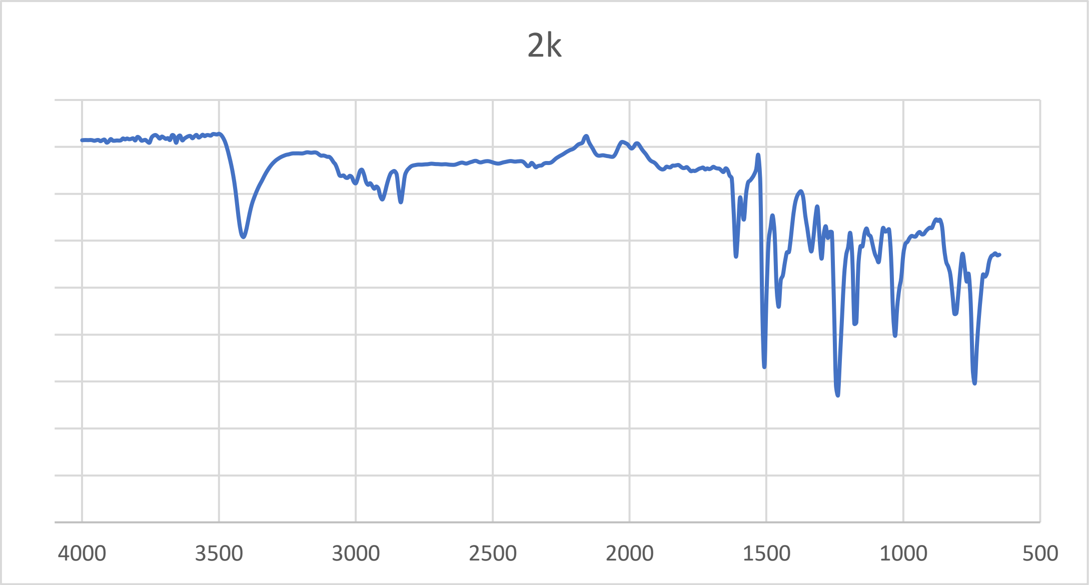

Figure S11. Infrared spectrum of 2k

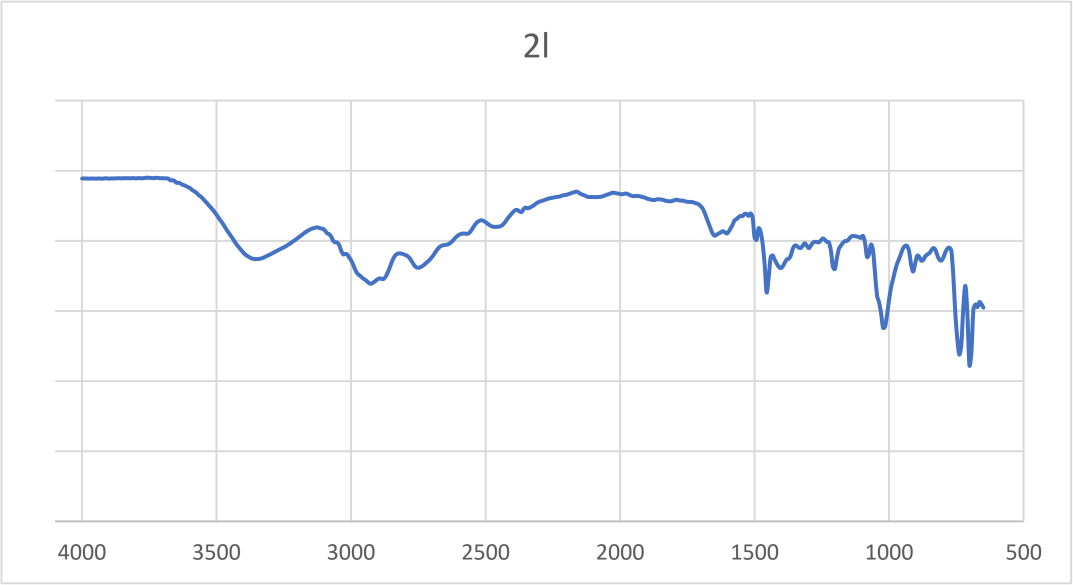

Figure S12. Infrared spectrum of 2l

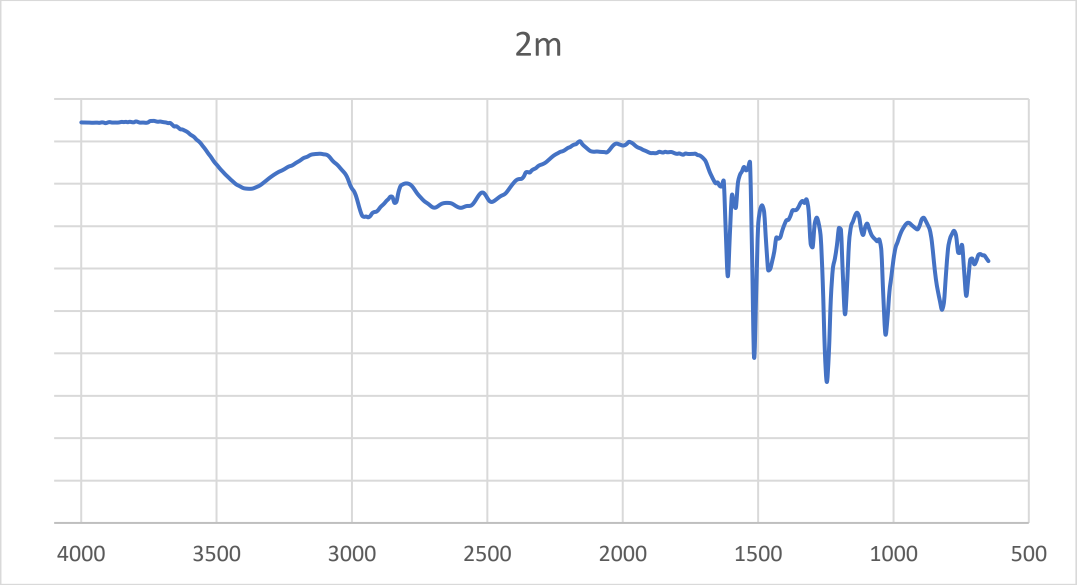

Figure S13. Infrared spectrum of 2m

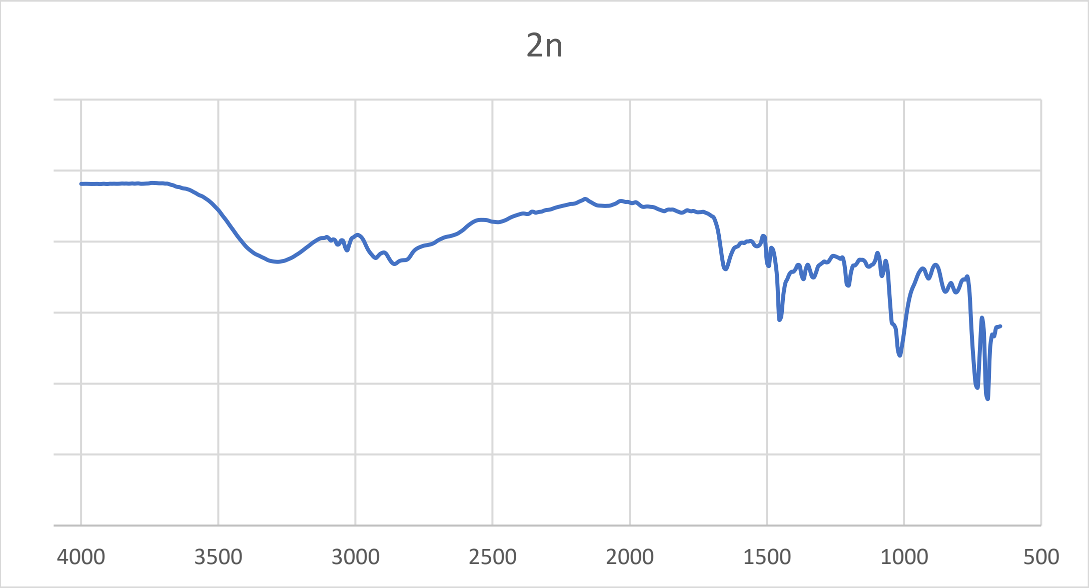

Figure S14. Infrared spectrum of 2n

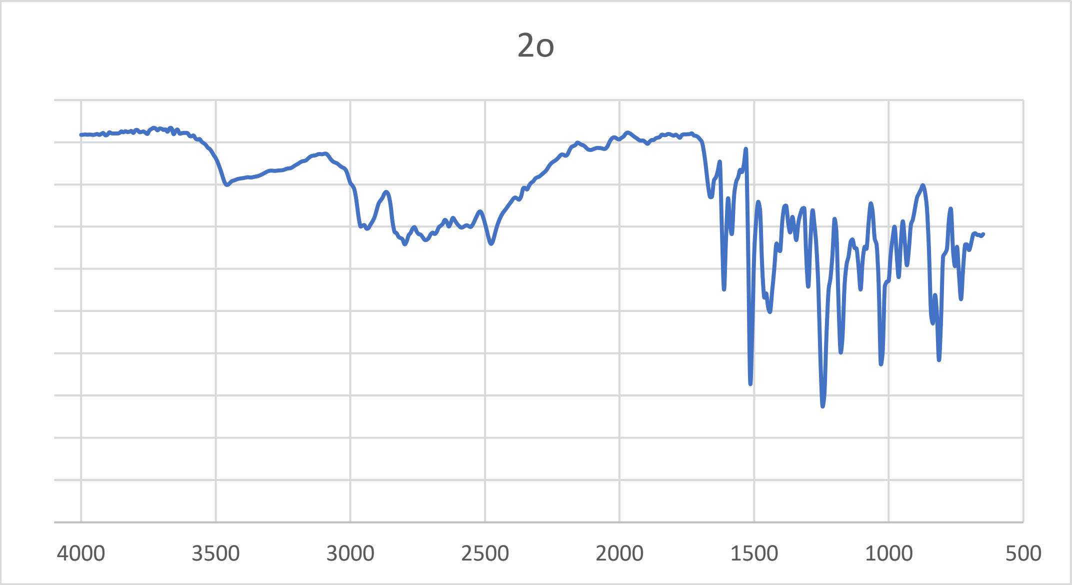

Figure S15. Infrared spectrum of 2o

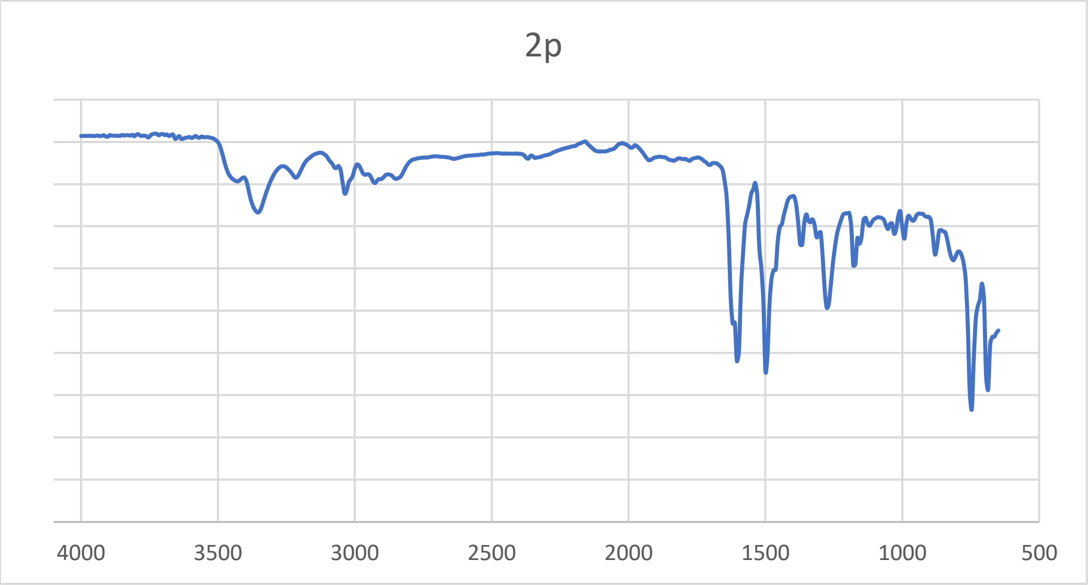

Figure S16. Infrared spectrum of 2p

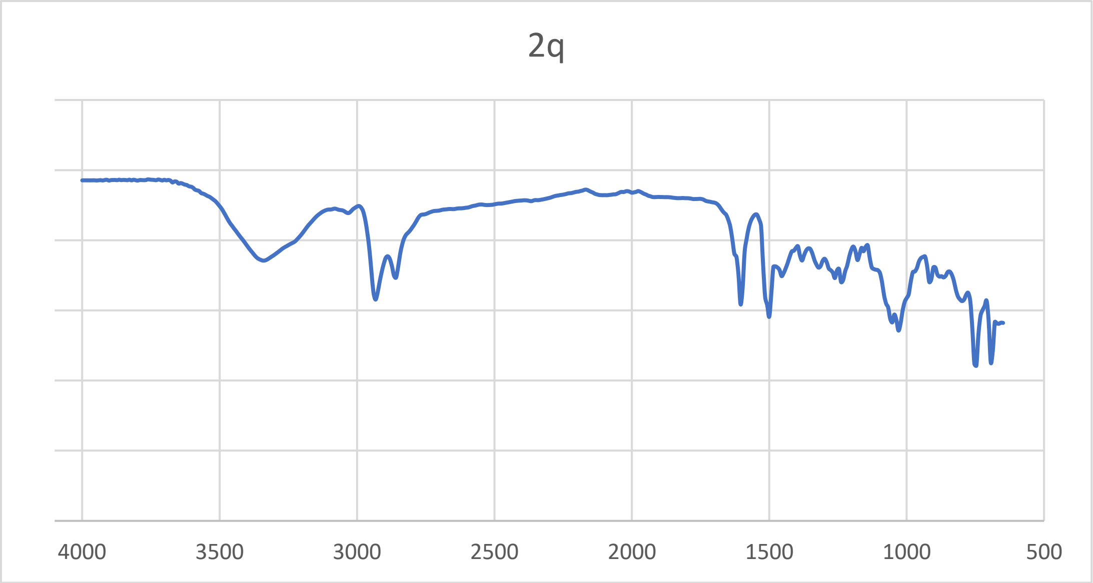

Figure S17. Infrared spectrum of 2q

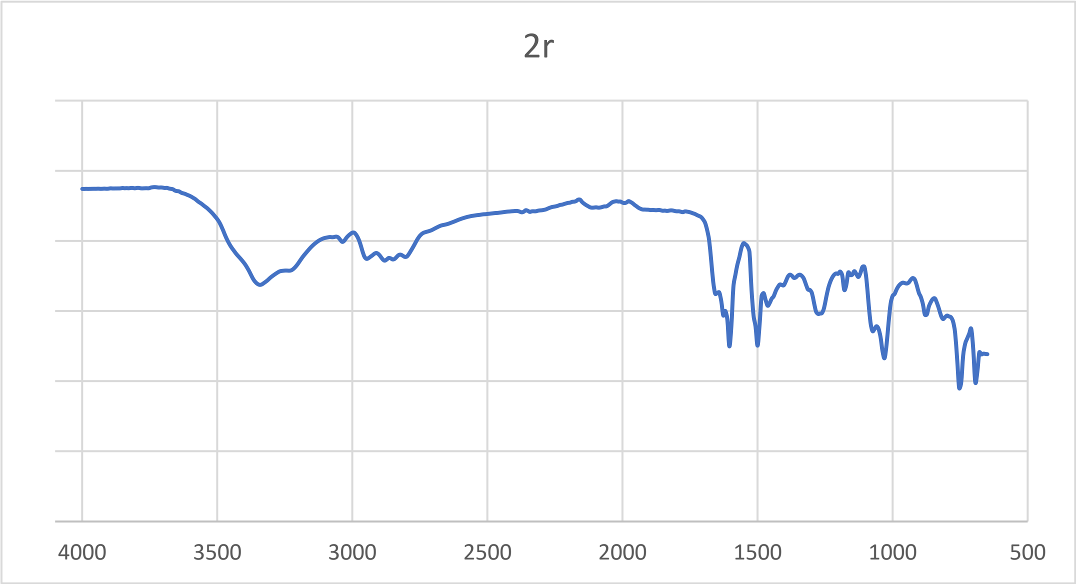

Figure S18. Infrared spectrum of 2r

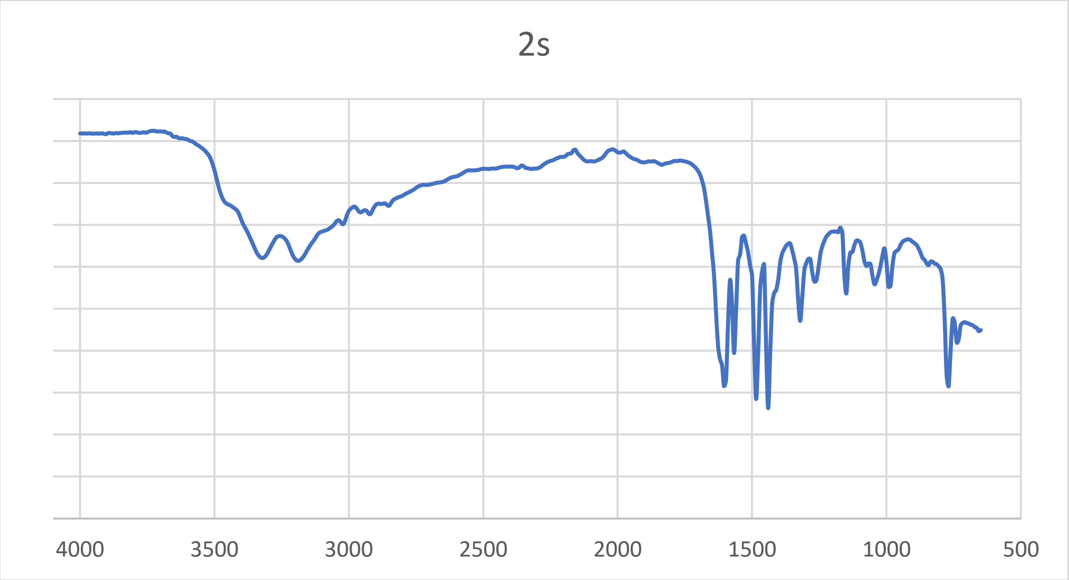

Figure S19. Infrared spectrum of 2s

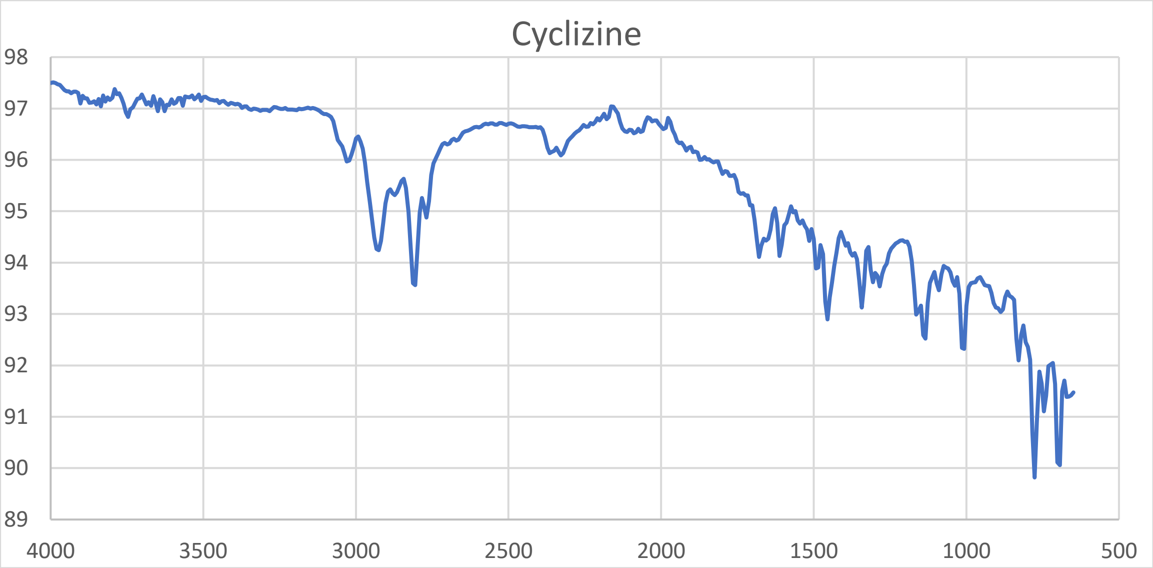

Figure S20. Infrared spectrum of cyclizine

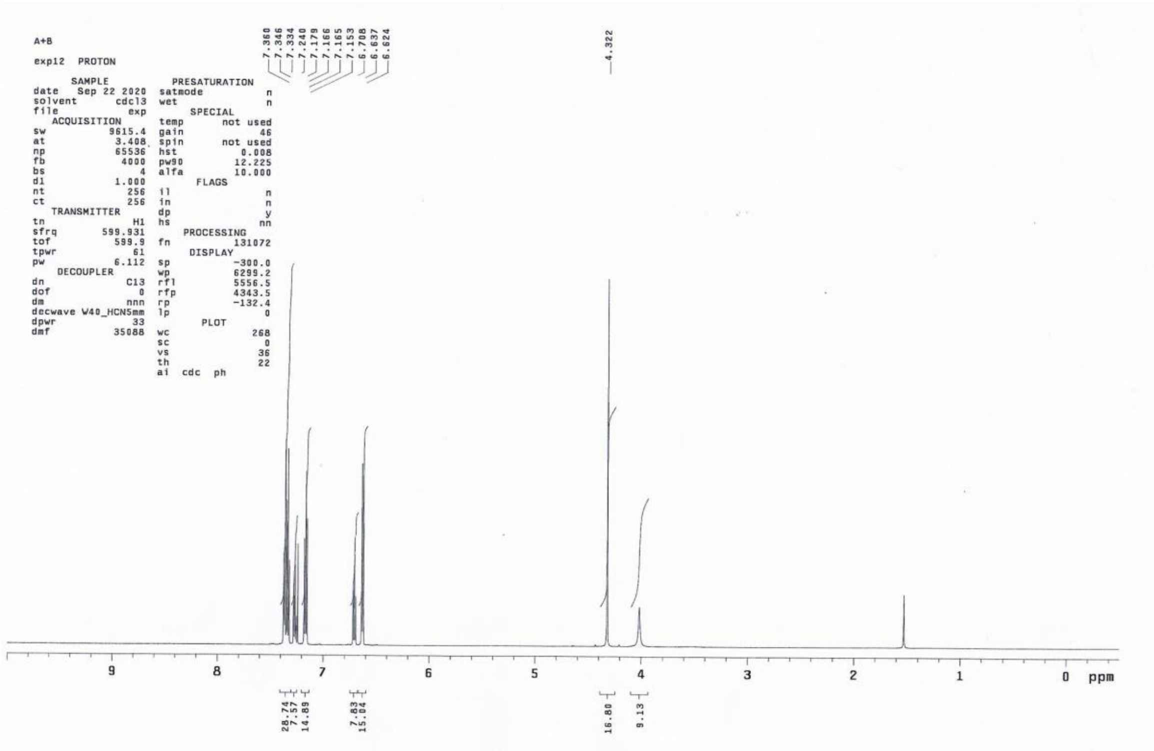

Figure S21. ^{1}H spectrum of 2a

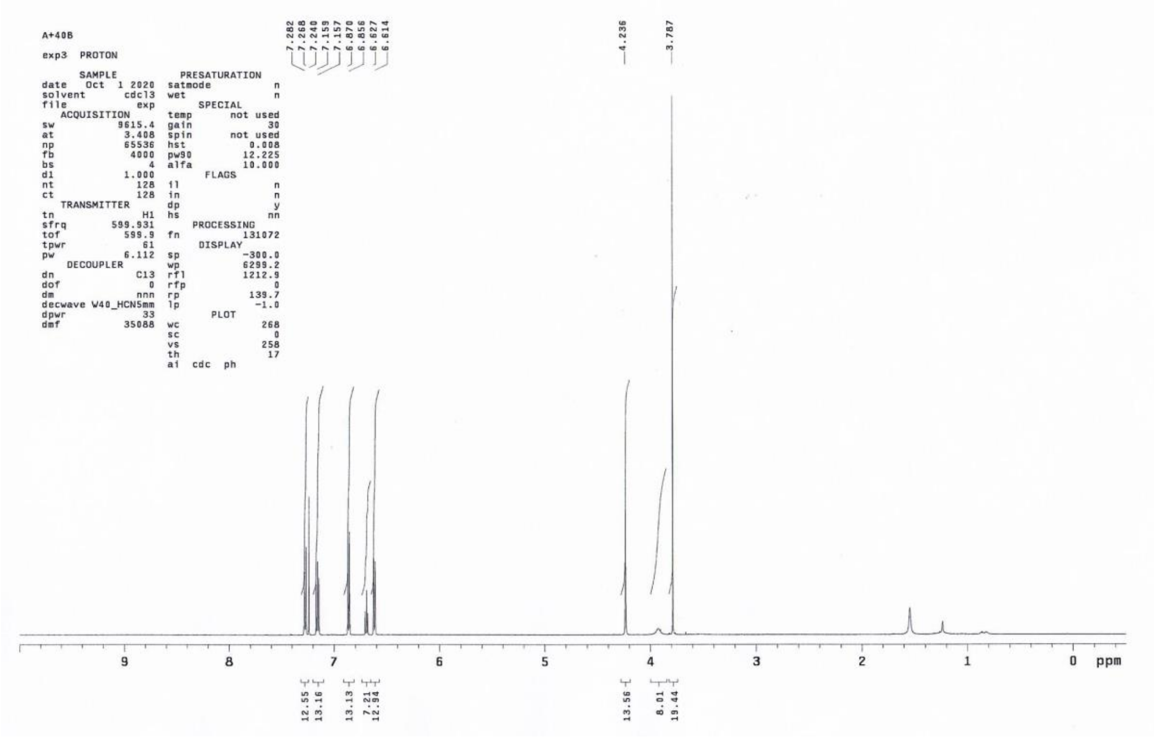

Figure S22. ^{1}H spectrum of 2b

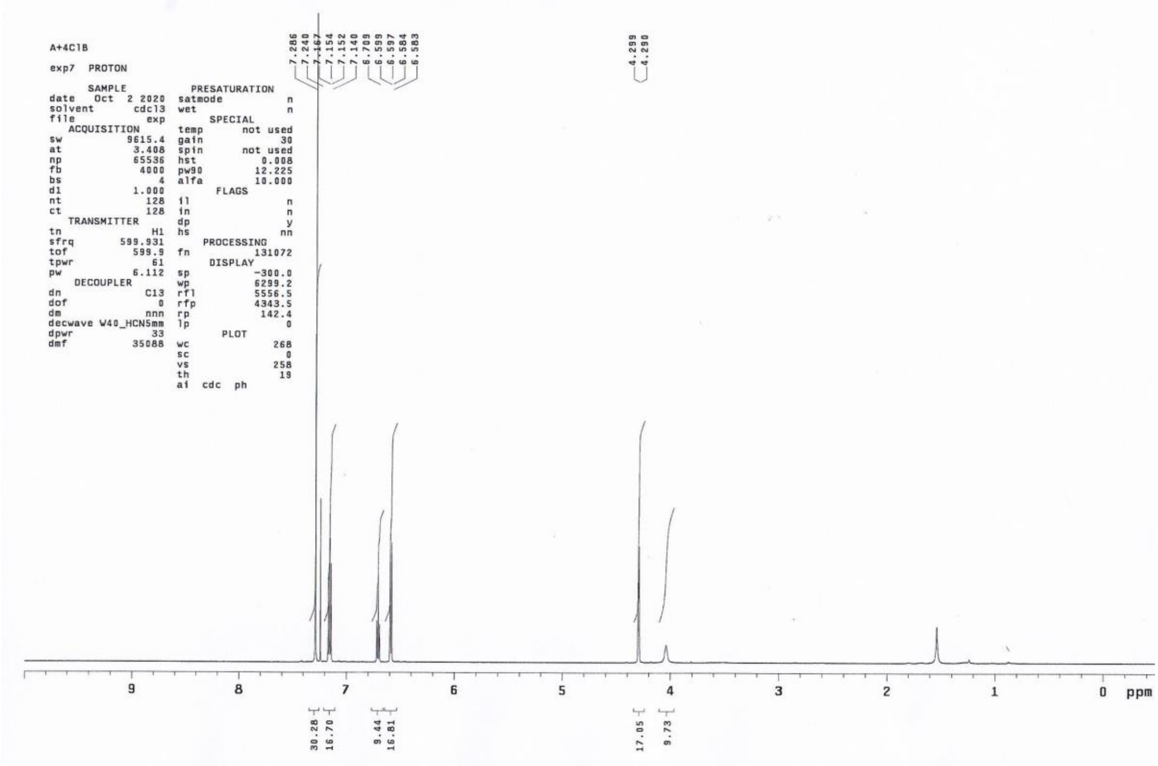

Figure S23. ^{1}H spectrum of 2c

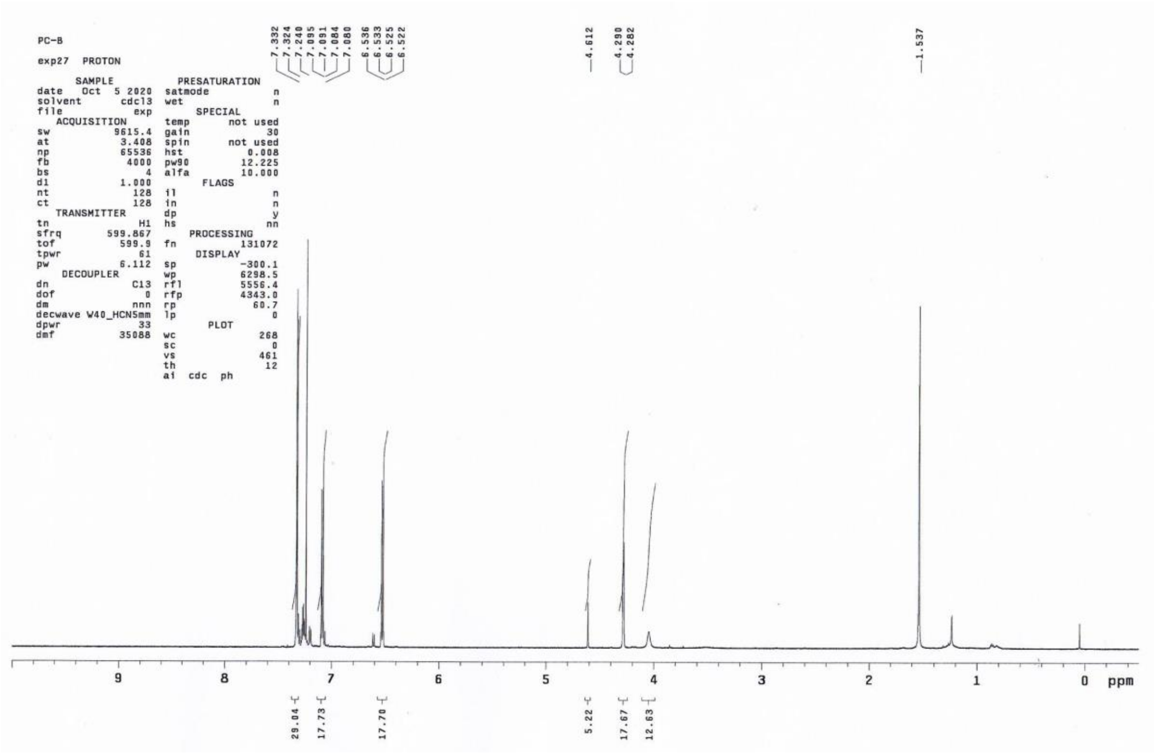

Figure S24. ^{1}H spectrum of 2d

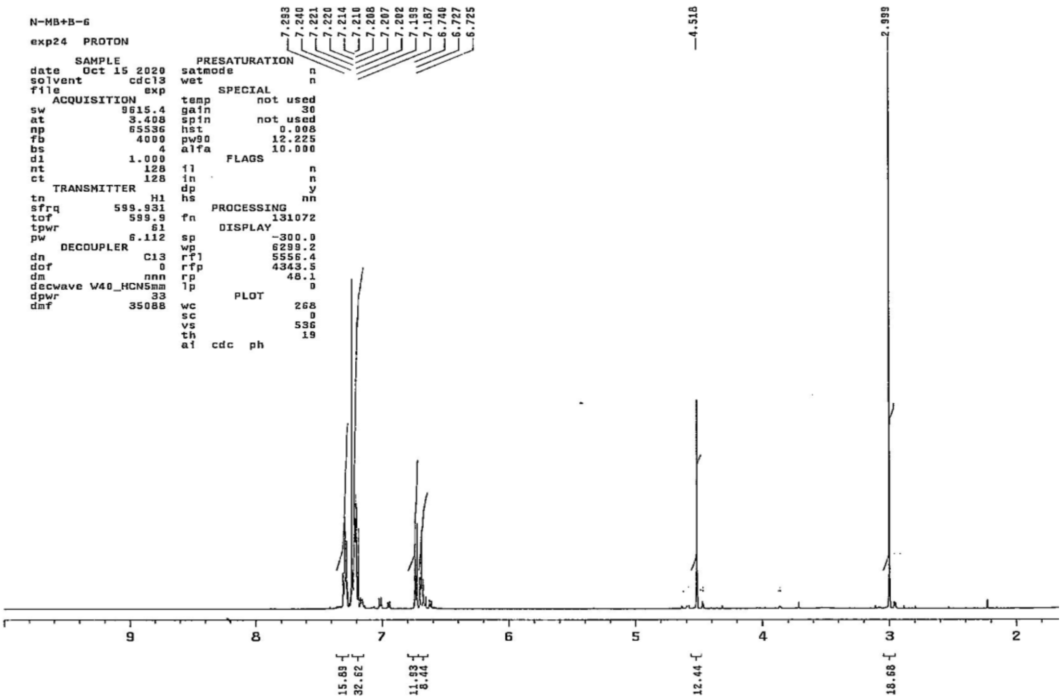

Figure S25. ^{1}H spectrum of 2e

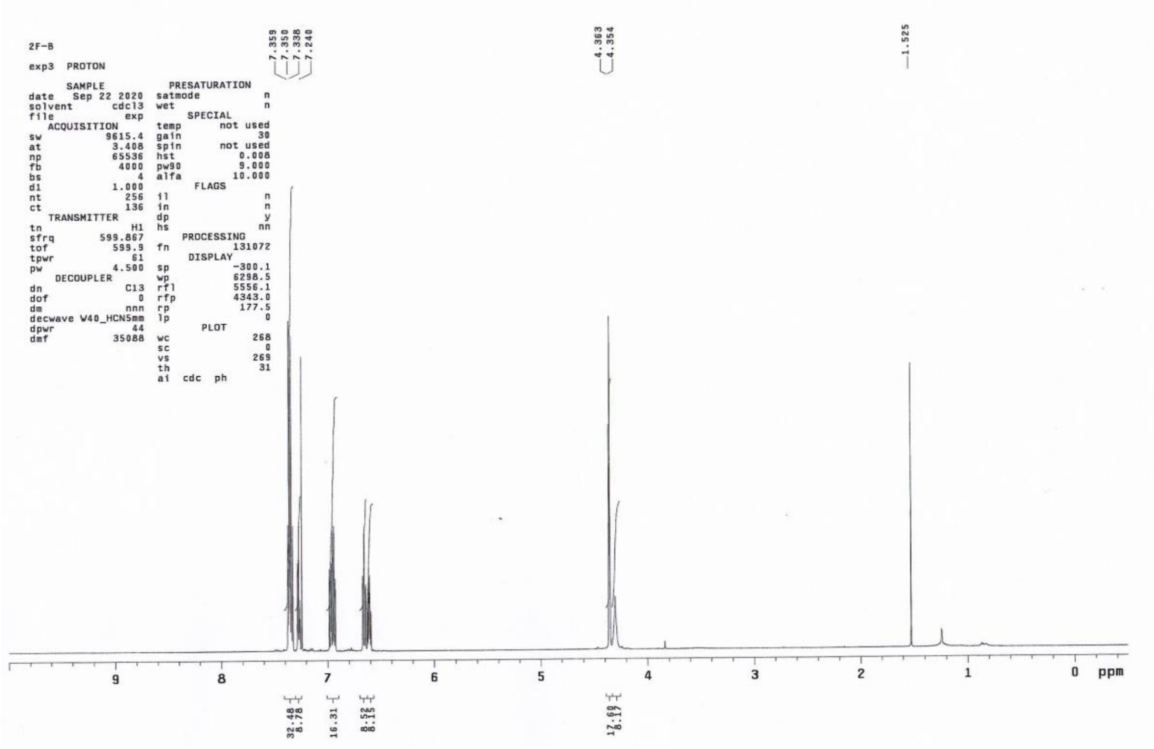

Figure S26. ^{1}H spectrum of 2f

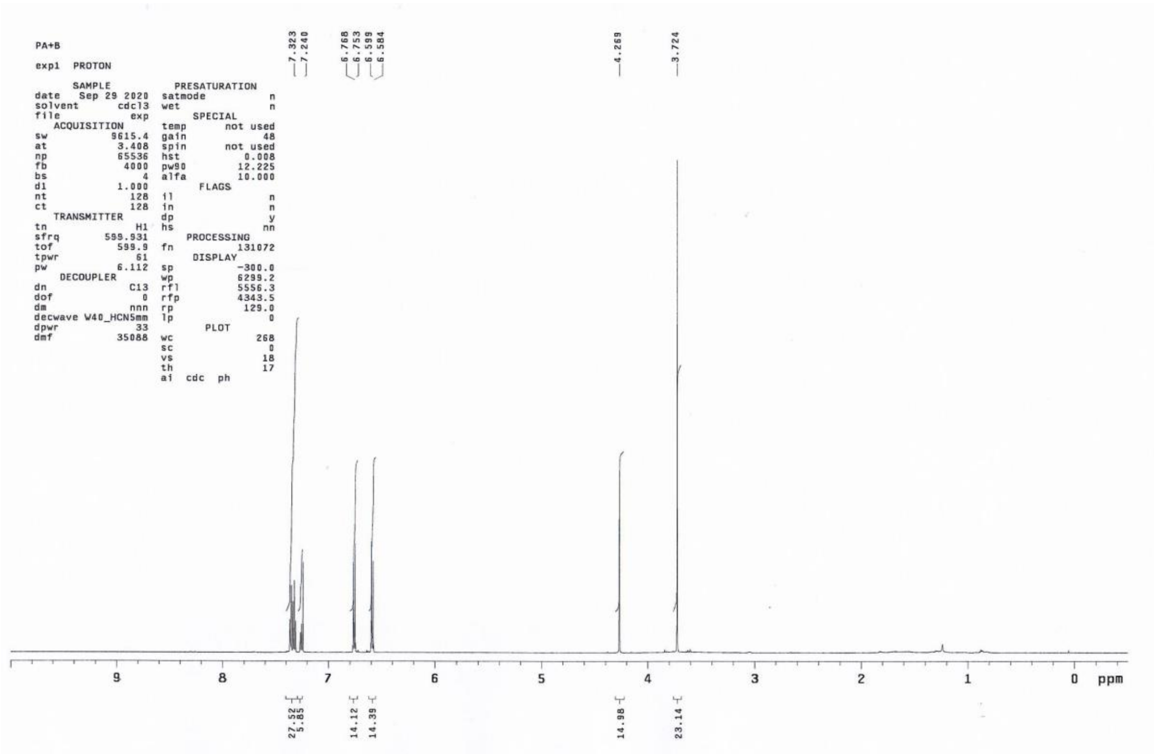

Figure S27. ^{1}H spectrum of 2g

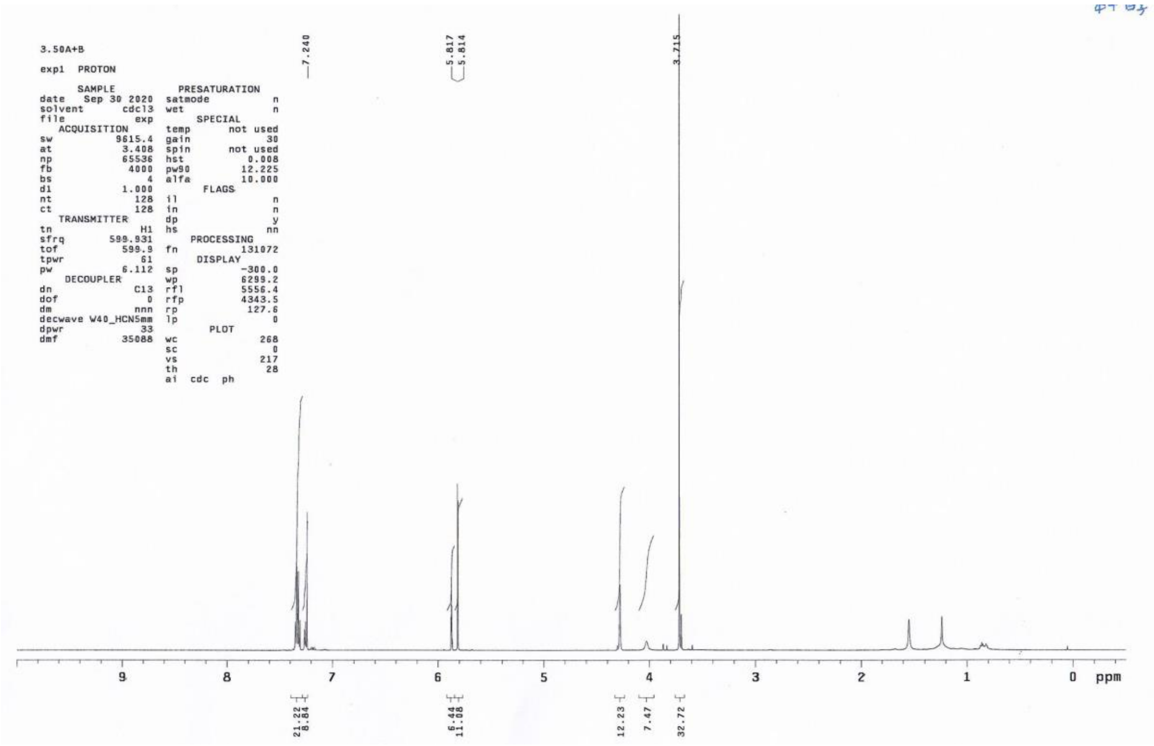

Figure S28. ^{1}H spectrum of 2h

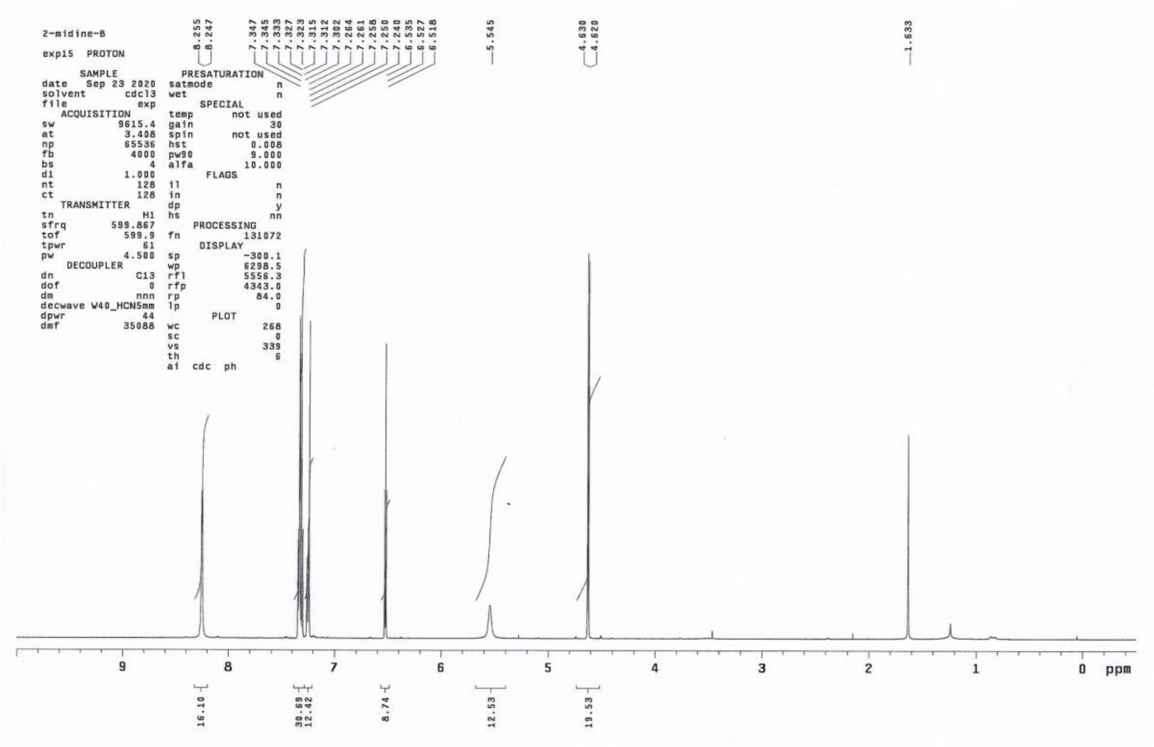

Figure S29. ^{1}H spectrum of 2i

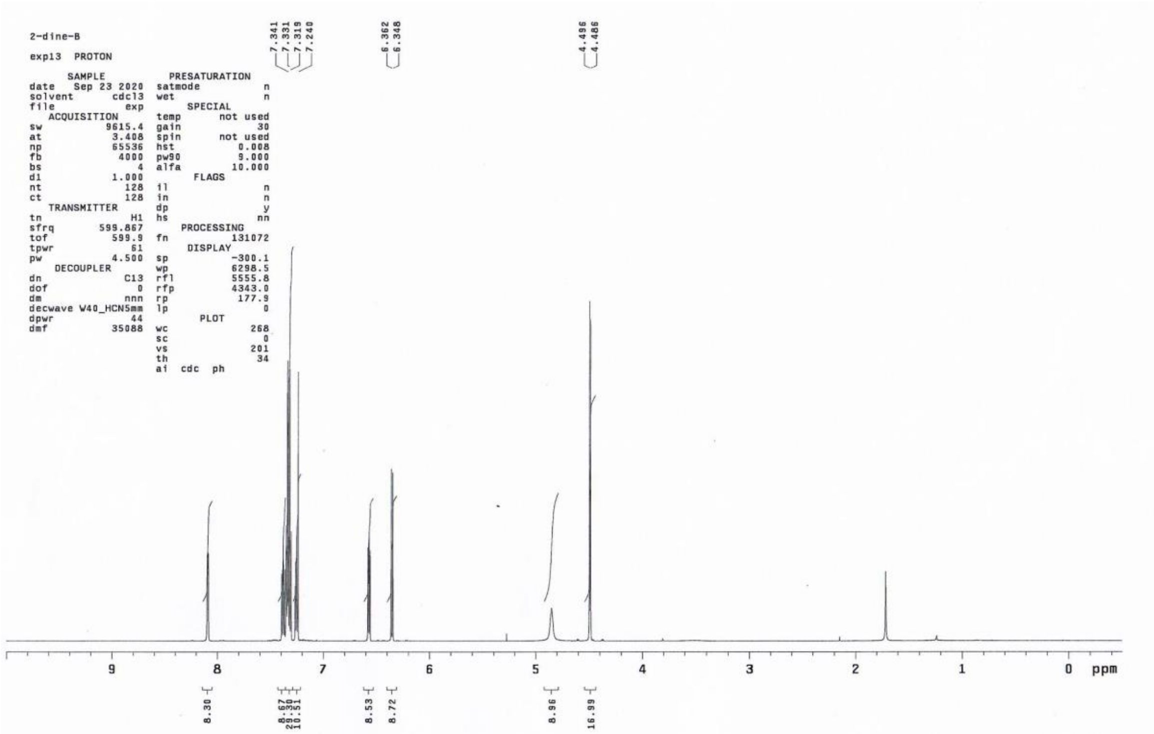

Figure S30. ^{1}H spectrum of 2j

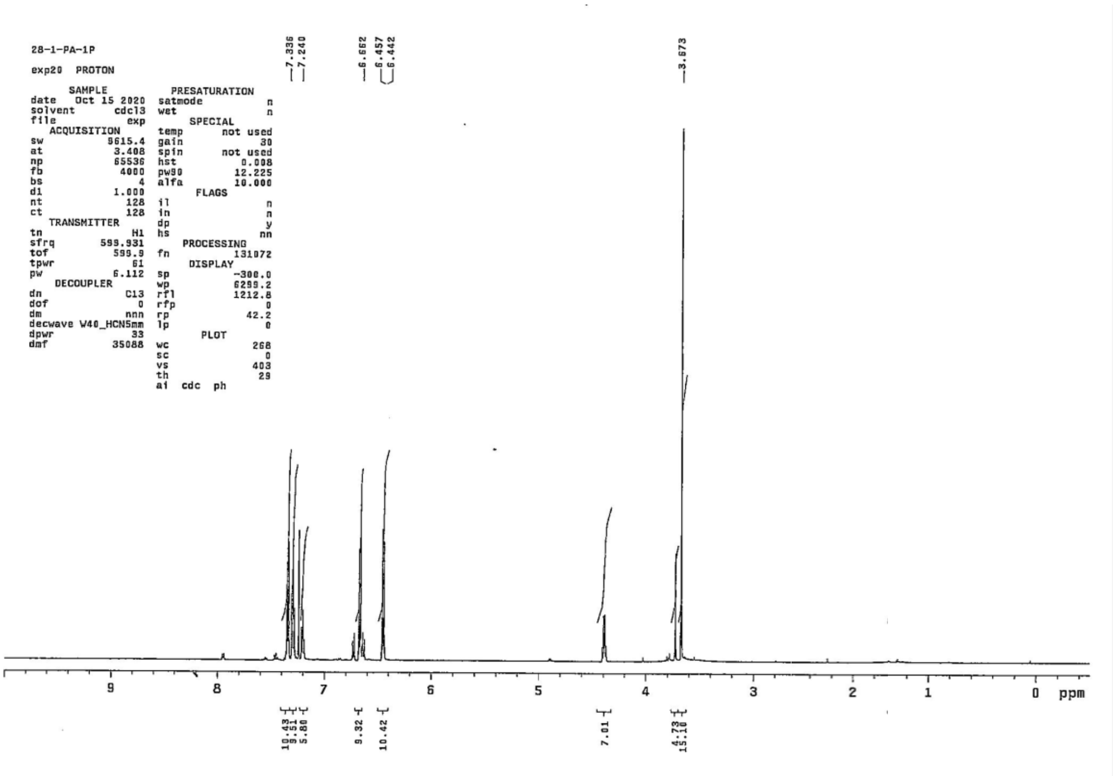

Figure S31. ^{1}H spectrum of 2k

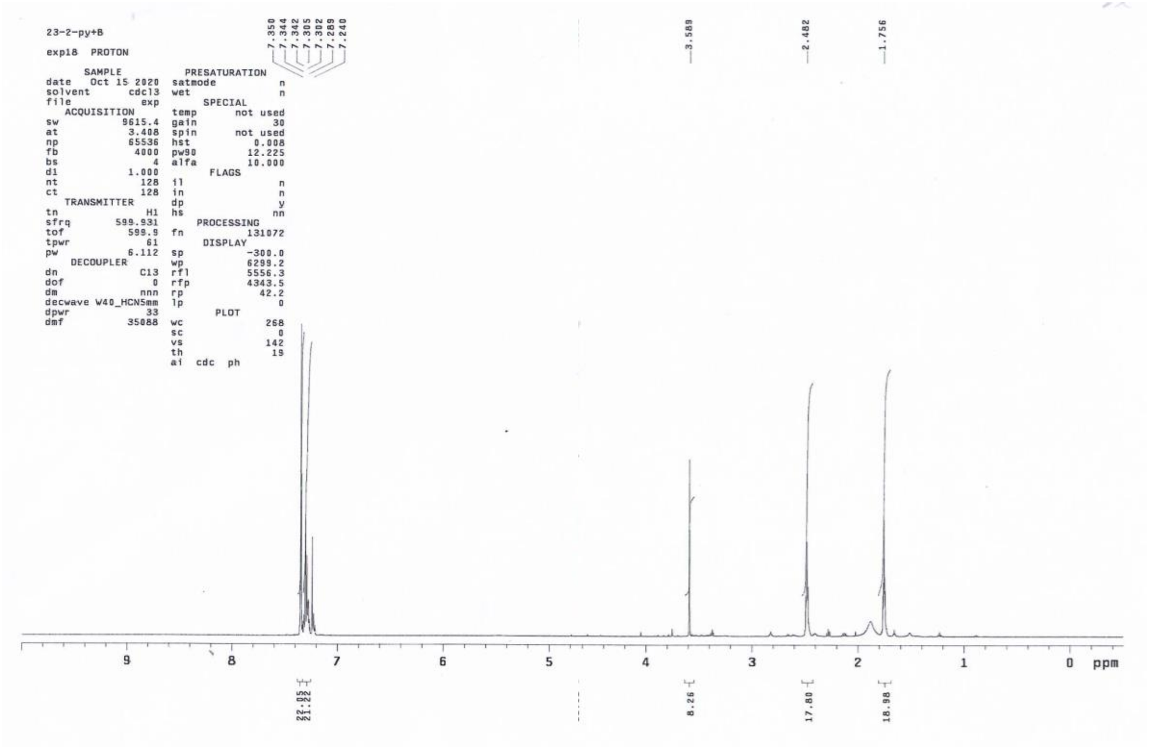

Figure S32. ^{1}H spectrum of 2l

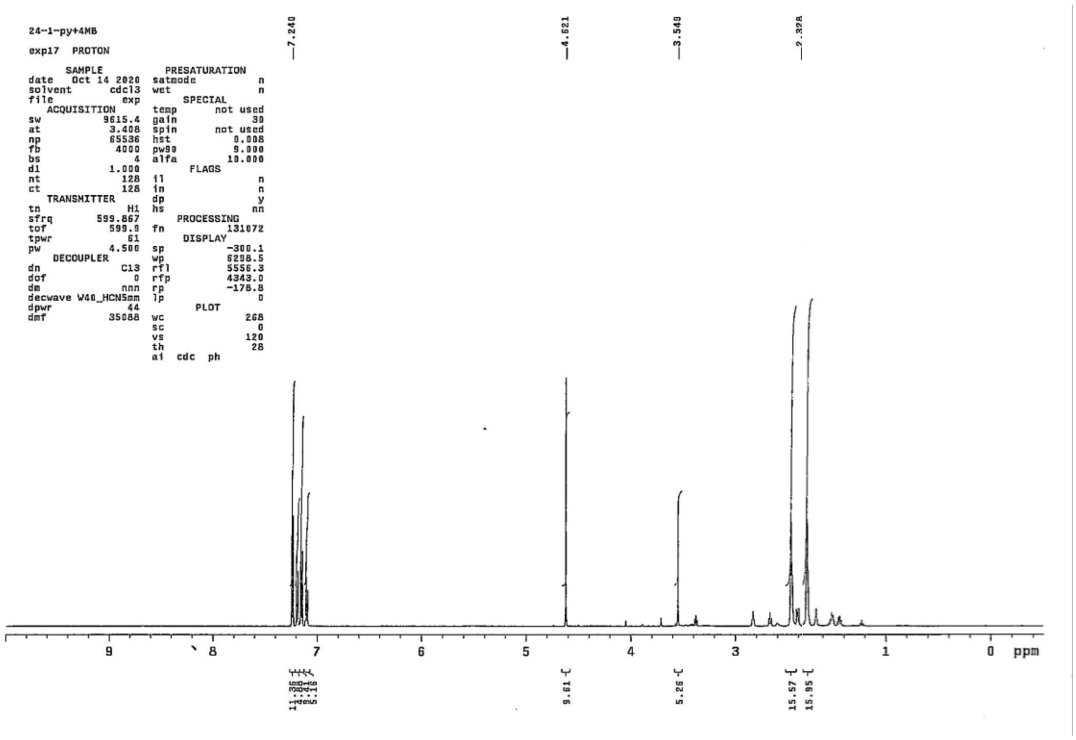

Figure S33. ^{1}H spectrum of 2m

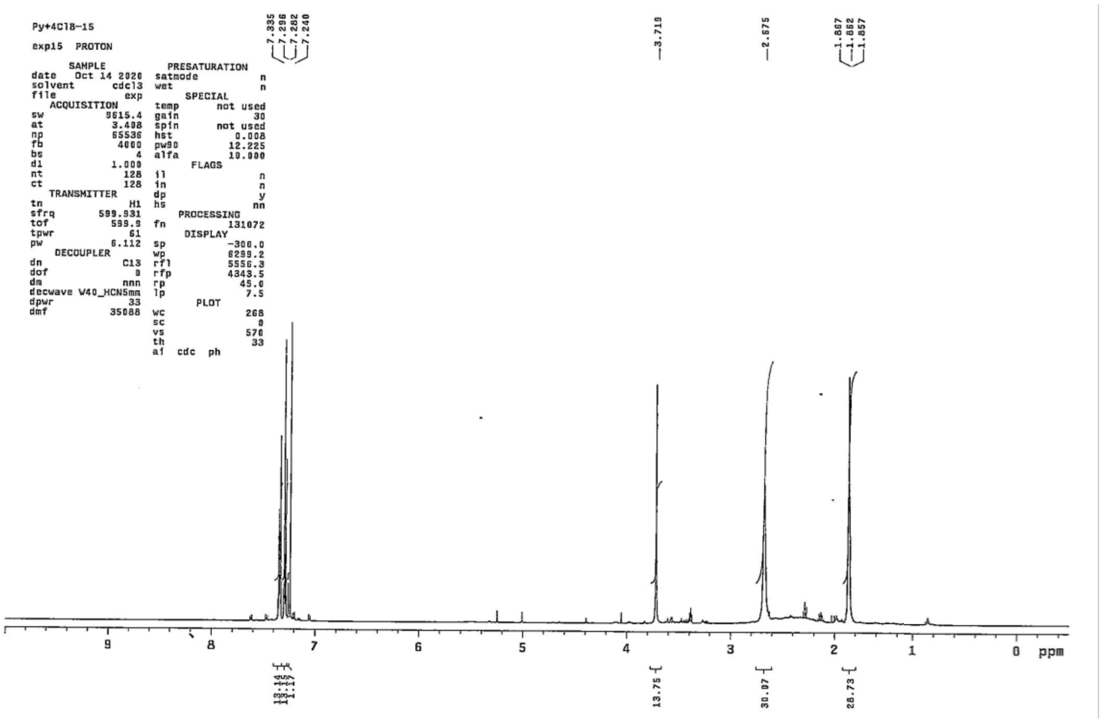

Figure S34. ^{1}H spectrum of 2n

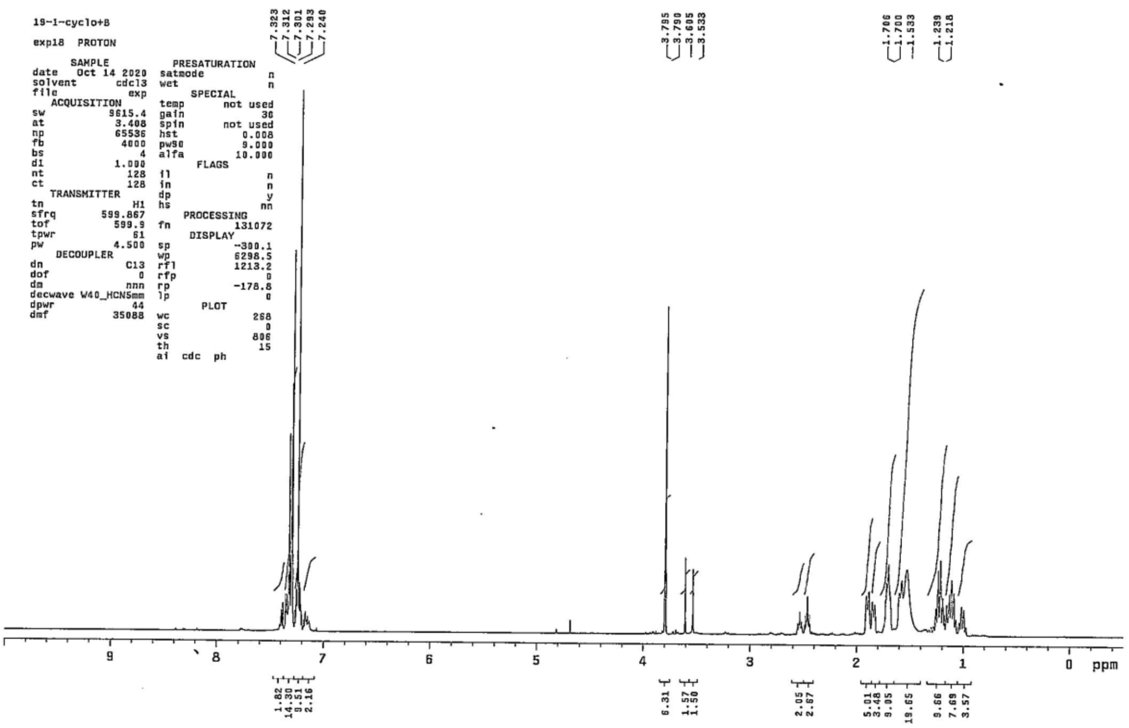

Figure S35. ^{1}H spectrum of 2o

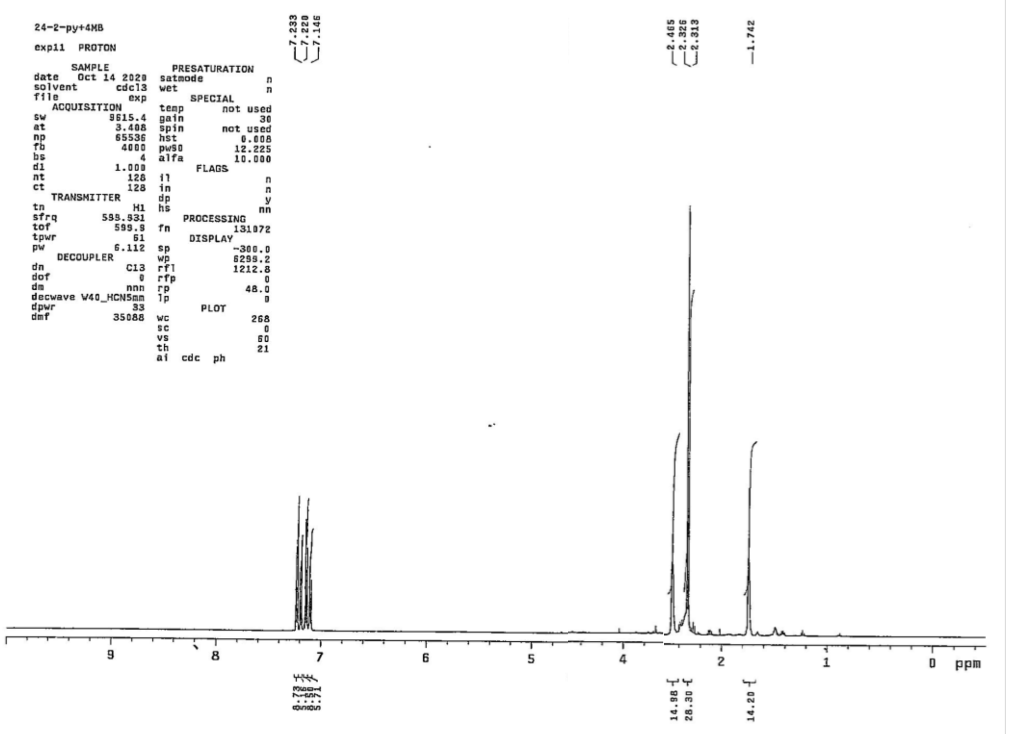

Figure S36. ^{1}H spectrum of 2p

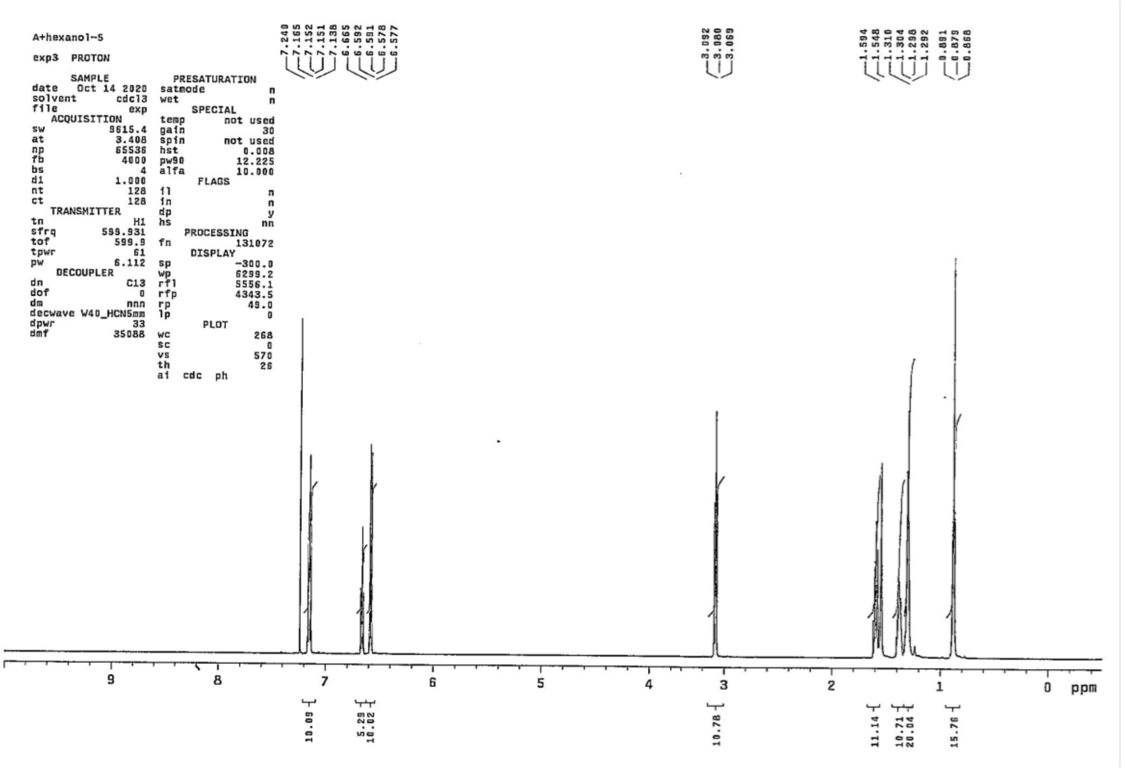

Figure S37. ^{1}H spectrum of 2q

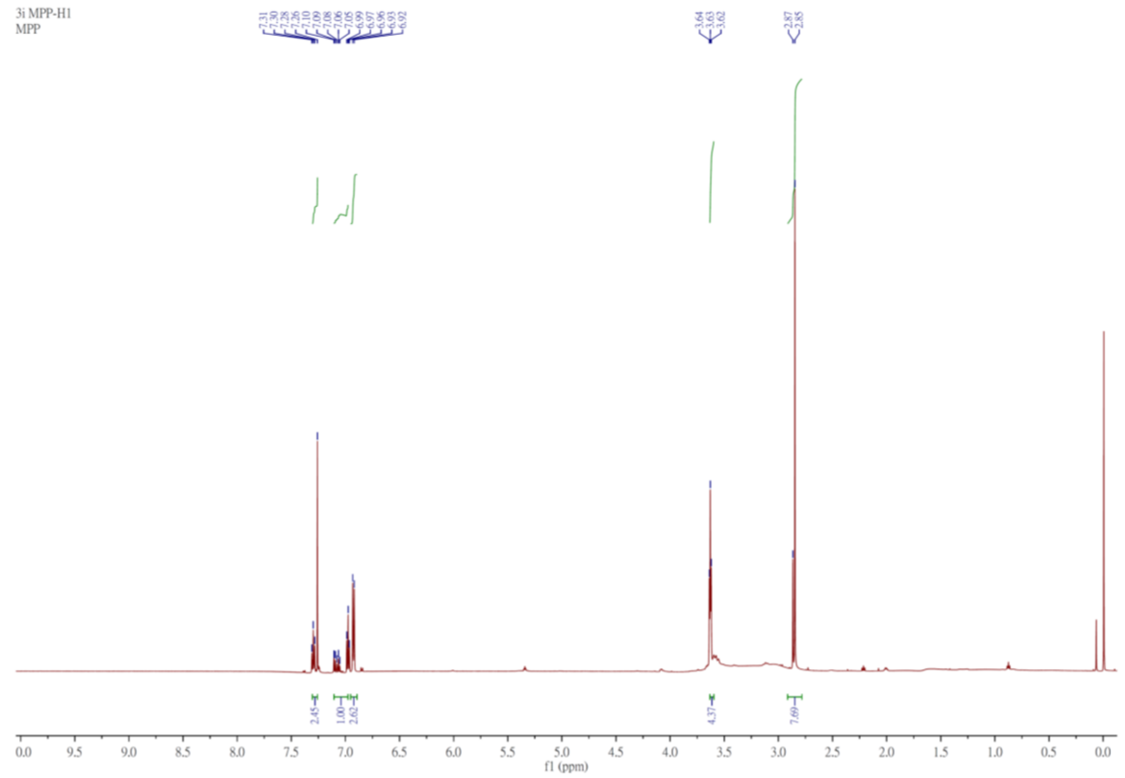

Figure S38. ^{1}H spectrum of 2r

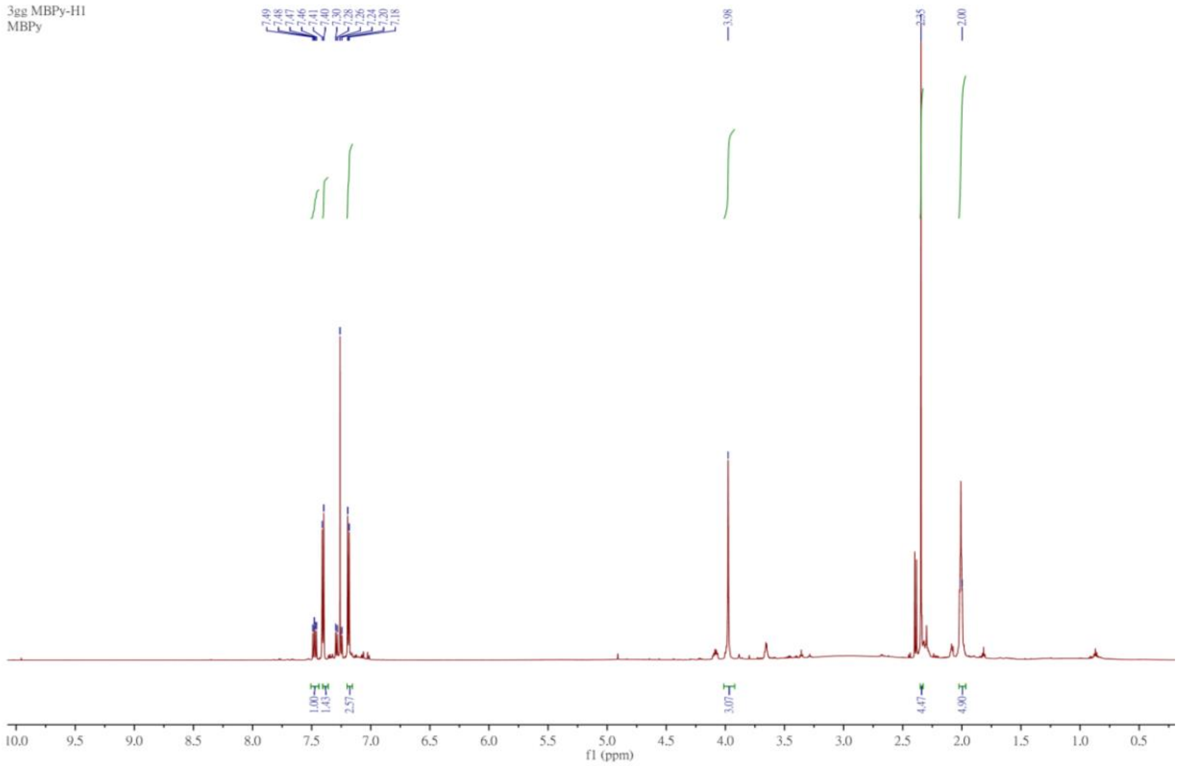

Figure S39. ^{1}H spectrum of 2s

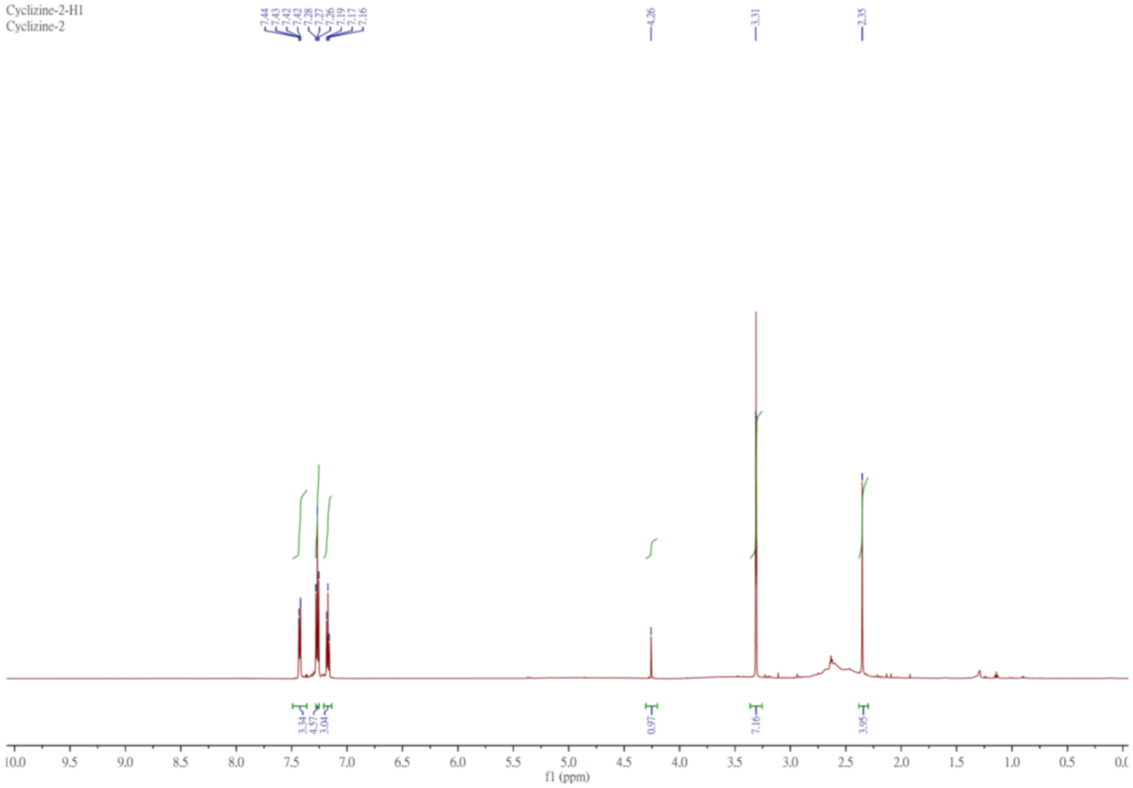

Figure S40. ^{1}H spectrum of cyclizine

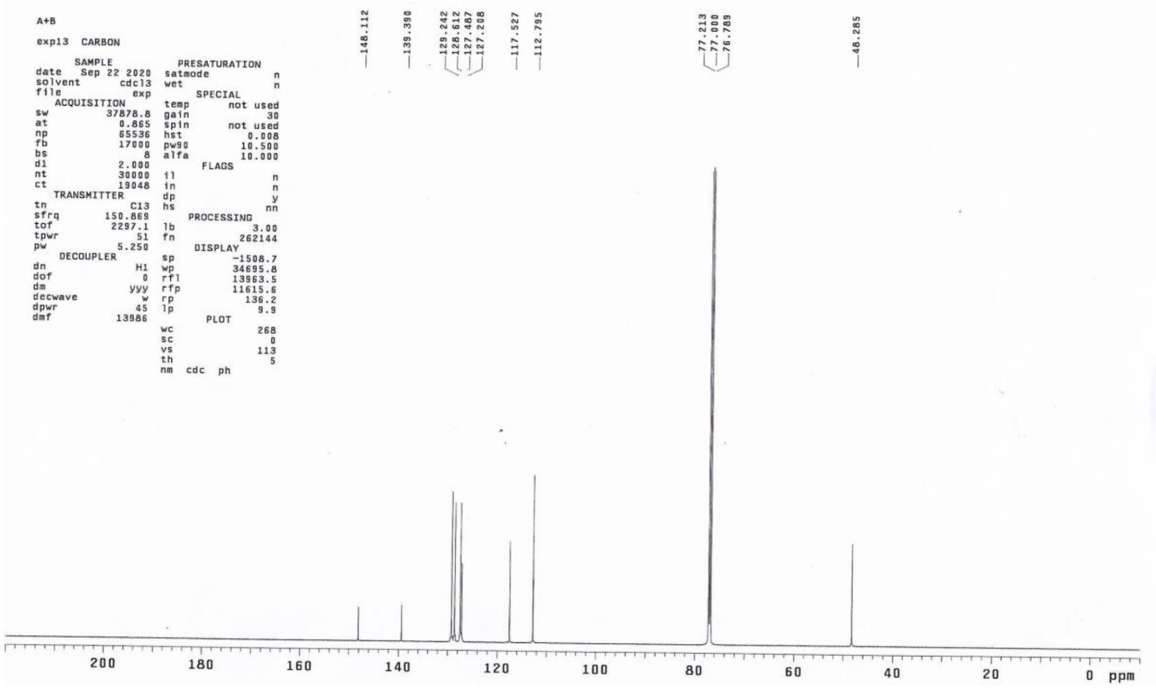

Figure S41. ^{13}C spectrum of 2a

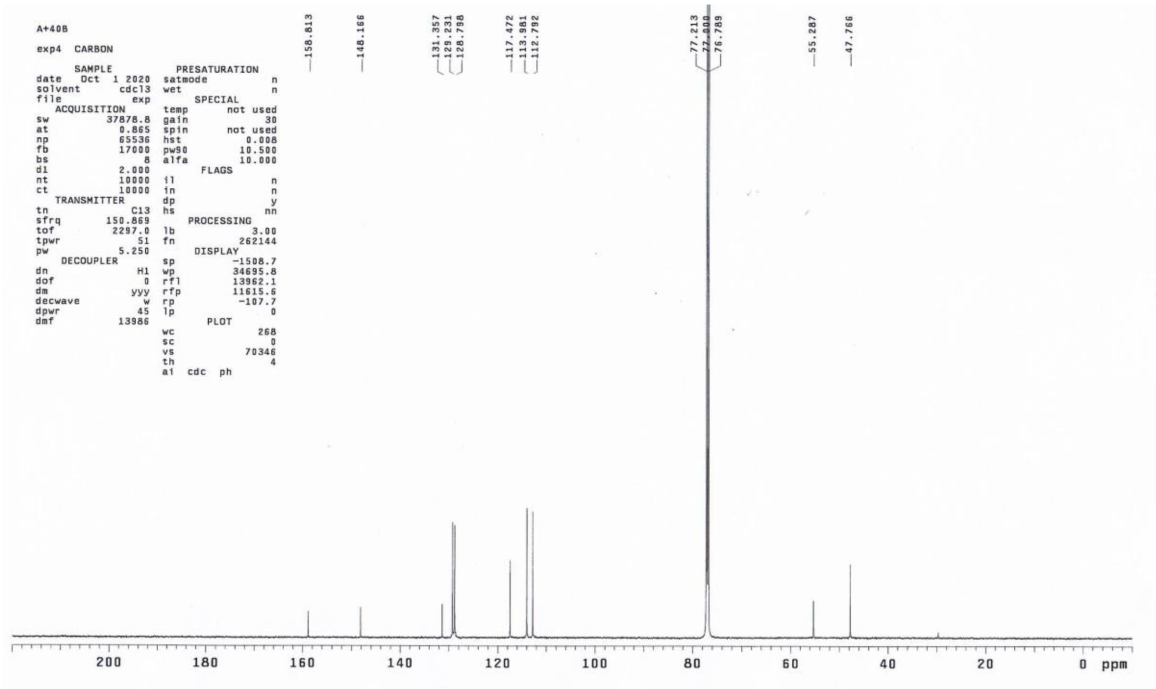

Figure S42. ^{13}C spectrum of 2b

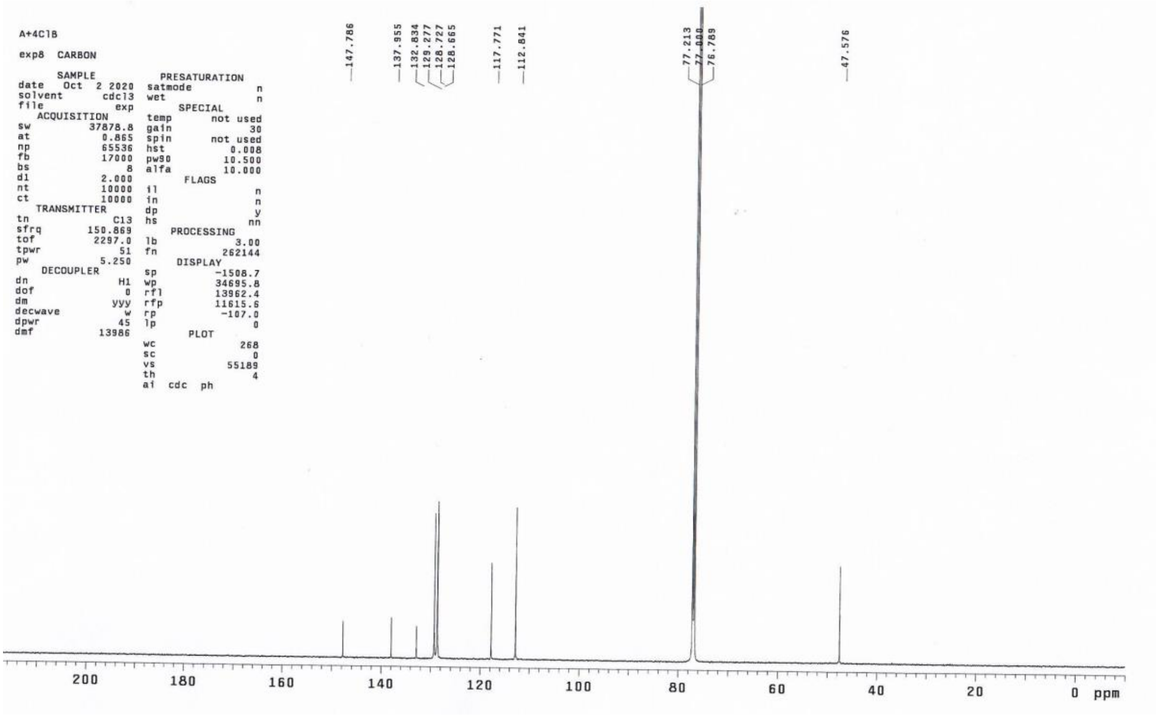

Figure S43. ^{13}C spectrum of 2c

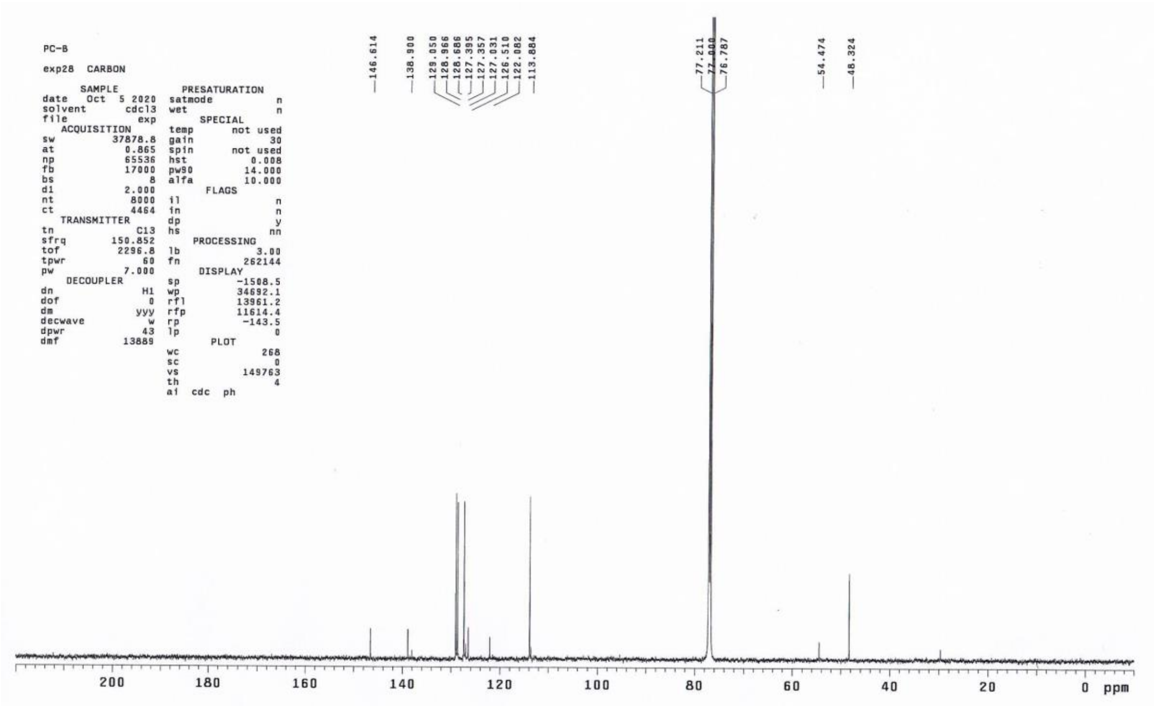

Figure S44. ^{13}C spectrum of 2d

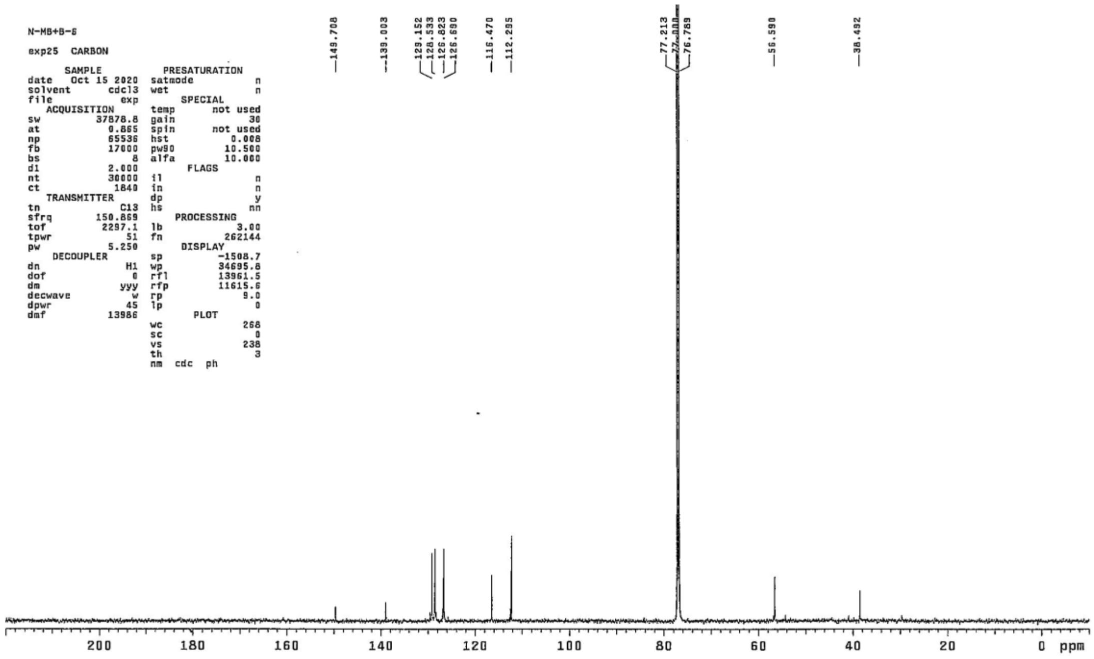

Figure S45. ^{13}C spectrum of 2e

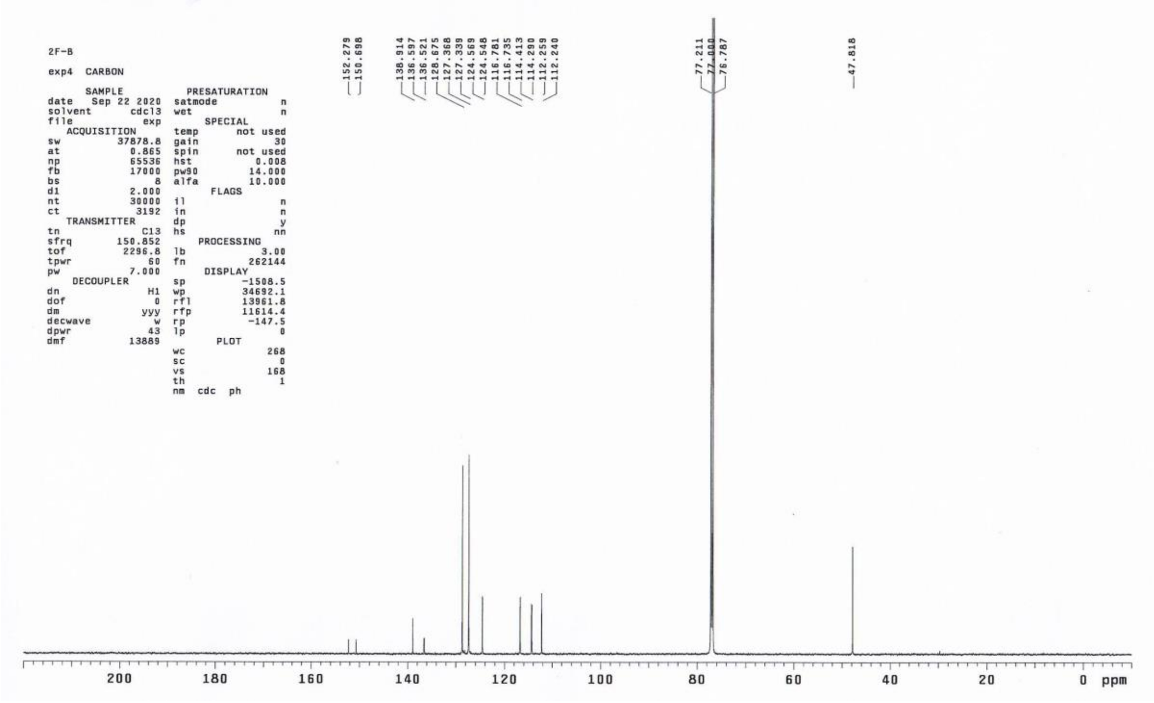

Figure S46. ^{13}C spectrum of 2f

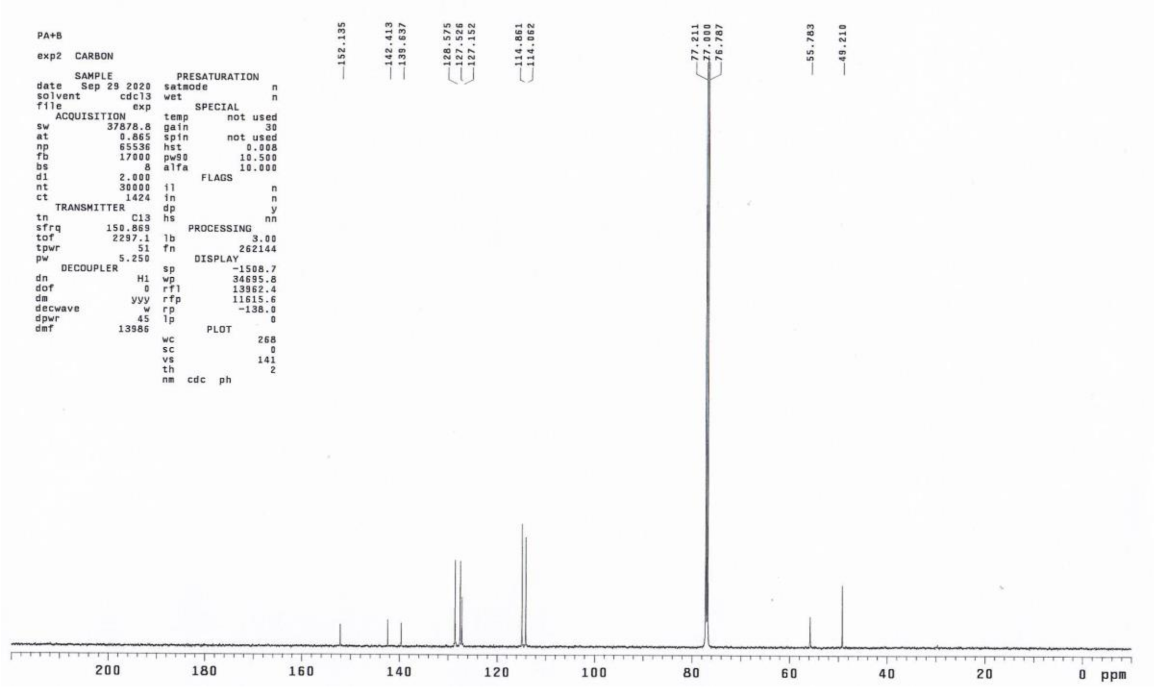

Figure S47. ^{13}C spectrum of 2g

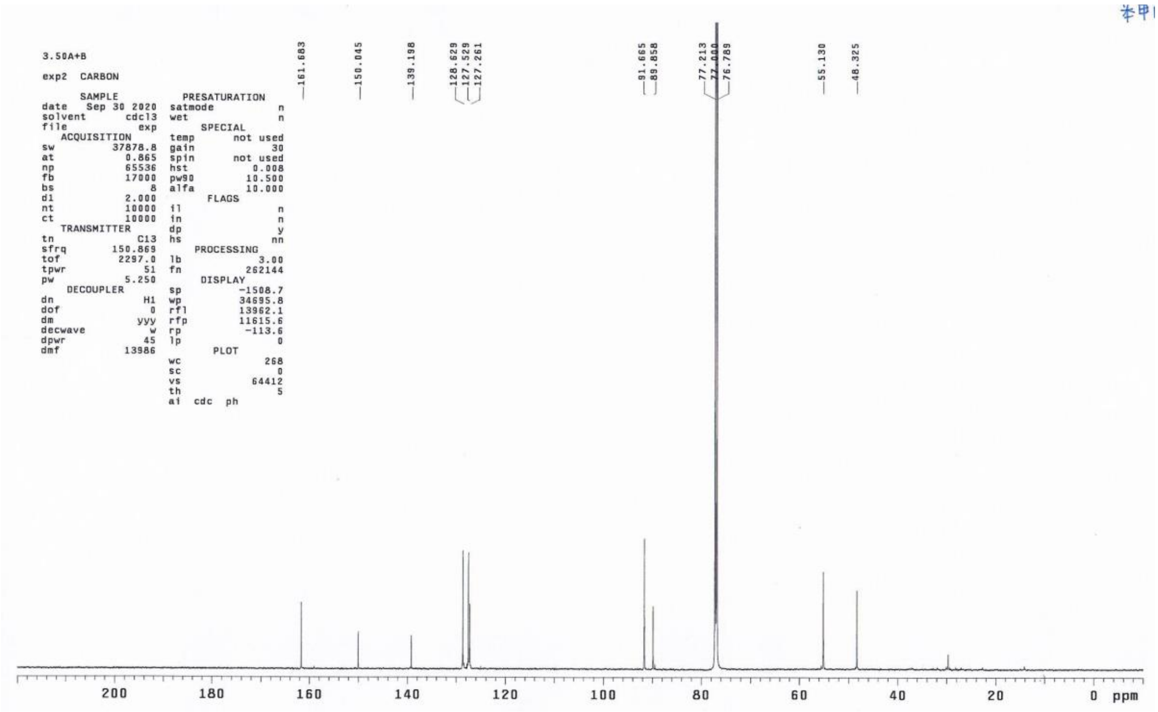

Figure S48. ^{13}C spectrum of 2h

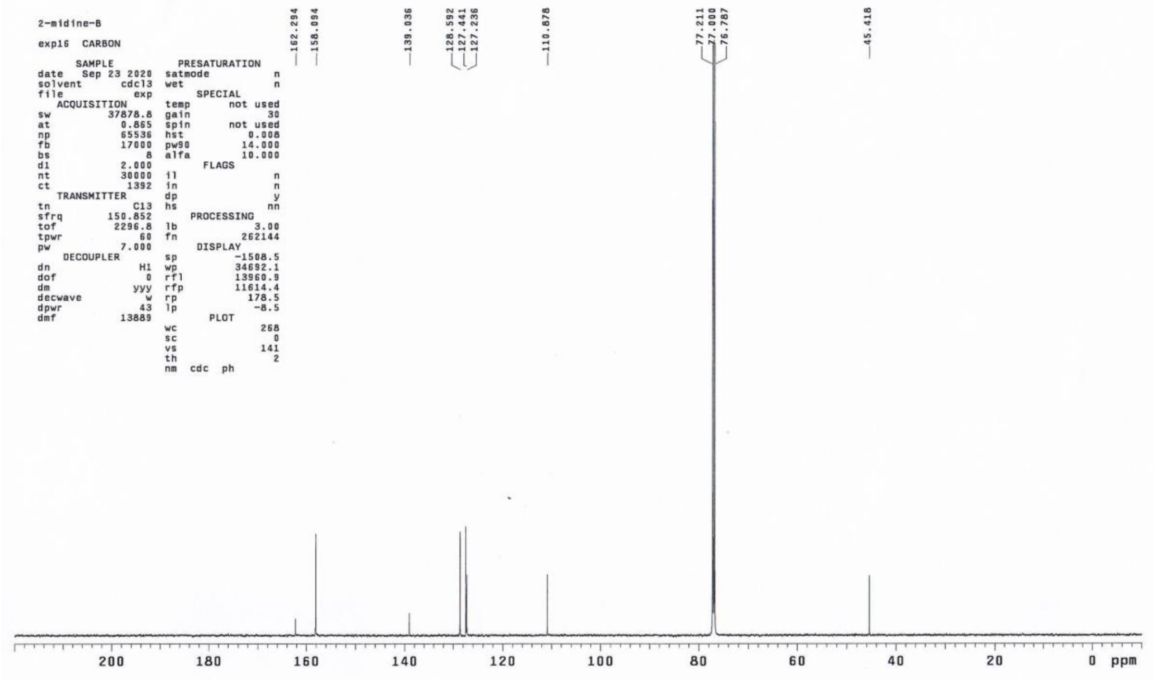

Figure S49. ^{13}C spectrum of 2i

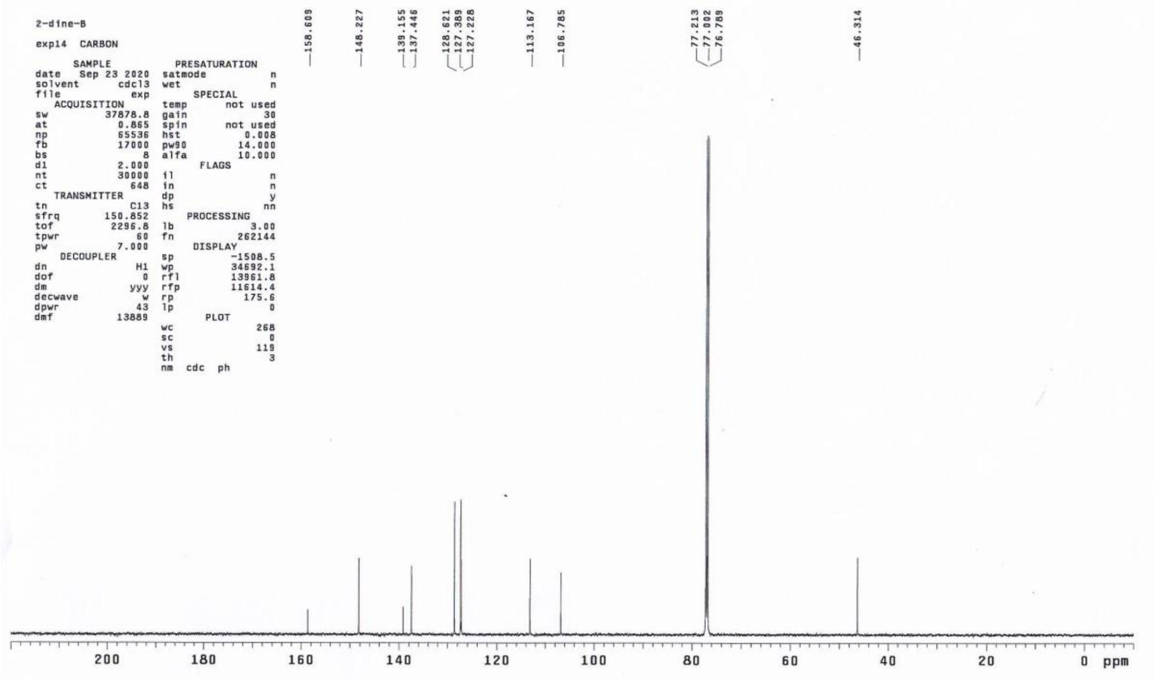

Figure S50. ^{13}C spectrum of 2j

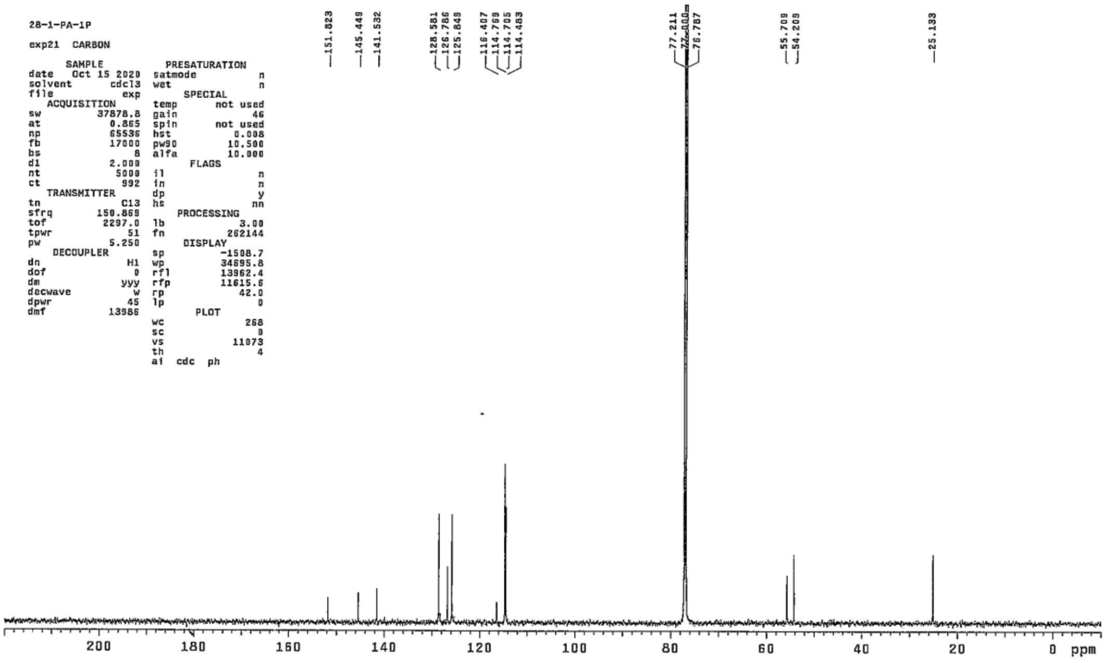

Figure S51. ^{13}C spectrum of 2k

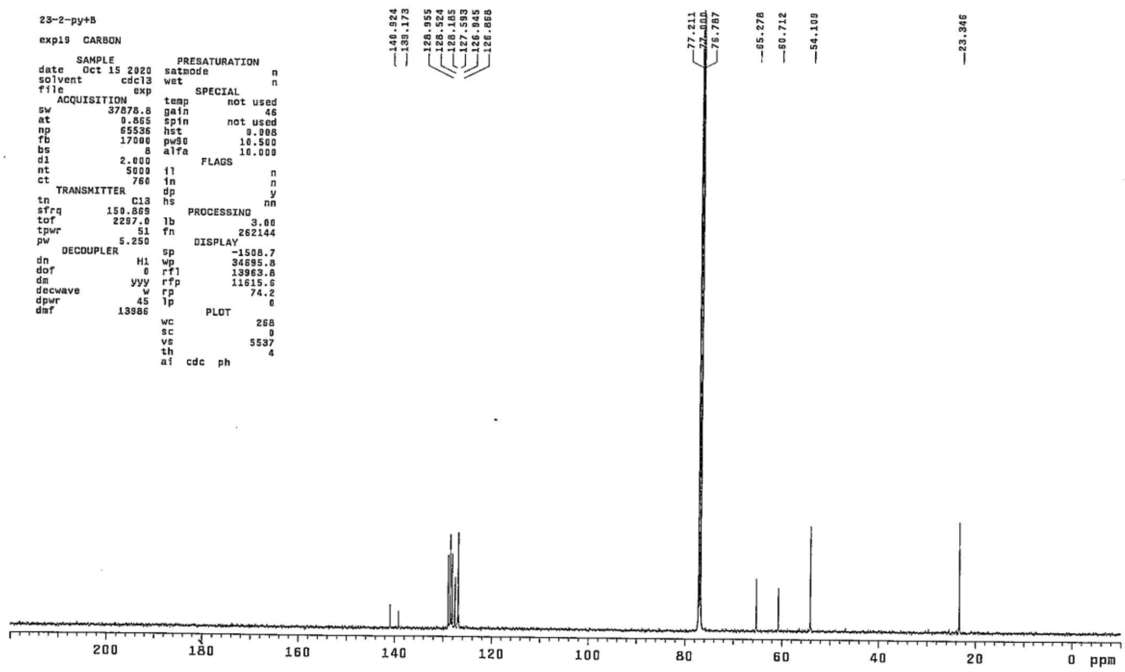

Figure S52. ^{13}C spectrum of 2l

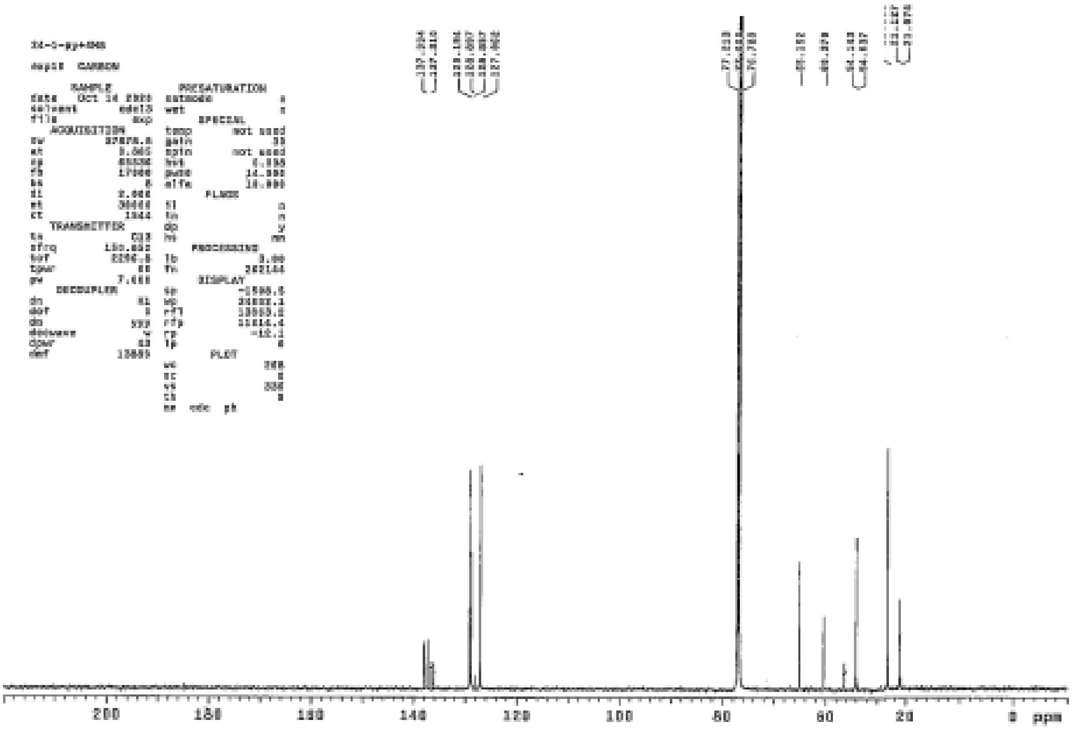

Figure S53. ^{13}C spectrum of 2m

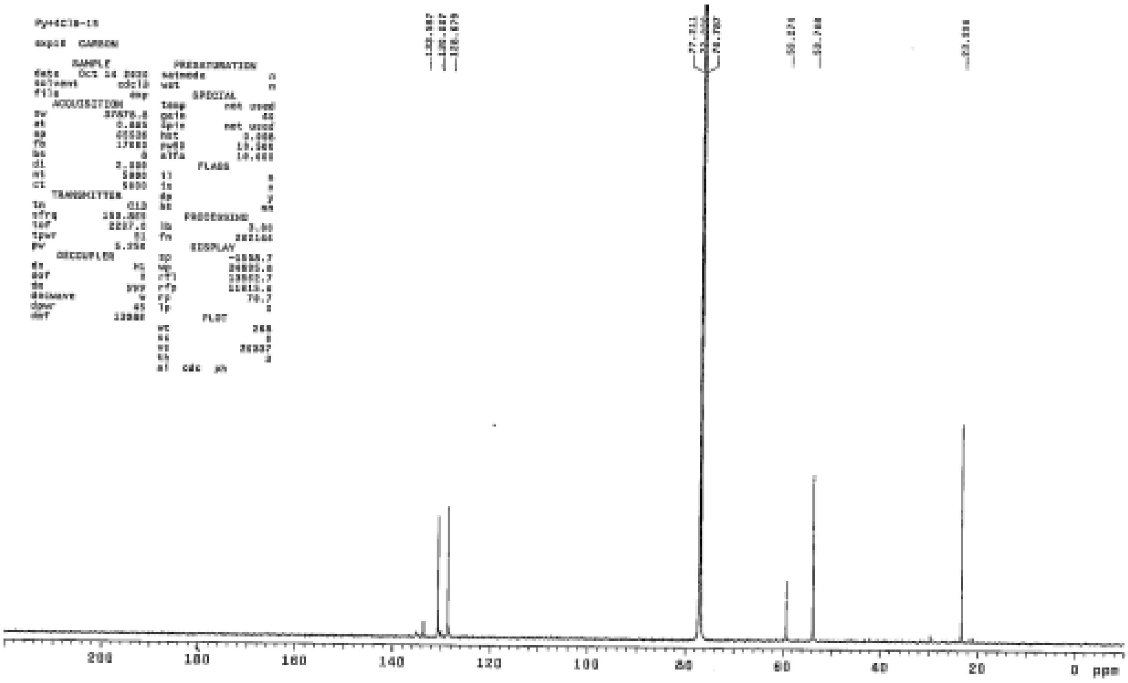

Figure S54. ^{13}C spectrum of 2n

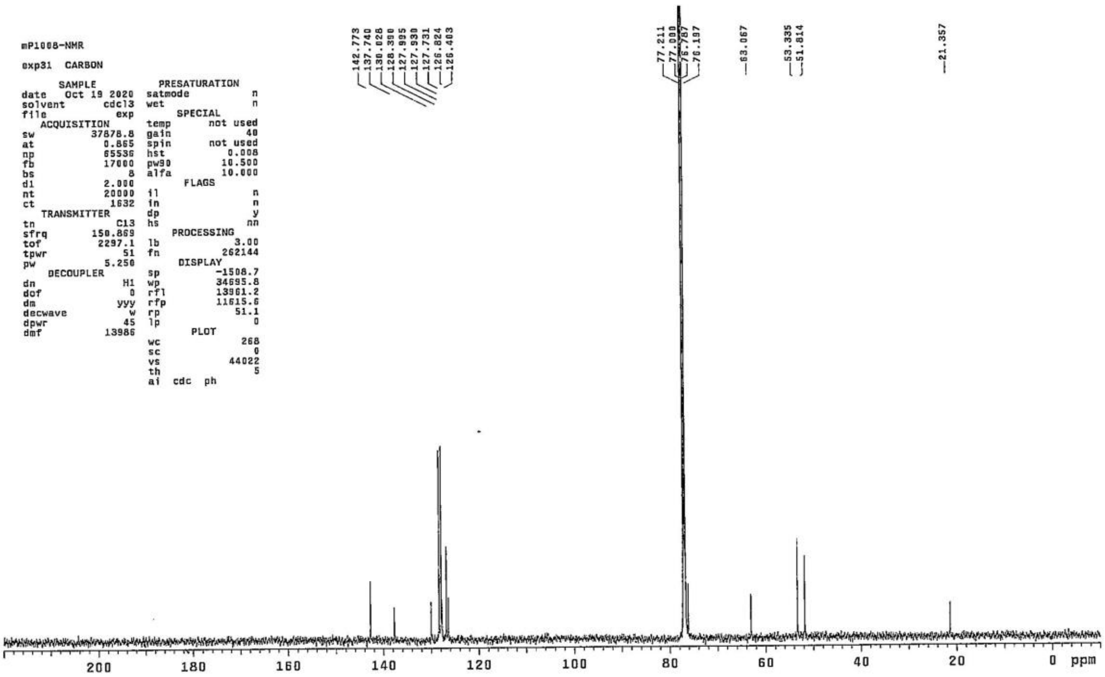

Figure S55. ^{13}C spectrum of 2o

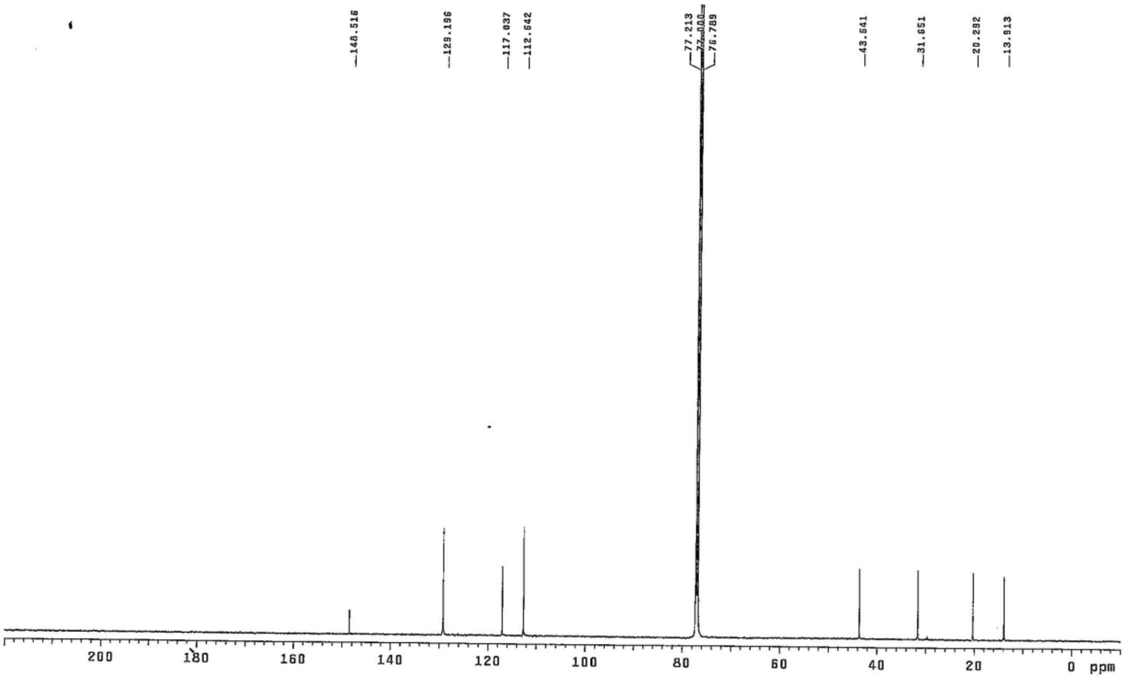

Figure S56. ^{13}C spectrum of 2p

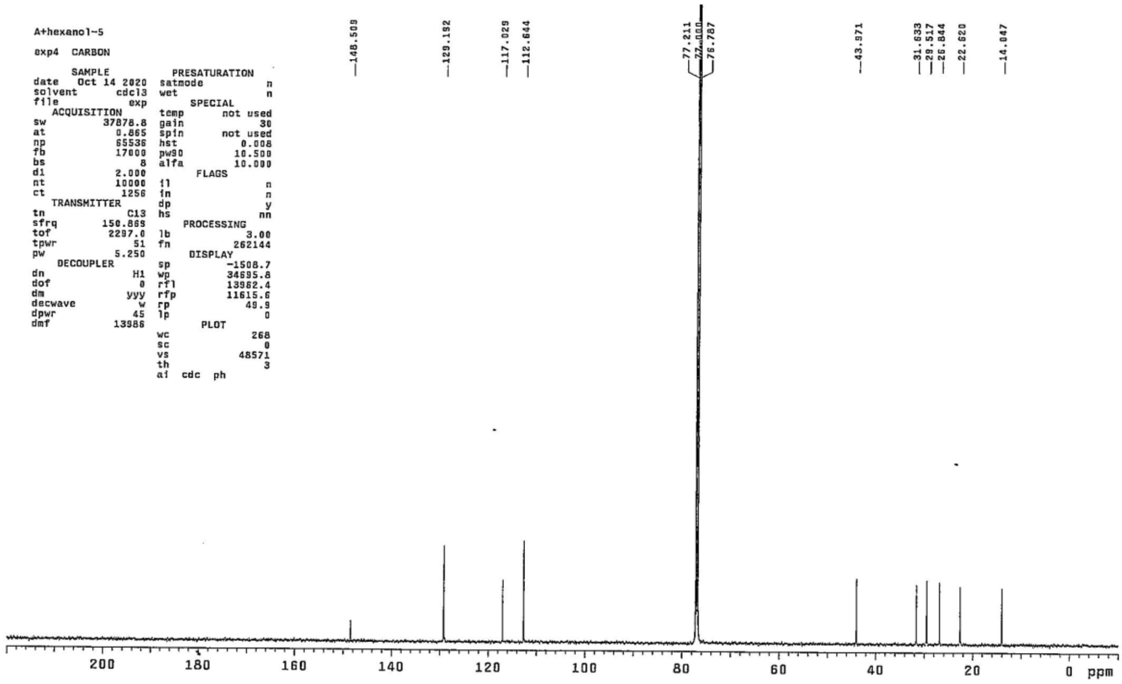

Figure S57. ^{13}C spectrum of 2q

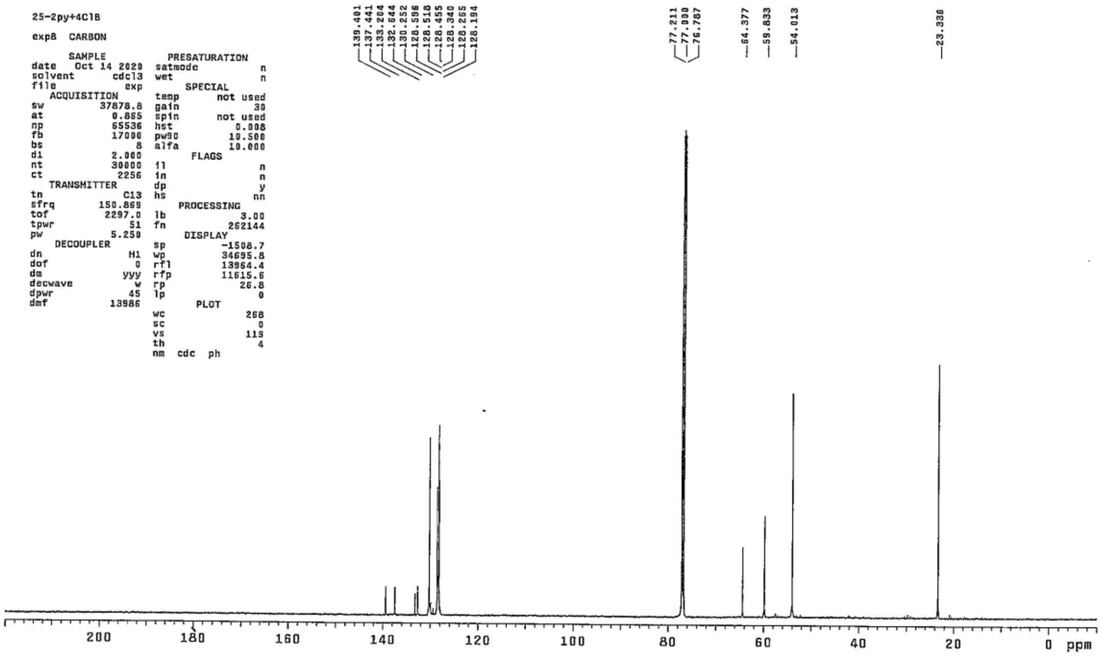

Figure S58. ^{13}C spectrum of 2r

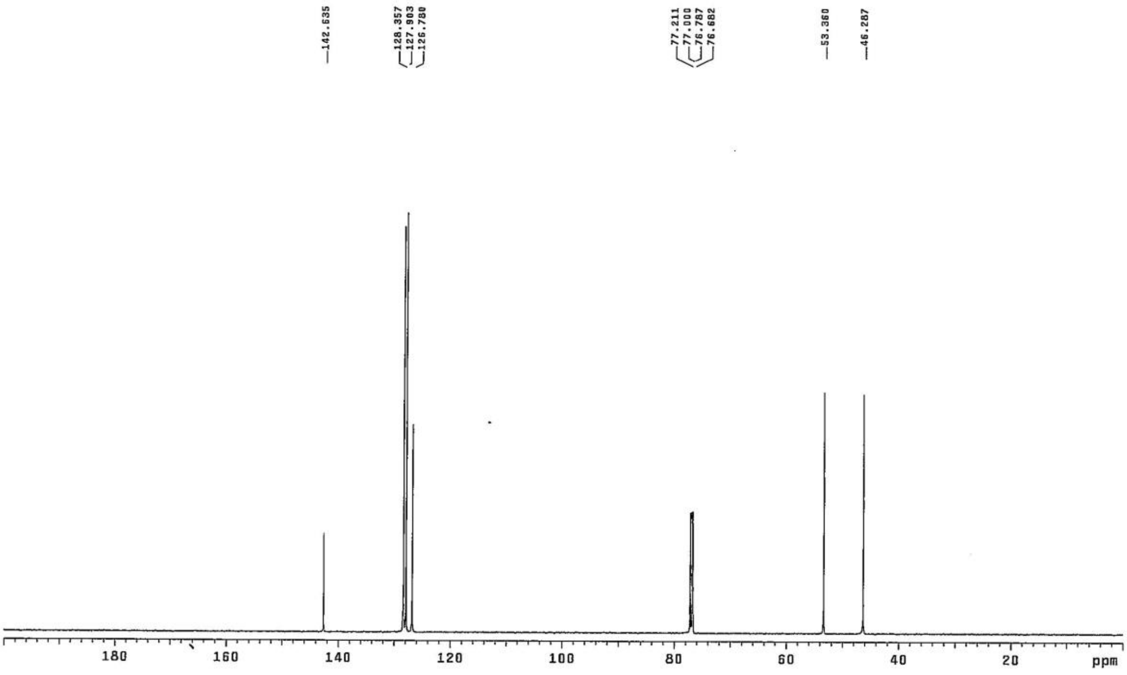

Figure S59. ^{13}C spectrum of 2s

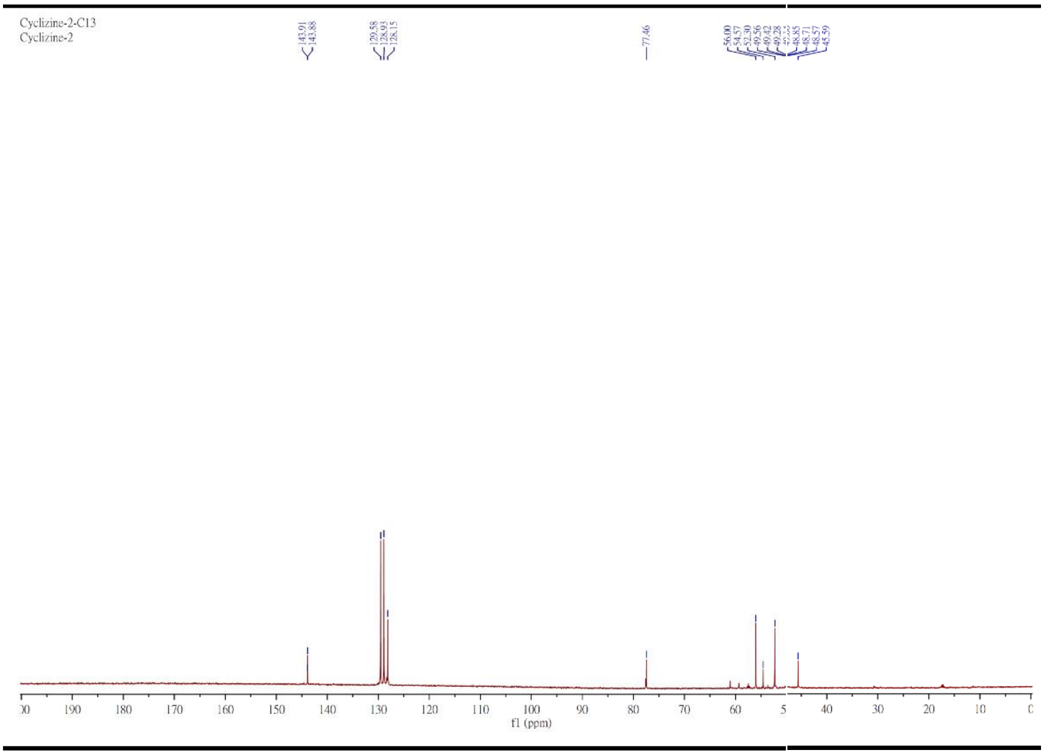

Figure S60. ^{13}C spectrum of cyclizine

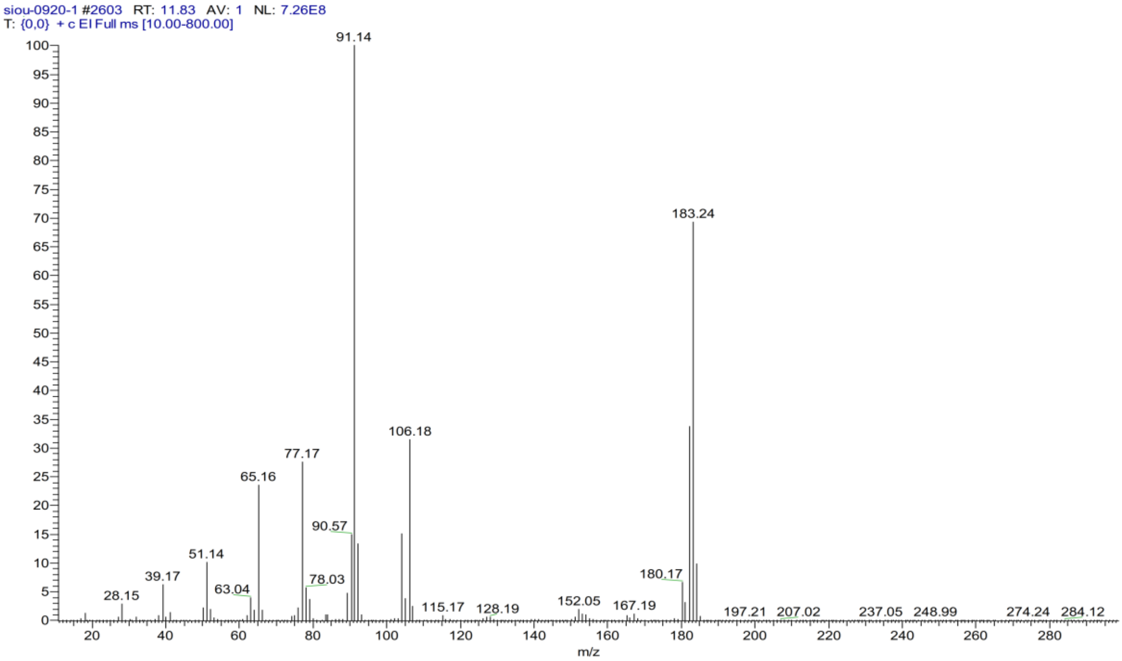

Figure S61. Mass spectrum of 2a

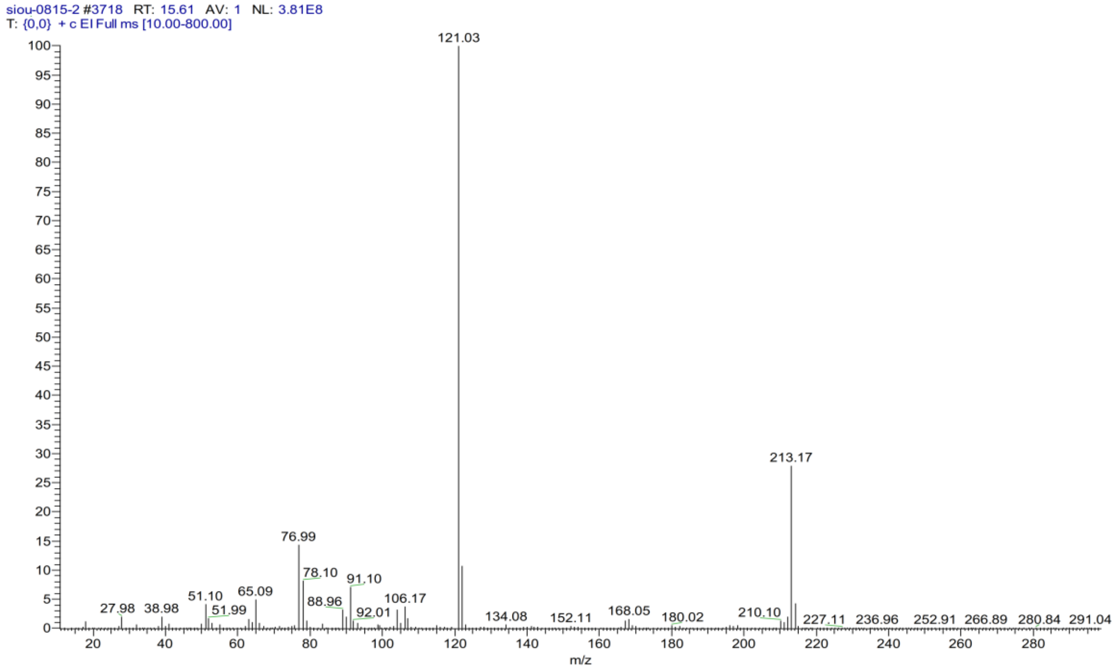

Figure S62. Mass spectrum of 2b

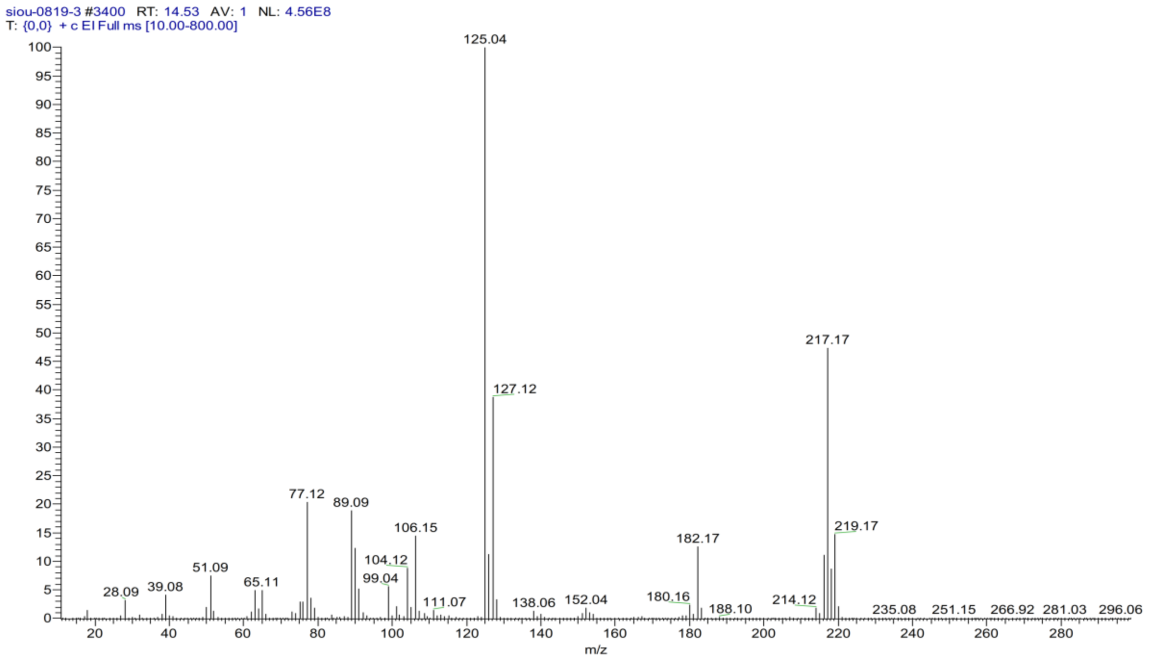

Figure S63. Mass spectrum of 2c

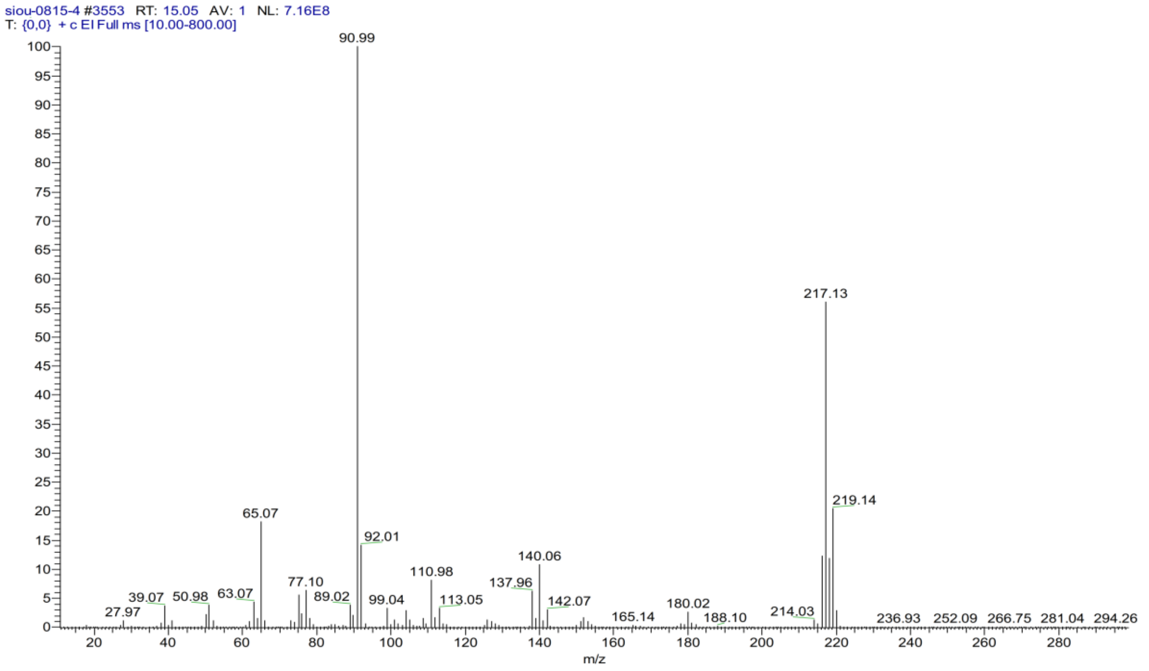

Figure S64. Mass spectrum of 2d

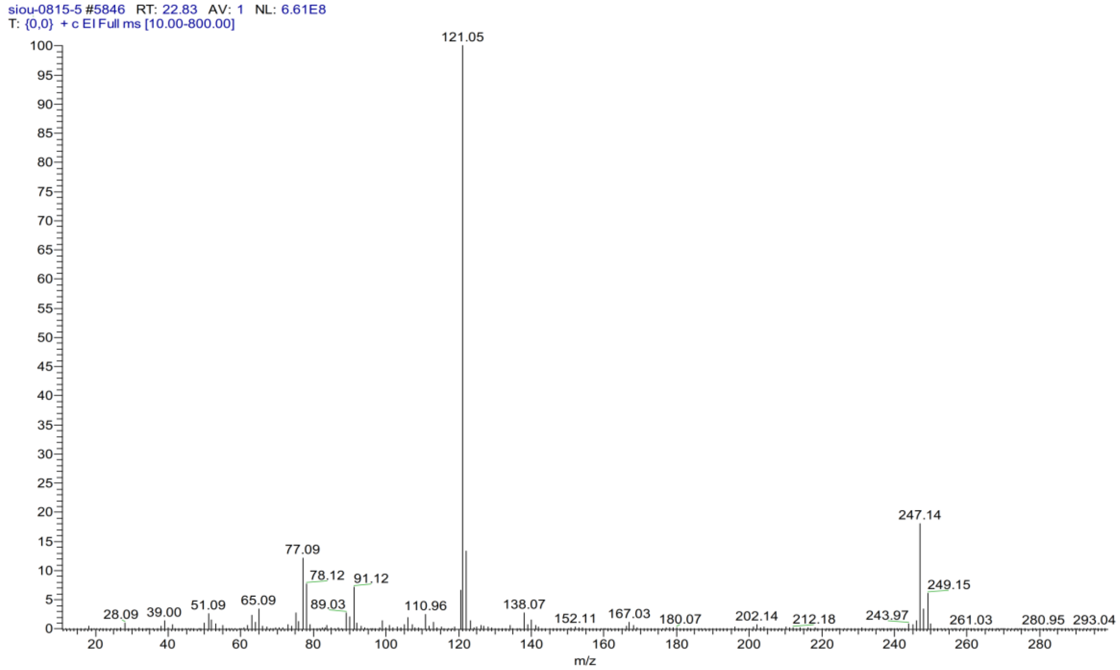

Figure S65. Mass spectrum of 2e

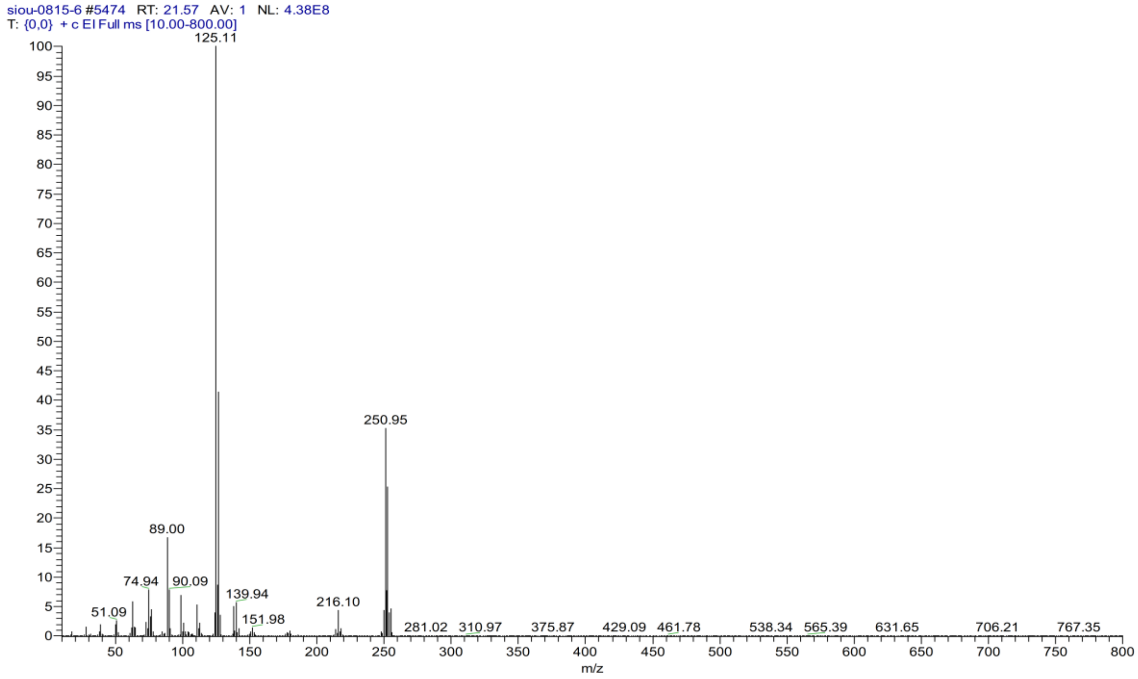

Figure S66. Mass spectrum of 2f

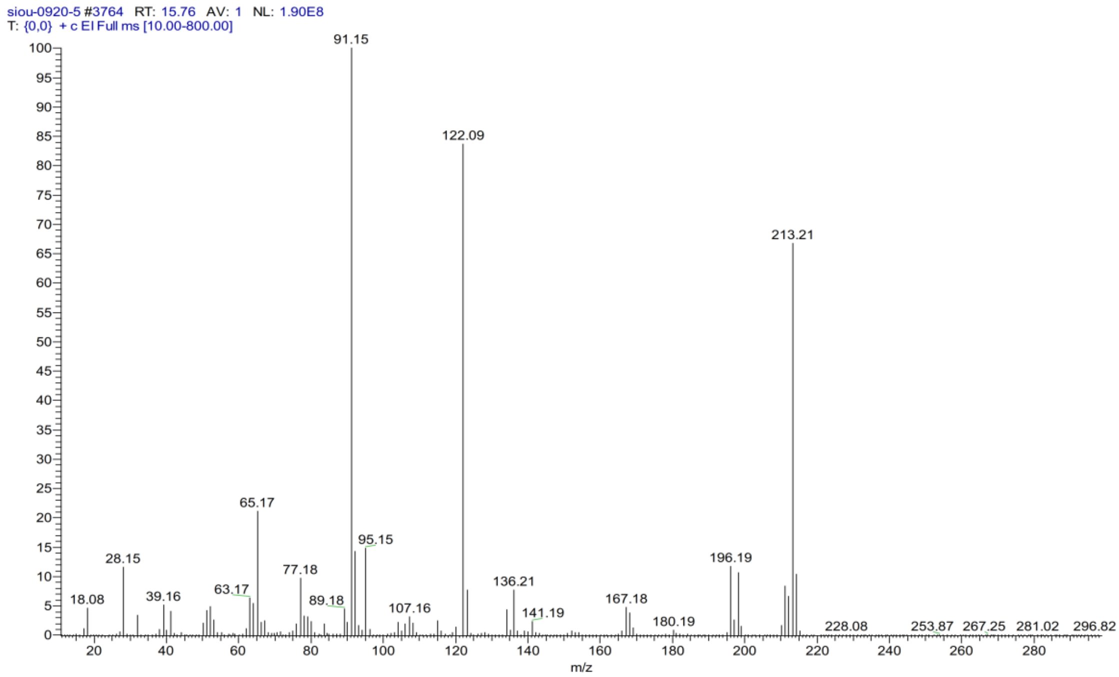

Figure S67. Mass spectrum of 2g

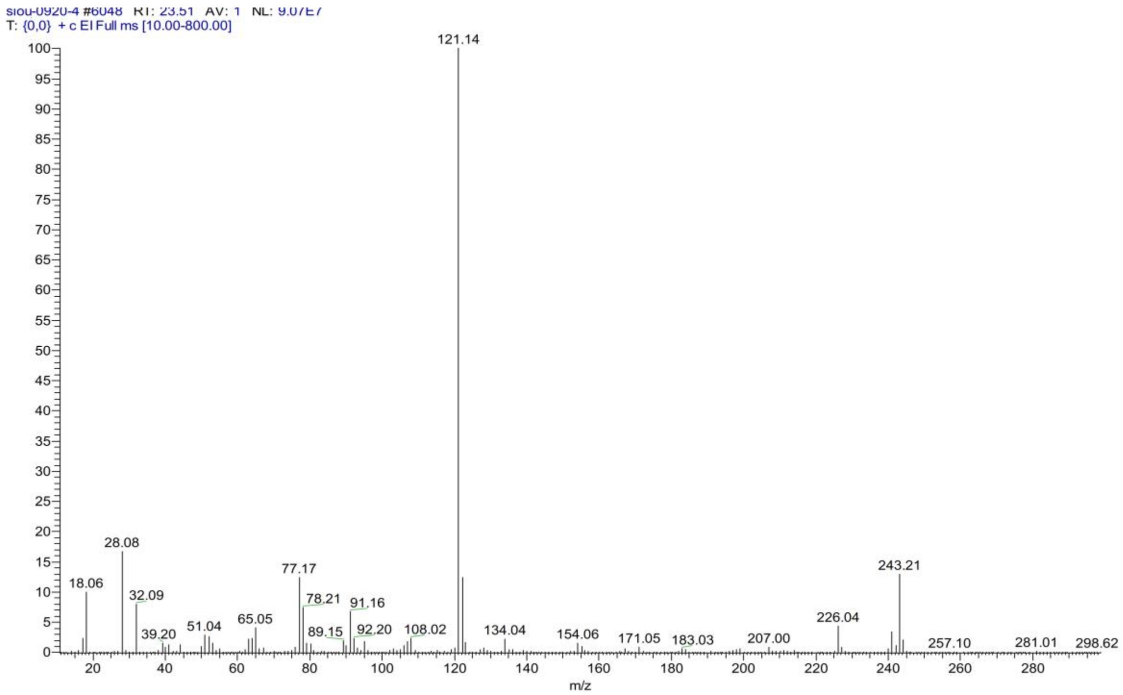

Figure S68. Mass spectrum of 2h

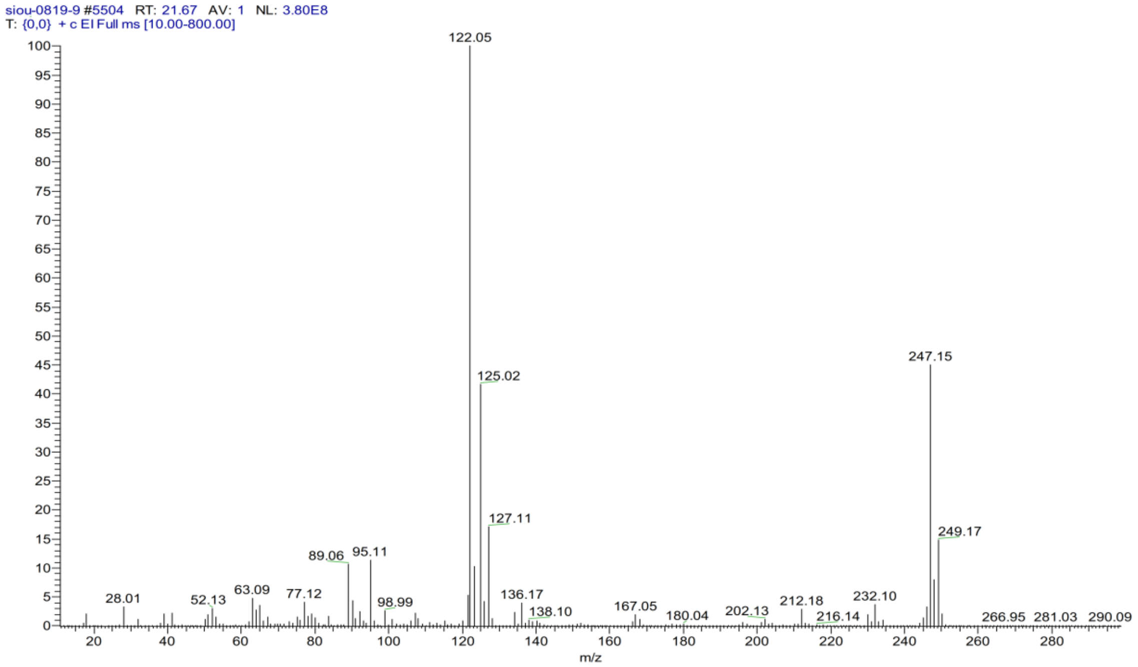

Figure S69. Mass spectrum of 2i

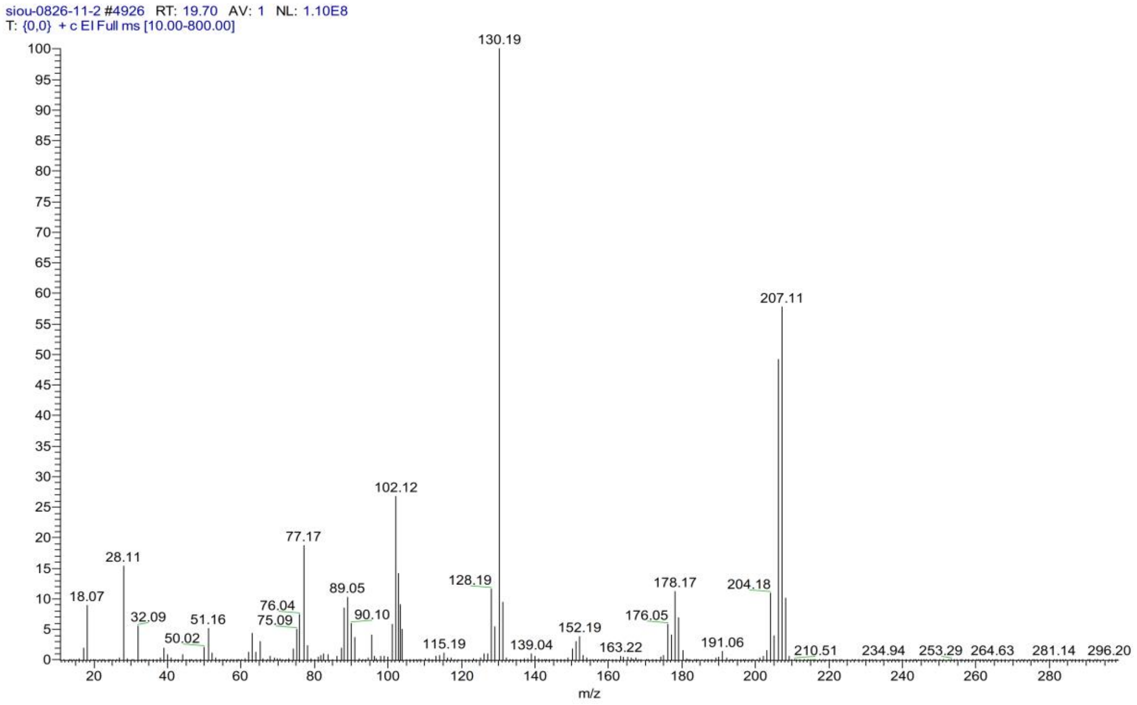

Figure S70. Mass spectrum of 2j

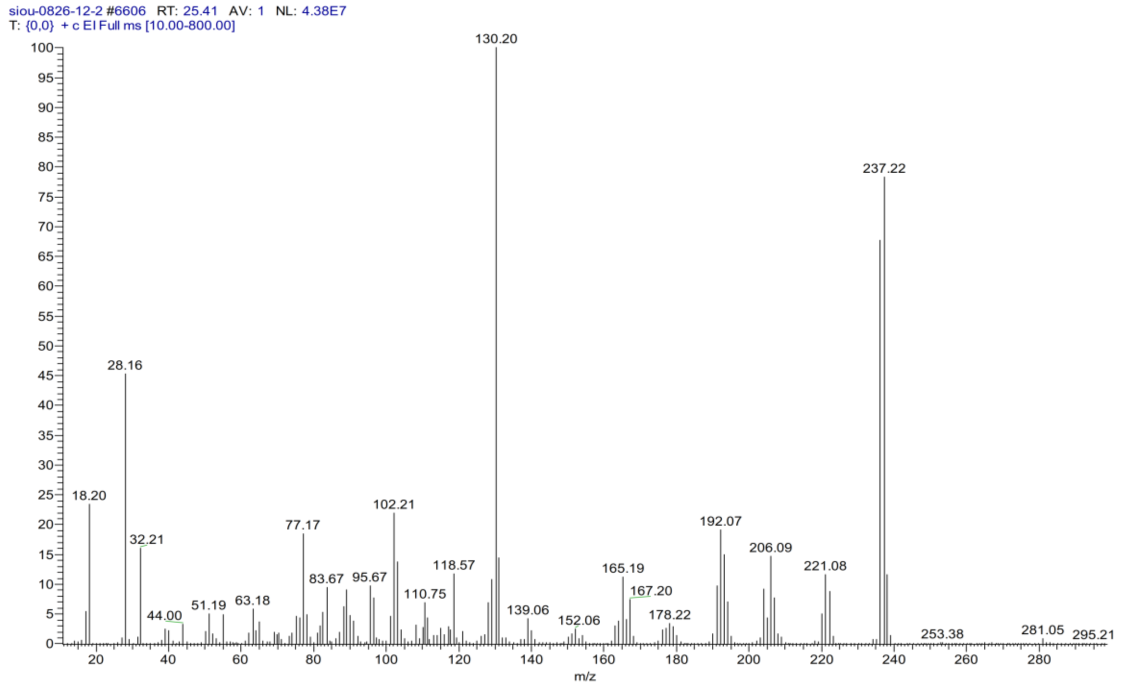

Figure S71. Mass spectrum of 2k

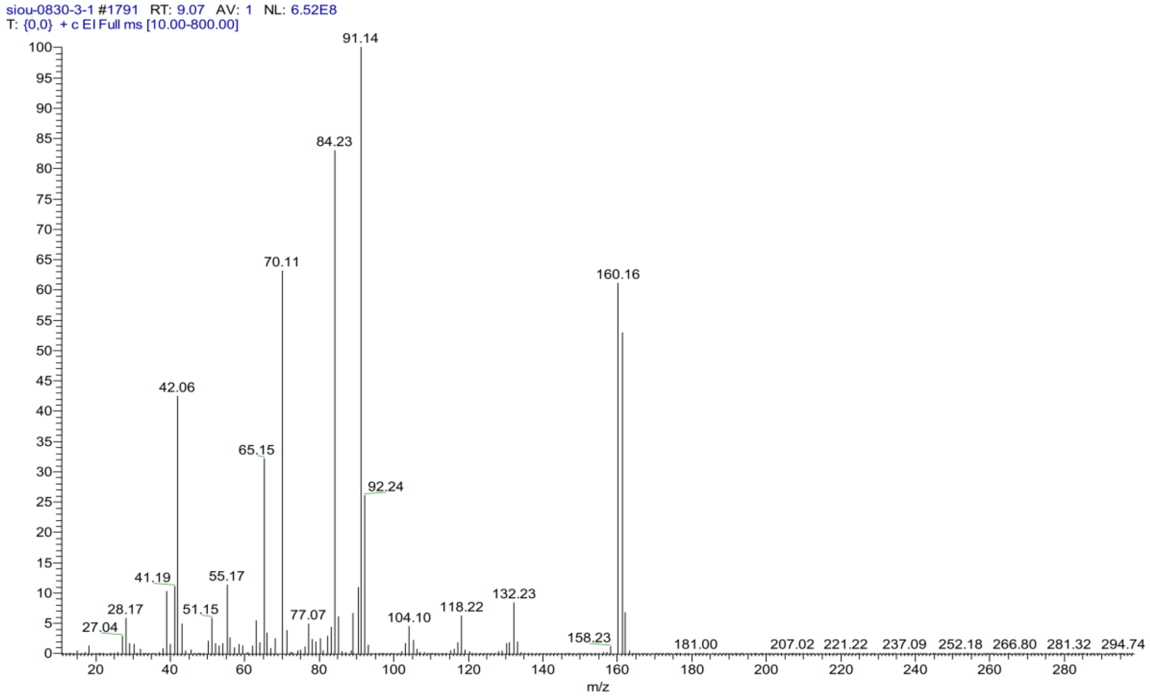

Figure S72. Mass spectrum of 2l

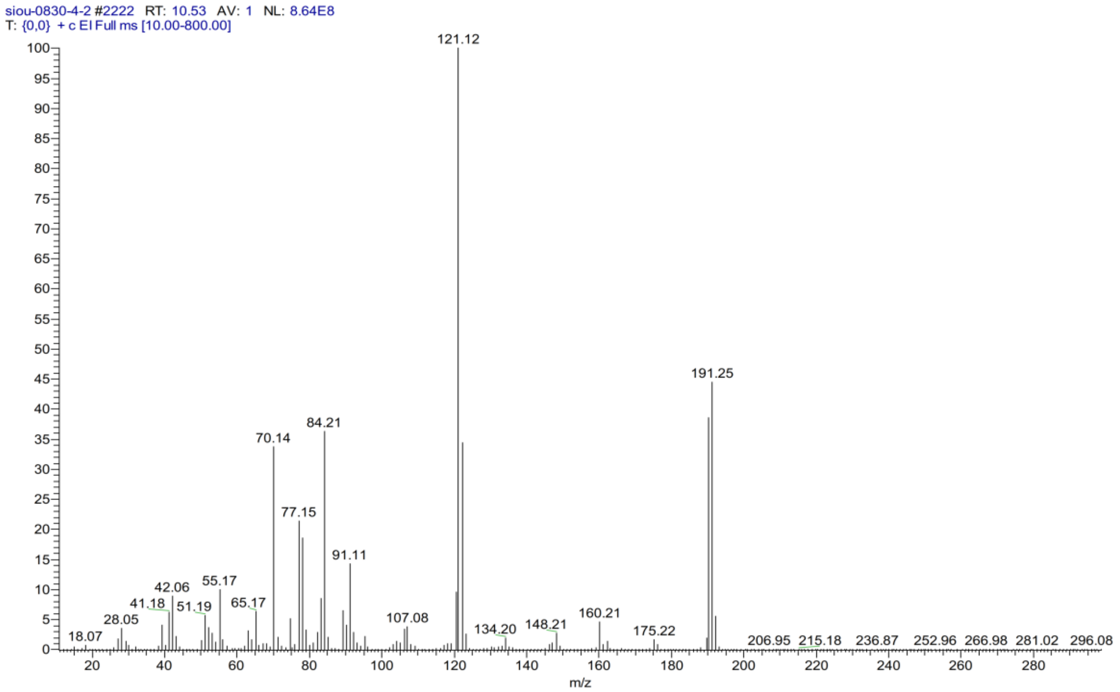

Figure S73. Mass spectrum of 2m

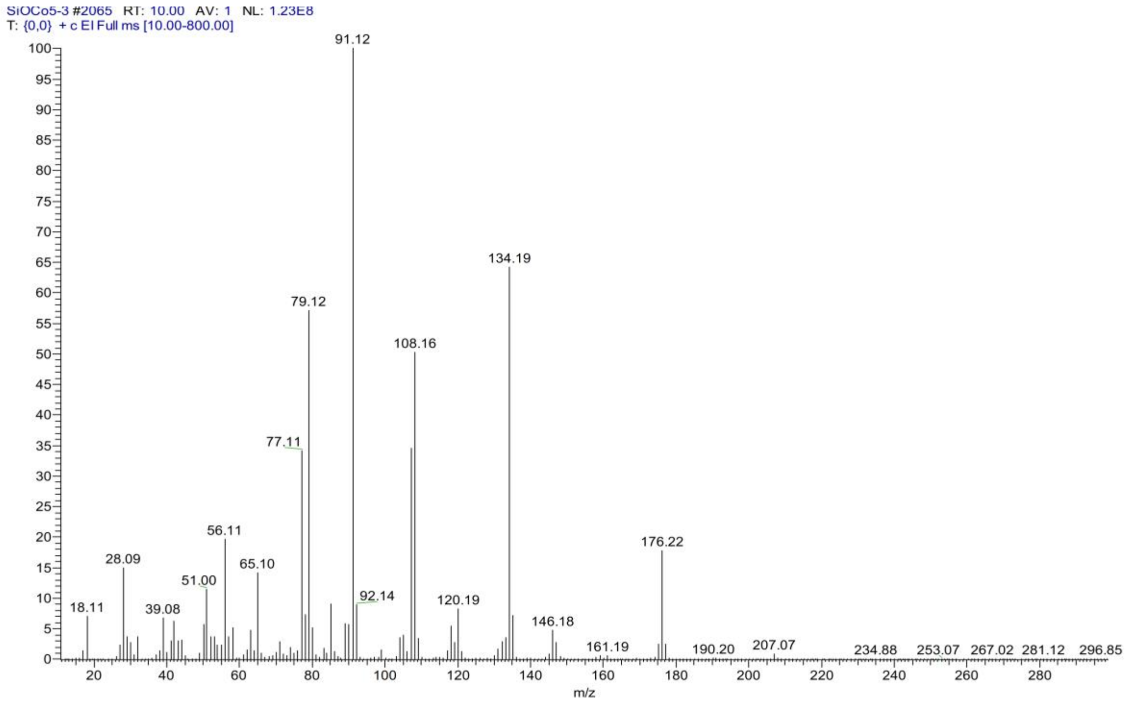

Figure S74. Mass spectrum of 2n

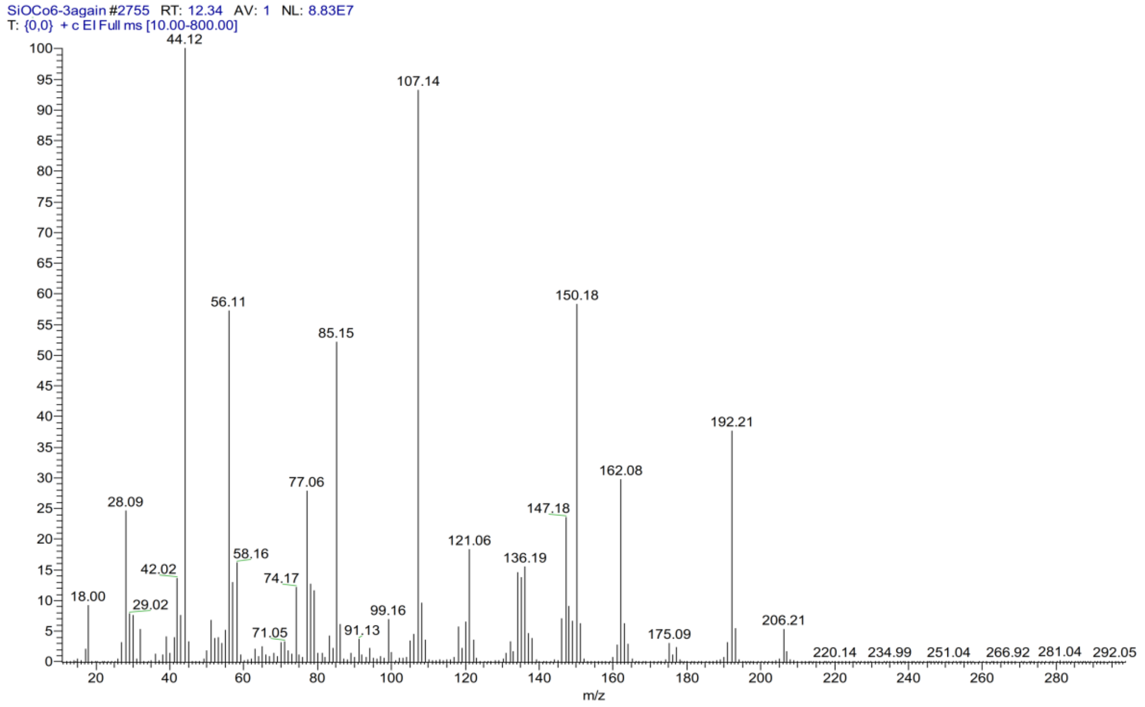

Figure S75. Mass spectrum of 2o

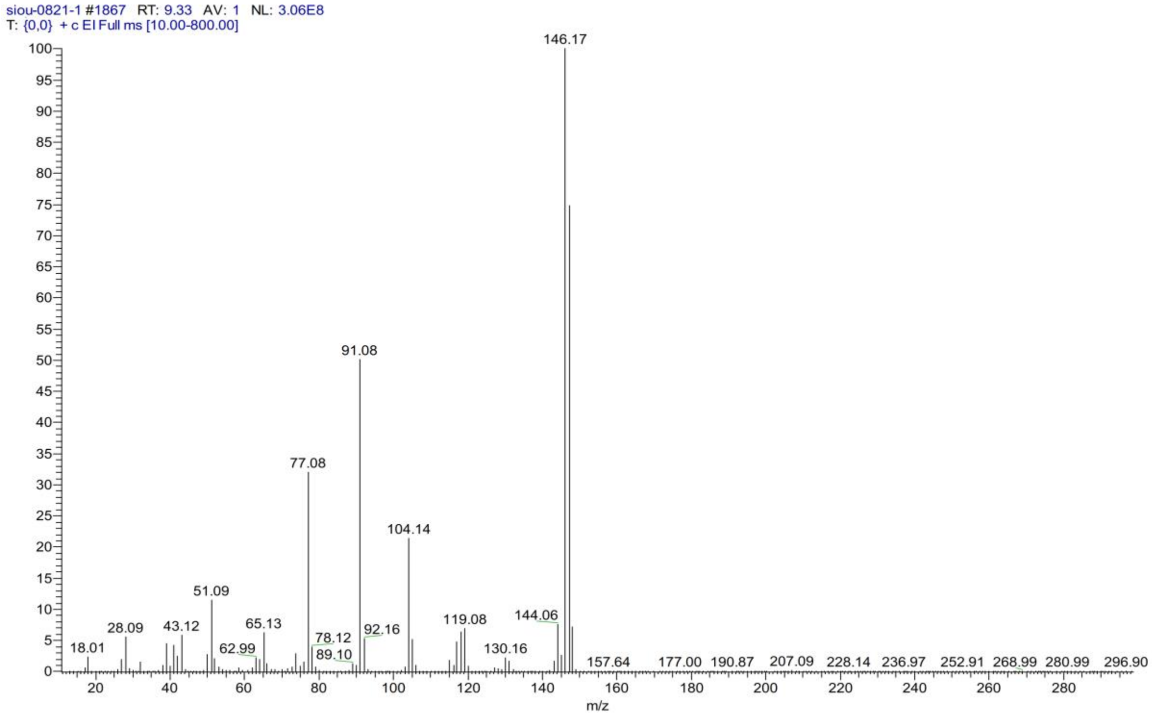

Figure S76. Mass spectrum of 2p

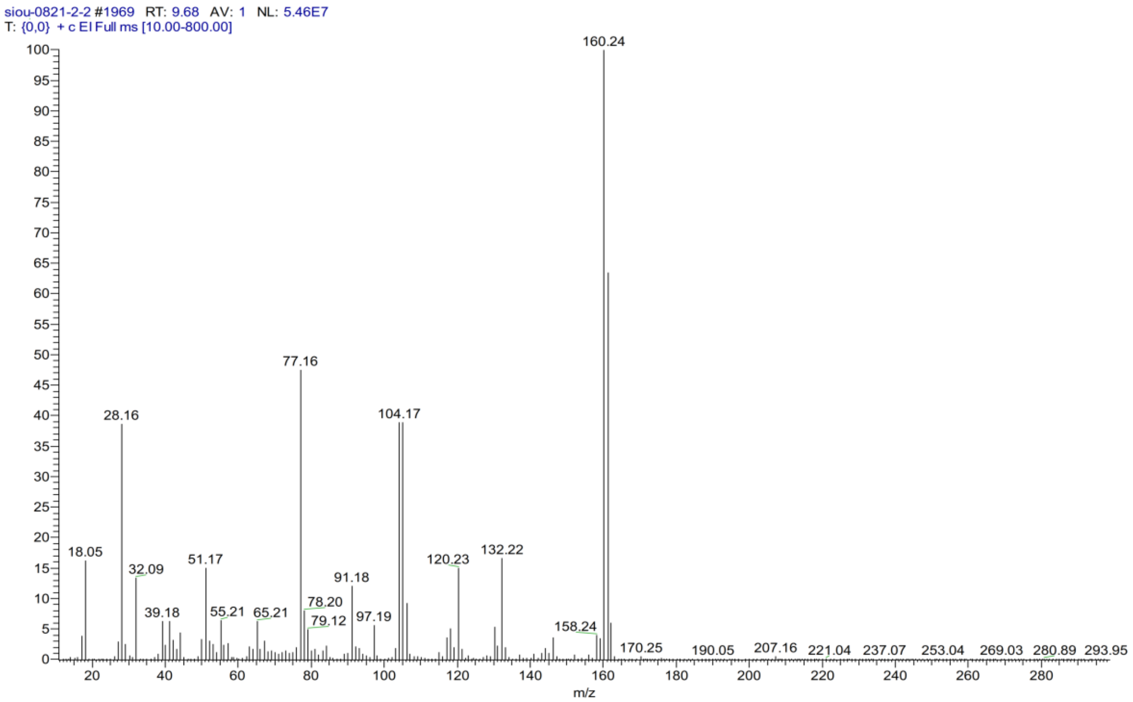

Figure S77. Mass spectrum of 2q

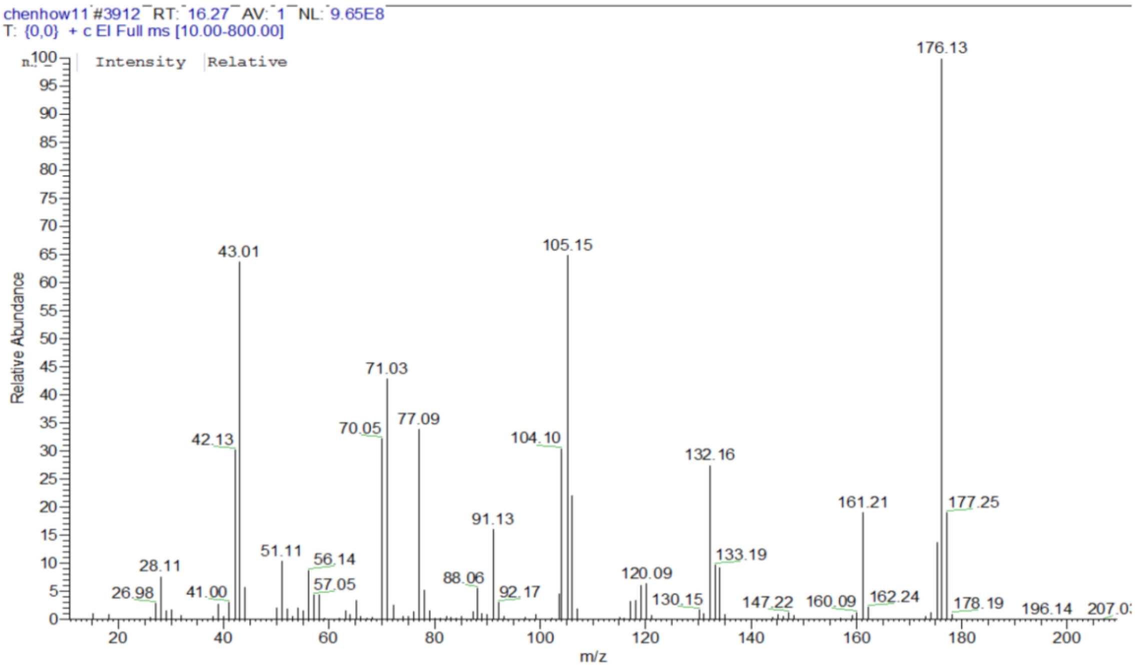

Figure S78. Mass spectrum of 2r

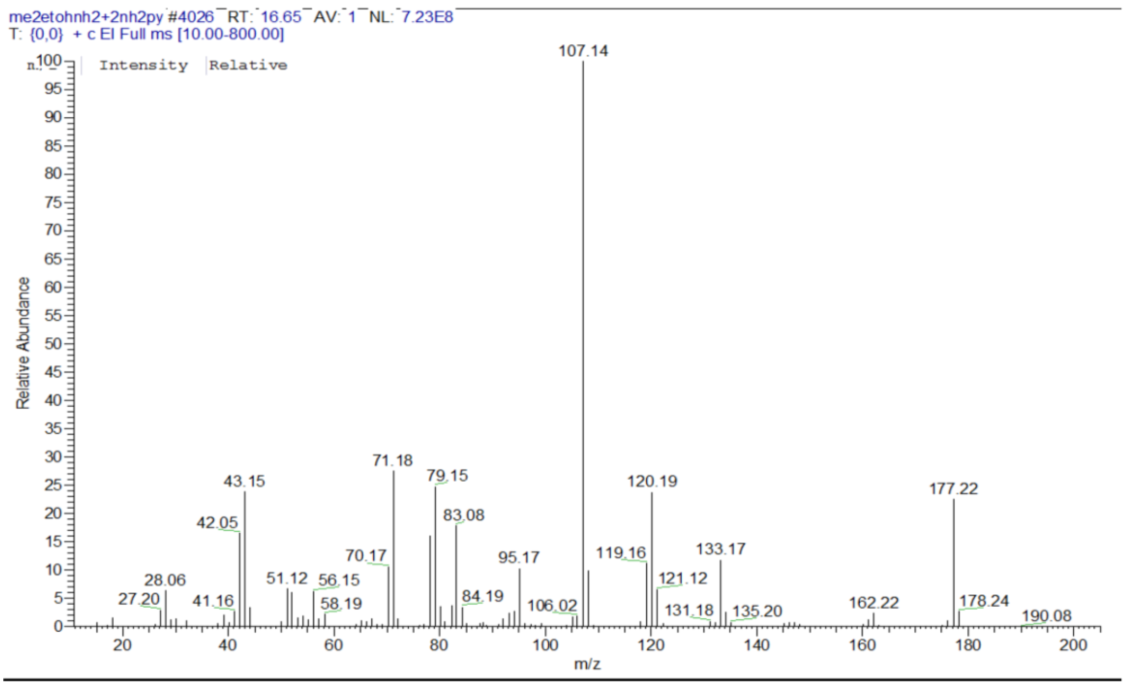

Figure S79. Mass spectrum of 2s

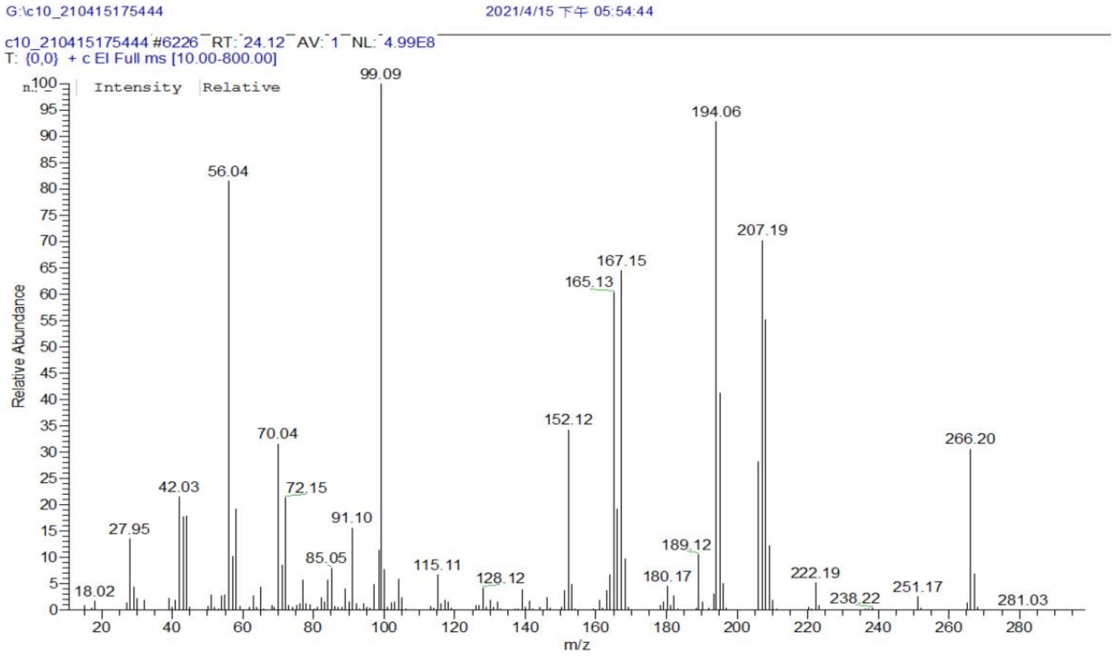

Figure S80. Mass spectrum of cyclizine
